# Deep learning in optical metrology: a review

**DOI:** 10.1038/s41377-022-00714-x

**Published:** 2022-02-23

**Authors:** Chao Zuo, Jiaming Qian, Shijie Feng, Wei Yin, Yixuan Li, Pengfei Fan, Jing Han, Kemao Qian, Qian Chen

**Affiliations:** 1 Smart Computational Imaging (SCI) Laboratory, Nanjing University of Science and Technology, 210094 Nanjing, Jiangsu Province China; 2 Jiangsu Key Laboratory of Spectral Imaging & Intelligent Sense, Nanjing University of Science and Technology, 210094 Nanjing, Jiangsu Province China; 3 School of Engineering and Materials Science, Queen Mary University of London, London, E1 4NS UK; 4 School of Computer Science and Engineering, Nanyang Technological University, Singapore, 639798 Singapore

**Keywords:** Imaging and sensing, Optical metrology

## Abstract

With the advances in scientific foundations and technological implementations, optical metrology has become versatile problem-solving backbones in manufacturing, fundamental research, and engineering applications, such as quality control, nondestructive testing, experimental mechanics, and biomedicine. In recent years, deep learning, a subfield of machine learning, is emerging as a powerful tool to address problems by learning from data, largely driven by the availability of massive datasets, enhanced computational power, fast data storage, and novel training algorithms for the deep neural network. It is currently promoting increased interests and gaining extensive attention for its utilization in the field of optical metrology. Unlike the traditional “physics-based” approach, deep-learning-enabled optical metrology is a kind of “data-driven” approach, which has already provided numerous alternative solutions to many challenging problems in this field with better performances. In this review, we present an overview of the current status and the latest progress of deep-learning technologies in the field of optical metrology. We first briefly introduce both traditional image-processing algorithms in optical metrology and the basic concepts of deep learning, followed by a comprehensive review of its applications in various optical metrology tasks, such as fringe denoising, phase retrieval, phase unwrapping, subset correlation, and error compensation. The open challenges faced by the current deep-learning approach in optical metrology are then discussed. Finally, the directions for future research are outlined.

## Introduction

Optical metrology is the science and technology of making measurements with the use of light as standards or information carriers^1–3^. Light is characterized by its fundamental properties, namely, amplitude, phase, wavelength, direction, frequency, speed, polarization, and coherence. In optical metrology, these fundamental properties of light are ingeniously utilized as information carriers of a measurand, enabling a wide range of optical metrology tools that allow the measurement of a wide range of subjects^4–6^. For example, optical interferometry takes advantage of the wavelength of light as a precise dividing marker of length. The speed of light defines the international standard of length, the meter, as the length traveled in vacuum during a time interval of 1/299,792,458 of a second^7^. As a result, optical metrology is being increasingly adopted in many applications where reliable data about the distance, displacement, dimensions, shape, roughness, surface properties, strain, and stress state of the object under test are required^8–10^. Optical metrology is a broad and interdisciplinary field relating to diverse disciplines such as photomechanics, optical imaging, and computer vision. There is no strict boundary between those fields, and in fact, the term “optical metrology” is often interchangeably used with “optical measurement”, in which achieving higher precision, sensitivity, repeatability, and speed is always a priority^11,12^.

There are a few inventions that revolutionized optical metrology. The first is the invention of laser^13,14^. The advent of laser interferometry could be traced back to experiments conducted independently in 1962 by Denisyuk^15^ and Leith and Upatnix^16^ with the objective of marrying coherent light produced by lasers with Gabor’s holography method^17^. The use of lasers as a light source in optical metrology marked the first time that such highly controlled light became available as a physical medium to measure the physical properties of samples, opening up new possibilities for optical metrology. The second revolution was initiated with the invention of charged coupled device (CCD) cameras in 1969, which replaced the earlier photographic emulsions by virtue of recording optical intensity signals from the measurand digitally^8^. The use of the CCD camera as a recording device in optical metrology represented another important milestone: the compatibility of light with electricity, i.e., “light” can be converted into “electrical quantity (current, voltage, etc.)”. This means that the computational storage, access, analysis, and transmission of captured data are easily attainable, leading to the “digital transition” of optical metrology. Computer-based signal processing tools were introduced to automate the quantitative determination of optical metrology data, eliminating the inconvenience associated with the manual, labor-intensive, time-consuming evaluation of fringe patterns^18–20^. Methods such as digital interferometry^21^, digital holography^22^, and digital image correlation (DIC)^23^ have become state of the art by now.

With the digital transition, image processing plays an essential role in optical metrology for the purpose of converting the observed measurements (generally displayed in the form of deformed fringe/speckle patterns) into the desired attributes (such as geometric coordinates, displacements, strain, refractive index, and others) of an object under study. Such information-recovery process is similar to those of computer vision and computational imaging, presenting as an inverse problem that is often ill-posed with respect to the existence, uniqueness, and stability of the solution^24–27^. Tremendous progress has been achieved in terms of accurate mathematical modeling (statistical models of noise and the observational data)^28^, regularization techniques^29^, numerical methods, and their efficient implementations^30^. For the field of optical metrology, however, the situation becomes quite different due to the fact that the optical measurements are frequently carried out in a highly controlled environment. Instead of explicitly interpreting optical metrology tasks from the perspective of solving inverse problems (based on a formal optimization framework), mainstream scientists in optical metrology prefer to bypass the ill-posedness and simplify the problem by means of active strategies, such as sample manipulation, system adjustment, and multiple acquisitions^31^. A typical example is the phase-shifting technique^32^, which sacrifices the time and effort of capturing multiple fringe patterns to exchange for a deterministic and straightforward solution. Under such circumstances, the phase retrieval problem is well-posed or even over-determined (when the phase-shifting step is larger than 3), and employing more evolved algorithms, such as compressed sensing^33^ and nonconvex (low-rank) regularization^34^ seem redundant and unnecessary, especially as they fail to demonstrate clear advantages over classical ones in terms of accuracy, adaptability, speed, and, more importantly, ease-of-use. This gives us the key question and motivation of this review paper: whether machine learning will be the driving force in optical metrology not only provides superior solutions to the growing new challenges but also tolerates imperfect measurement conditions with the least efforts, such as additive noise, phase-shifting error, intensity nonlinearity, motion, and vibration.

In the past few years, we have indeed witnessed the rapid progress on high-level artificial intelligence (AI), where deep representations based on convolutional and recurrent neural network models are learned directly from the captured data to solve many tasks in computer vision, computational imaging, and computer-aided diagnosis with unprecedented performance^35–37^. The early framework for deep learning was established on artificial neural networks (ANNs) in the 1980s^38^, yet only recently the real impact of deep learning became significant due to the advent of fast graphics processing units (GPUs) and the availability of large datasets^39^. In particular, deep learning has revolutionized the computer vision community, introducing non-traditional and effective solutions to numerous challenging problems such as object detection and recognition^40^, object segmentation^41^, pedestrian detection^42^, image super-resolution^43^, as well as medical image-related applications^44^. Similarly, in computational imaging, deep learning has led to rapid growth in algorithms and methods for solving a variety of ill-posed inverse computational imaging problems^45^, such as super-resolution microscopy^46^, lensless phase imaging^47^, computational ghost imaging^48^, and image through scattering media^49^. In this context, researchers in optical metrology have also made significant explorations in this regard with very promising results within just a few short years, as evidenced by the ever-increasing and the respectable number of publications^50–55^. Meanwhile, those research works are scattered rather than systematic, which gives us the second motivation to provide a comprehensive review to understand their principles, implementations, advantages, applications, and challenges. It should be noted that optical metrology covers a wide range of methods and applications today. It would be beyond the scope of this review to discuss all relevant technologies and trends. We, therefore, restrict our focus to phase/correlation measurement techniques, such as interferometry, holography, fringe projection, and DIC. Although phase retrieval and wave-field sensing technologies, such as defocus variation (Gerchberg–Saxton–Fienup-type methods^56,57^), transport of intensity equation (TIE)^58,59^, aperture modulation^60^, ptychography^61,62^, and wavefront sensing (e.g., Shack–Hartmann^63^, Pyramid^64^, and computational shear interferometry^65^), has been recently introduced to optical metrology^66–68^, they may be more appropriately placed in the field of “computational imaging”. The reader is referred to the earlier review by Barbastathis et al.^45^ for more detailed information on this topic. It is also worth mentioning that (passive) stereovision, which extracts depth information from stereo images, is an important branch of photogrammetry that has been extensively studied by the computer vision community. Although stereovision techniques do not strictly fall into the category of optical metrology, due to the fact that many ideas and algorithms in DIC and fringe projection were “borrowed” from stereovision, they are also included in this review.

The remainder of this review is organized as follows. We start by summarizing the relevant foundations and image formation models of different optical metrology approaches, which are generally required as a priori knowledge in conventional optical metrology methods. Next, we present a general hierarchy of the image-processing algorithms that are most commonly used in conventional optical metrology in the “Image processing in optical metrology” section. After a brief introduction to the history and basic concept of deep learning, we recapitulate the advantages of using deep learning in optical metrology tasks by interpreting the concept as an optimization problem. We then present a recollection of the deep learning methods that have been proposed in optical metrology, suggesting the pervasive penetration of deep learning in almost all aspects of the image-processing hierarchy. The “Challenges” section discusses both technical and implementation challenges faced by the current deep-learning approach in optical metrology. In the “Future directions” section, we give our outlook for the prospects for deep learning in optical metrology. Finally, conclusions and closing remarks are given in the “Conclusions” section.

## Image formation in optical metrology

**Fig. 1:**
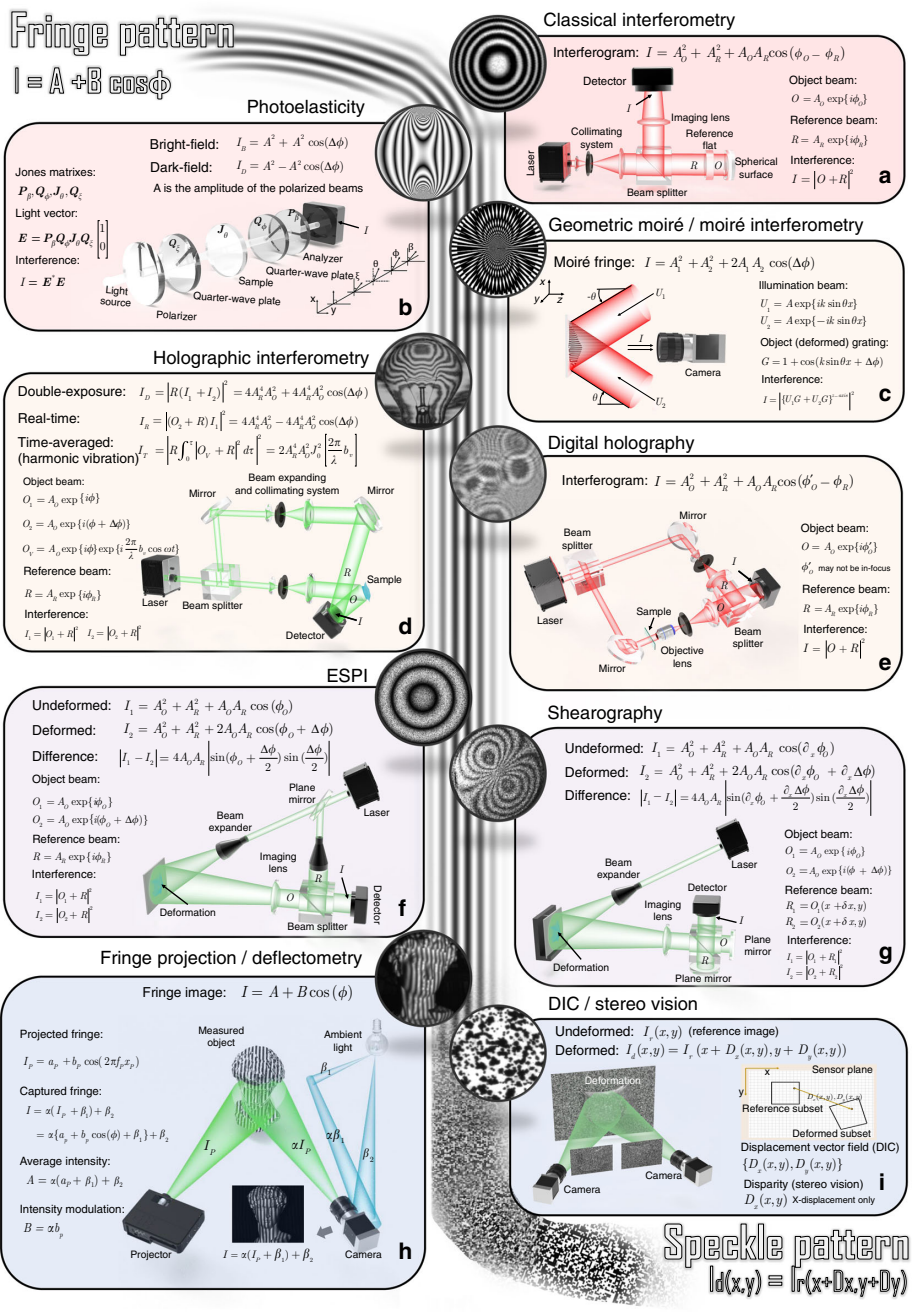
Image formation in typical optical metrology methods. **a** Classical interferometry. **b** Photoelasticity. **c** Geometric moiré and moiré interferometry. **d** Holographic interferometry. **e** Digital holography. **f** Electronic speckle shearing interferometry (ESPI). **g** Shearography. **h** Fringe projection profilometry (FPP) and deflectometry. **i** Digital image correlation (DIC) and stereovision

Optical metrology methods often form images (e.g., fringe/speckle patterns) for processing. Thus image formation is essential to reconstruct various quantities. In most interferometric metrological methods, the image is formed by the coherent superposition of the object and reference beams. As a result, the raw intensity across the object is modulated by a harmonic function, resulting in the bright and dark contrasts, known as fringe patterns. A typical fringe pattern can be written as^18,19^

1 $$I\left( {x,y} \right) = A\left( {x,y} \right) + B\left( {x,y} \right)\cos \left[ {\phi \left( {x,y} \right)} \right]$$

where (*x*, *y*) refers to the spatial coordinates along the horizontal and vertical directions, *A*(*x*, *y*) is the background intensity, *B*(*x*, *y*) is the fringe amplitude, *ϕ*(*x*, *y*) is the phase distribution. In most cases, phase is the primary quantity of the fringe pattern to be retrieved as it is related to the final object quantities of interest, such as surface shape, mechanical displacement, 3D coordinates, and their derivations. The related techniques include classical interferometry, photoelasticity, holographical interferometry, digital holography, etc. On a different note, the fringe patterns can also be created noninterferometrically by overlapping of two periodic gratings as in geometric moiré, or incoherent projection of structured patterns onto the object surface as in fringe projection profilometry (FPP)/deflectometry. As summarized in Fig. 1, though the final fringe patterns obtained in all forms of fringe-based techniques discussed herein are similar in form, the physics behind the image formation process and the meanings of the fringe parameters are different. In DIC, the measured intensity images are speckle patterns of the specimen surface before and after deformation,

2 $$I_d\left( {x,y} \right) = I_r\left( {x + D_x(x,y),y + D_y(x,y)} \right)$$

where $\left( {D_x(x,y),D_y(x,y)} \right)$ refers to the displacement vector-field mapping from the undeformed/reference pattern *I*_*r*_(*x*, *y*) to the deformed one *I*_*d*_(*x*, *y*). It directly provides full-field displacements and strain distributions of the sample surface. The DIC technique can also be combined with binocular stereovision or stereophotogrammetry to recover depth and out-of-plane deformation of the surface from the displacement field (so-called disparity) by exploiting the unique textures present in two or more images of the object taken from different viewpoints. The image formation processes for typical optical metrology methods are briefly described as follows.

1. **Classical interferometry:** In classical interferometry, the fringe pattern is formed by superimposition of two smooth coherent wavefronts, one of which is typically a flat or spherical reference wavefront and the other a distorted wavefront formed and directed by optical components^69,70^ (Fig. 1a). The phase of the fringe pattern reflects the difference between the ideal reference wavefront and object wavefront. Typical examples of classical interferometry include the use of configurations such as the Michelson, Fizeau, Twyman Green, and Mach-Zehnder interferometers to characterize the surface, aberration, or roughness of optical components with high accuracy, of the order of a fraction of the wavelength.
2. **Photoelasticity**: Photoelasticity is a nondestructive, full-field, optical metrology technique for measuring the stress developed in transparent objects under loading^71,72^. Photoelasticity is based on an optomechanical property, so-called “double refraction” or “birefringence” observed in many transparent polymers. Combined with two circular polarizers (linear polarizer coupled with quarter waveplate) and illuminated with a conventional light source, a loaded photoelastic sample (or photoelastic coating applied to an ordinary sample) can produce fringe patterns whose phases are associated with the difference between the principal stresses in a plane perpendicular to the light propagation direction^73^ (Fig. 1b).
3. **Geometric moiré/Moiré interferometry**: In optical metrology, the moiré technique is defined as the utilization of the moiré phenomenon to measure shape, deformation, or displacements of surfaces^74,75^. A moiré pattern is formed by the superposition of two periodic or quasi-periodic gratings. One of these gratings is called reference grating, and the other one is object grating mounted or engraved on the surface to be studied, which is subjected to distortions induced by surface changes. For in-plane displacement and strain measurements, moiré technology has evolved from low-sensitivity geometric moiré^75–77^ to high-sensitivity moiré interferometry^75,78^. In moiré interferometry, two collimated coherent beams interfere to produce a virtual reference grating with high frequencies, which interacts with the object grating to create the moiré pattern with fringes representing subwavelength in-plane displacements per contour (Fig. 1c).
4. **Holographic interferometry**: Holography, invented by Gabor^17^ in the 1940 s, is a technique that records an interference pattern and uses diffraction to reproduce a wavefront, resulting in a 3D image that still has the depth, parallax, and other properties of the original scene. The principle of holography can also be utilized as an optical metrology tool. In holographic interferometry, a wavefront is first stored in the hologram and later interferometrically compared with another, producing fringe patterns that yield quantitative information about the object surface deriving these two wavefronts^79,80^. This comparison can be made in three different ways that constitute the basic approaches of holographic interferometry: real-time^81^, double-exposure^82^, and time-average holographic interferometry^83,84^ (Fig. 1d), allowing for both qualitative visualization and quantitative measurement of real-time deformation and perturbation, changes of the state between two specific time points, and vibration mode and amplitude, respectively.
5. **Digital holography:** Digital holography utilizes a digital camera (CMOS or CCD) to record the hologram produced by the interference between a reference wave and an object wave emanating from the sample^85,86^ (Fig. 1e). Unlike classical interferometry, the sample may not be precisely in-focus and can even be recorded without using any imaging lenses. The numerical propagation using Fresnel transform or angular spectrum algorithm enables digital refocusing at any depths of the sample without physically moving it. In addition, digital holography also provides an alternative and much simpler way to realize double-exposure^87^ and time-averaged holographic interferometry^88,89^, without additional benefits of quantitative evaluation of holographic interferograms and flexible phase-aberration compensation^86,90^.
6. **Electronic speckle pattern interferometry (ESPI)**: In ESPI, the tested object generally has an optically rough surface. When illuminated by a coherent laser beam, it will create a speckle pattern with random phase, amplitude, and intensity^91,92^. If the object is displaced or deformed, the object-to-image distance will change, and the phase of the speckle pattern will change accordingly. In ESPI, two speckle patterns are acquired one each for the undeformed and deformed states, by double exposure, and the absolute difference between these two deformed patterns results in the form of fringes superimposed on the speckle pattern where each fringe contour normally represents a displacement of half a wavelength (Fig. 1f).
7. **Electronic speckle shearing interferometry (shearography):** Electronic speckle shearing interferometry, commonly known as shearography, is an optical measurement technique similar to ESPI. However, instead of using a separate known reference beam, shearography uses the test object itself as the reference; and the interference pattern is created by two sheared speckle fields originated from the light scattered by the surface of the object under test^93,94^. In shearography, the phase encoded in the fringe pattern depicts the derivatives of the surface displacements, i.e., to the strain developed on the object surface (Fig. 1g). Consequently, the anomalies or defects on the surface of the object can be revealed more prominently, rendering shearography one of the most powerful tools for nondestructive testing applications.
8. **Fringe projection profilometry/deflectometry:** Fringe projection is a widely used noninterferometic optical metrology technique for measuring the topography of an object at a certain angle between the observation and the projection point^95,96^. The sinusoidal pattern in fringe projection techniques is generally incoherently formed by a digital video projector and directly projected onto the object surface. The corresponding distorted fringe pattern is recorded by a digital camera. The average intensity and intensity modulation of the captured fringe pattern are associated with the surface reflectivity and ambient illuminations, and the phase is associated with the surface height^32^ (Fig. 1h). Deflectometry is another structured light technique similar to FPP, but instead of being produced by a projector, similar types of fringe patterns are displayed on a planar screen and distorted by the reflective (mirror-like) test surface^97,98^. The phase measured in deflectometry is directly sensitive to the surface slope (similar to shearography), so it is more effective for detecting shape defects^99,100^.
9. **Digital image correction (DIC)/stereovision**: DIC is another important noninterferometic optical metrology method that employs image correlation techniques for measuring full-field shape, displacement, and strains of an object surface^23,101,102^. Generally, the object surface should have a random intensity distribution (i.e., a random speckle pattern), which distorts together with the sample surface as a carrier of deformation information. Images of the object at different loadings are captured with one (2D-DIC)^23^, or two synchronized cameras (3D-DIC)^103^, and then these images are analyzed with correlation-based matching (tracking or registration) to extract full-field displacement and strain distributions (Fig. 1i). Unlike 2D-DIC that is limited to in-plane deformation measurement of nominal planar objects, 3D-DIC, also known as stereo-DIC, allows for the measurement of 3D displacements (both in-plane and out-of-plane) for both planar and curved surfaces^104,105^. 3D-DIC is inspired by binocular stereovision or stereophotogrammetry in the computer vision community, which recovers the 3D coordinates by finding pixel correspondence (i.e., disparity) of unique features that exist in two or more images of the object taken from different points of view^106,107^. Nevertheless, unlike DIC, in which the displacement vector can be along both *x* and *y* directions, in stereophotogrammetry, after epipolar rectification, disparities between the images are along the *x* direction only^108^.

## Image processing in optical metrology

**Fig. 2:**
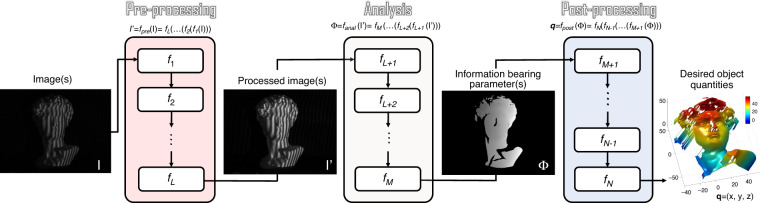
Image-processing pipeline of typical optical metrology methods. The pipeline of a typical optical metrology method (e.g., FPP) encompasses a sequence of distinct operations (algorithms) to process and analyze the image data, which can be further categorized into three main steps: pre-processing (e.g., denoising, image enhancement), analysis (e.g., phase demodulation, phase unwrapping), and postprocessing (e.g., phase-depth mapping)

**Fig. 3:**
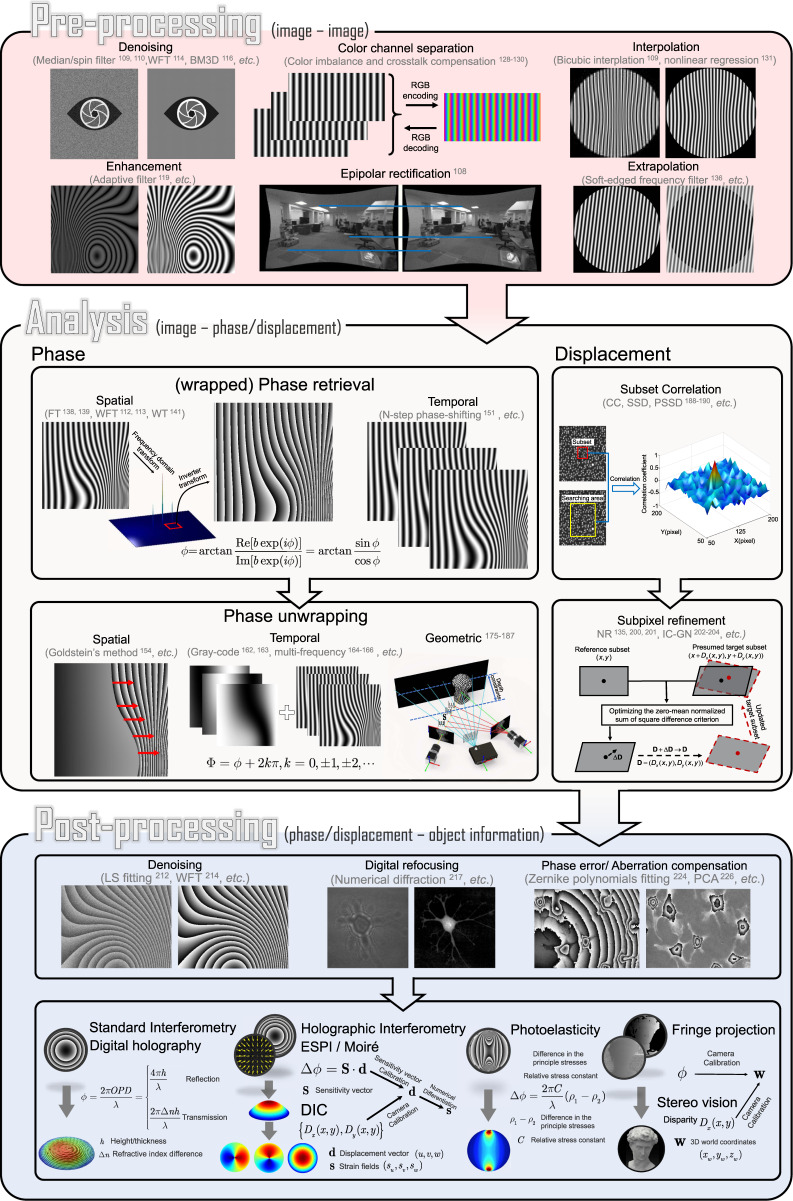
Hierarchy and typical algorithms of image processing in optical metrology. Image processing in optical metrology is not a one-step procedure. Depending on the purpose of the evaluation, a logical hierarchy of processing steps should be implemented before the desired information can be extracted from the image. In general, the image processing architecture in optical metrology consists of three main steps: pre-processing, analysis, and post-processing.

The elementary task of digital image processing in optical metrology can be defined as the conversion of the captured raw intensity image(s) into the desired object quantities taking into account the physical model of the intensity distribution describing the image formation process. In most cases, image processing in optical metrology is not a one-step procedure, and a logical hierarchy of image processing steps should be accomplished. As illustrated in Fig. 2, the image-processing hierarchy typically encompasses three main steps, pre-processing, analysis, and postprocessing, each of which includes a series of mapping functions that are cascaded to form a pipeline structure. For each operation, the corresponding *f* is an operator that transforms the image-like input into an output of corresponding (possibly resampled) spatial dimensions. Figure 3 shows the big picture of the image-processing hierarchy with various types of algorithms distributed in different layers. Next, we will zoom in one level deeper on each of the hierarchical steps.

### Pre-processing

The purpose of pre-processing is to assess the quality of the image data and improve the data quality by suppressing or minimizing unwanted disturbances (noise, aliasing, geometric distortions, etc.) before being fed to the following image analysis stage. It takes place at the lowest level (so-called iconic level) of image processing —the input and output of the corresponding mapping function(s) are both intensity images, i.e., $f_{anal}:I \to I^\prime$. Representative image pre-processing algorithms in optical metrology includes but not limited to:

- **Denoising**: In optical metrology, noise in captured raw intensity data has several sources that are related to the electronic noise of photodetectors and the coherent noise (so-called speckle). Typical numerical approaches to noise reduction include median filter^109^, spin filter^110^, anisotropic diffusion^111^, coherence diffusion^112^, Wavelet^113^, windowed Fourier transform (WFT)^114,115^, block matching 3D (BM3D)^116^, etc. For more detailed information and comparisons of these algorithms, the reader may refer to the reviews by Kulkarnia and Rastogi^117^ and Bianco et al.^118^.
- **Enhancement**: Image enhancement is a crucial pre-processing step in intensity-based fringe analysis approaches, such as fringe tracking or skeletonizing. Referring to the intensity model, the fringe pattern may still be disturbed by locally varying background and intensity modulation after denoising. Several algorithms have been developed for fringe pattern enhancement, e.g., adaptive filter^119^, bidimensional empirical mode decomposition^120,121^, and dual-tree complex wavelet transform^122^.
- **Color channel separation**: Because a Bayer color sensor-camera captures three monochromatic (red, green, and blue) images at once, color multiplexing techniques are often employed in optical metrology to speed up the image acquisition process^123–127^. However, the separation of three color channels is not so straightforward due to the coupling and imbalance among the three color channels. Many cross-talk-matrix-based color channel calibration and leakage correction algorithms have been proposed to minimize such side effects^128–130^.
- **Image registration and rectification**: Image registration and rectification are aimed at aligning two or more images of the same object to a reference or correcting image distortion due to lens aberration. In stereophotogrammetry, epipolar (stereo) rectification determines a reprojection of each image plane so that pairs of conjugate epipolar lines in both images become collinear and parallel to one of the image axes^108^.
- **Interpolation**: Image interpolation algorithms, such as the nearest neighbor, bilinear, bicubic^109^, and nonlinear regression^131^ are necessary when the measured intensity image is sampled at an insufficient dense grid. In DIC, to reconstruct displacements with subpixel accuracy, the correlation criterion must be evaluated at non-integer-pixel locations^132–134^. Therefore, image interpolation is also a key algorithm for DIC to infer subpixel gray values and gray-value gradients in many subpixel displacement registration algorithms, e.g., the Newton–Raphson method^133–135^.
- **Extrapolation**: Image extrapolation, especially fringe extrapolation is often employed in Fourier transform (FT) fringe analysis methods to minimize the boundary artifacts induced by spectrum leakage. Schemes for the extrapolation of the fringe pattern beyond the borders have been reported, such as soft-edged frequency filter^136^ and iterative FT^137^.

### Analysis

Image analysis is the core component of the image-processing architecture to extract the key information-bearing parameter(s) reflecting the desired physical quantity being measured from the input images. In phase measurement techniques, image analysis refers to the reconstruction of phase information from the fringe-like modulated intensity distribution(s), i.e., $f_{anal}:I \to \phi$.

- **Phase demodulation**: The aim of phase demodulation, or more specifically, fringe analysis, is to obtain the wrapped phase map from the quasi-periodic fringe patterns. Various techniques for fringe analysis have been developed to meet different requirements in diverse applications, which can be broadly classified into two categories:
  - **Spatial phase demodulation**: Spatial phase-demodulation methods are capable of estimating the phase distribution through a single-fringe pattern. FT^138,139^, WFT^114,115,140^, and wavelet transform (WT)^141^ are classical methods for the spatial carrier fringe analysis. For closed-fringe patterns without the carrier, alternative methods, such as Hilbert spiral transform^142,143^, regularized phase tracking (RPT)^144,145^ and frequency-guided sequential demodulation^146,147^, can be applied provided that the cosinusoidal component of the fringe pattern can be extracted by pre-processing algorithms of denoising, background removal, and fringe normalization. The interested reader may refer to the book by Servin et al.^148^ for further details.
  - **Temporal phase demodulation**: Temporal phase-demodulation techniques detect the phase distribution from the temporal variation of fringe signals, as typified by heterodyne interferometry^149^ and phase-shifting techniques^150^. Many phase-shifting algorithms have originally been proposed for optical interferometry/holography and later been adapted and extended to fringe projection, for example, standard N-step phase-shifting algorithm^151^, Hariharan 5-step algorithm^21^, 2 + 1 algorithm^152^ etc. The interested reader may refer to the chapter “Phase shifting interferometry”^153^ of the book edited by Malacara^4^ and the review article by Zuo et al.^32^ for more details about phase-shifting techniques in the contexts of optical interferometry and FPP, respectively.
- **Phase unwrapping**: No matter which phase-demodulation technique is used, the retrieved phase distribution is mathematically wrapped to the principal value of the arctangent function ranging between −*π* and *π*. The result is what is known as a wrapped phase image, and phase unwrapping has to be performed to remove any 2*π*-phase discontinuities. Phase unwrapping algorithms can be broadly classified into three categories:
  - **Spatial phase unwrapping**: Spatial phase unwrapping methods use only a single wrapped phase map to retrieve the corresponding unwrapped phase distribution, and the unwrapped phase of a given pixel is derived based on the adjacent phase values. Representative methods include Goldstein’s method^154^, reliability-guided method^155^, Flynn’s method^156^, minimal Lp-norm method^157^, and phase unwrapping max-flow/min-cut (PUMA) method^158^. The interested reader may refer to the book by Ghiglia et al. for more technical details. There are also many reviews on the performance comparisons of different unwrapping algorithms for specific applications^159–161^. Limited by the assumption of phase continuity, spatial phase unwrapping methods cannot fundamentally address the inherent fringe order ambiguity problem when the phase difference between neighboring pixels is greater than *π*.
  - **Temporal phase unwrapping**: To remove the phase ambiguity, temporal phase unwrapping methods generally generate different or synthetic wavelengths by adjusting flexible system parameters (wavelength, angular separation of light sources, spatial frequency, orientation of the projected fringe patterns) step by step, so that the object can be covered by fringes with different periods. Representative temporal phase unwrapping algorithms include gray-code methods^162,163^, multi-frequency (hierarchical) methods^164–166^, multi-wavelength (heterodyne) methods^167–169^, and number-theoretical methods^170–173^. For more detailed information about these methods, the reader can refer to the comparative review by Zuo et al.^174^ The advantage of temporal phase unwrapping lies in that the unwrapping is neighborhood-independent and proceeds along the time axis on the pixel itself, enabling an absolute evaluation of the mod-2*π* phase distribution.
  - **Geometric phase unwrapping**: Geometric phase unwrapping approaches can solve the phase ambiguity problem by exploiting the epipolar geometry of projector–camera systems. If the measurement volume can be predefined, depth constraints can be incorporated to preclude some phase ambiguities corresponding to the candidates falling out of the measurement range^175–185^. Alternatively, an adaptive depth-constraint strategy can provide pixel-wise depth constraint ranges according to the shape of the measured object^186^. By introducing more cameras, tighter geometry constraints can be enforced so as to guarantee the unique correspondence and improve the unwrapping reliability^185,187^.

In stereomatching techniques, image analysis refers to determining (tracking or matching) the displacement vector of each pixel point between a pair of acquired images, i.e., $f_{anal}:(I_r,I_d) \to (D_x,D_y)$. In the routine implementation for DIC and stereophotogrammetry, a region of interest (ROI) or subset in the image is specified at first. The subset is further divided into an evenly spaced virtual grid. The similarity is evaluated at each point of the virtual grid in the reference image to obtain the displacement between two subsets. A full-field displacement map can be obtained by sliding the subset in the searching area of the reference image and obtaining the displacement at each location.

- **Subset correlation**: In DIC, to quantitatively evaluate the similarity or difference between the selected reference subset and the target subset, several correlation criteria have been proposed, such as cross-correlation (CC), the sum of absolute difference (SAD), the sum of squared difference (SSD), zero-mean normalized cross-correlation criterion (ZNCC), zero-mean normalized sum of squared difference (ZNSSD), and the parametric sum of squared difference (PSSD)^188–190^. The subsequent matching procedure is realized by identifying the peak (or valley) position of the correlation coefficient distribution based on certain optimization algorithms. In stereophotogrammetry, nonparametric costs rely on the local ordering (i.e., Rank^191^, Census^192^, and Ordinal measures^193^) of intensity values, which are more frequently used due to their robustness against radiometric changes and outliers, especially near object boundaries^192–194^.
- **Subpixel refinement**: The subset correlation methods mentioned above can only provide integer-pixel displacements. To further improve the measurement resolution and accuracy, many subpixel refinement methods were developed, including intensity interpolation (i.e., the coarse–fine search method)^195,196^, correlation coefficient curve-fitting^133,197^, gradient-based method^198,199^, Newton–Raphson (NR) algorithm^135,200,201^, and inverse compositional Gauss–Newton (IC-GN) algorithm^202–204^. Among these algorithms, NR and IC-GN are most commonly used for their high registration accuracy and effectiveness in handling high-order surface transformations. However, they suffer from expensive computation cost stemming from their iterative nonlinear optimization and repeated subpixel interpolation. Therefore, accurate initial guesses obtained by integer-pixel subset correlation methods are critical to ensure the rapid convergence^205^ and reduce the computational cost^206^. In stereovision, the matching algorithms can be classified as local^207–209^, semi-global^210^, and global methods^211^. Local matching methods utilize the intensity information of a local subset centered at the pixel to be matched. Global matching methods take the result obtained by local matching methods as the initial value and then optimize the disparity by minimizing a predefined global energy function. Semi-global matching methods reduce the 2D global energy minimization problem into a 1D one, enabling faster and more efficient implementations of stereomatching.

### Postprocessing

In optical metrology, the main task of postprocessing is to further refine the measured phase or retrieved displacement field, and finally transform them into the desired physical quantity of the measured object, i.e., the corresponding operator $f_{post}:\phi {{{\mathrm{/}}}}(D_x,D_y) \to q$, where *q* is the desired sample quantity.

- **Denoising**: Instead of applying to raw fringe patterns, image denoising can also be used as a postprocessing algorithm to remove noise directly from the retrieved phase distribution. Various phase denoising algorithms have been proposed, such as least-square (LS) fitting^212^, anisotropic average filter^213^, WFT^214^, total variation^215^, and nonlocal means filter^216^.
- **Digital refocusing**: The numerical reconstruction of propagating wavefronts by diffraction is a unique feature of digital holography. Since the hologram of the object may not be recorded in the in-focus plane. Numerical diffraction or backpropagation algorithms (e.g., Fresnel diffraction and angular spectrum methods) should be used to obtain a focused image by performing a plane-by-plane refocusing after the image acquisition^217–219^.
- **Error compensation**: There are various types of phase errors associated with optical metrology systems, such as phase-shifting error, intensity nonlinearity, and motion-induced error, which can be compensated with different types of postprocessing algorithms^60,220,221^. In digital holographic microscopy, the microscope objective induces additional phase curvature on the measured wavefront, which needs to be compensated in order to recover the phase information induced by the sample. Typical numerical phase-aberration compensation methods include double exposure^222^, 2D spherical fitting^223^ Zernike polynomials fitting^224^, Fourier spectrum filtering^225^, and principal component analysis (PCA)^226^.
- **Quantity transformation**: The final step of postprocessing and also the whole measurement chain is to convert the phase or displacement field into the desired sample quantity, such as height, thickness, displacement, stress, strains, and 3D coordinates, based on sample parameters (e.g., refractive index, relative stress constant) or calibrated system parameters (e.g., sensitivity vector and camera (intrinsic, extrinsic) parameters). The optical setup should be carefully designed to optimize the sensitivity with respect to the measuring quantity in order to achieve a successful and efficient measurement^227,228^.

Finally, it should be mentioned that since optical metrology is a rapidly expanding field in both its scientific foundations and technological developments, the image-processing hierarchy used here cannot provide full coverage of all relevant methods and technologies. For example, phase retrieval and wave-field sensing technologies have shown great promise for inexpensive, vibration-tolerant, non-interferometric, optical metrology of optical surfaces and systems^66,67^. These methods constitute an important aspect of computational imaging as they often involve solving ill-posed inverse problems. There are also some optical metrology methods based on solving constrained optimization problems with added penalties and relaxations (e.g., RPT phase demodulation^144,145^ and minimal Lp-norm phase unwrapping methods^157^), which may make pre- and postprocessing unnecessary. For a detailed discussion on this topic, please refer to the subsection “Solving inverse optical metrology problems: issues and challenges”.

## Brief introduction to deep learning

Deep learning is a subset of machine learning, which is defined as the use of specific algorithms that enable machines to automatically learn patterns from large amounts of historical data, and then utilize the uncovered patterns to make predictions about the future or enable decision making under uncertain intelligently^229,230^. The key specific algorithm used in machine learning is the ANN, which exploits input data ${{{\mathbf{x}}}} \in {{{\mathcal{X}}}} \subseteq {\Bbb R}^n$ to predict an unknown output ${{{\mathbf{y}}}} \in {{{\mathcal{Y}}}}$. The tasks accomplished by the ANN can be broadly divided as classification tasks or regression tasks, depending on whether **y** is a discrete label or a continuous value. The objective of machine learning is then to find a mapping function $f:{{{\mathbf{x}}}} \to {{{\mathbf{y}}}}$. The choice of such functions is given by the neural network models with additional parameters ${{{\mathbf{\theta }}}} \in \Theta$: i.e., ${{{\hat{\mathbf y}}}} = f\left( {{{{\mathbf{x}}}},{{{\mathbf{\theta }}}}} \right) \approx {{{\mathbf{y}}}}$. The goal of this section is to provide a brief introduction to deep learning, as a preparation for the introduction of its applications in optical metrology later on.

### Artificial neural network (ANN)

**Fig. 4:**
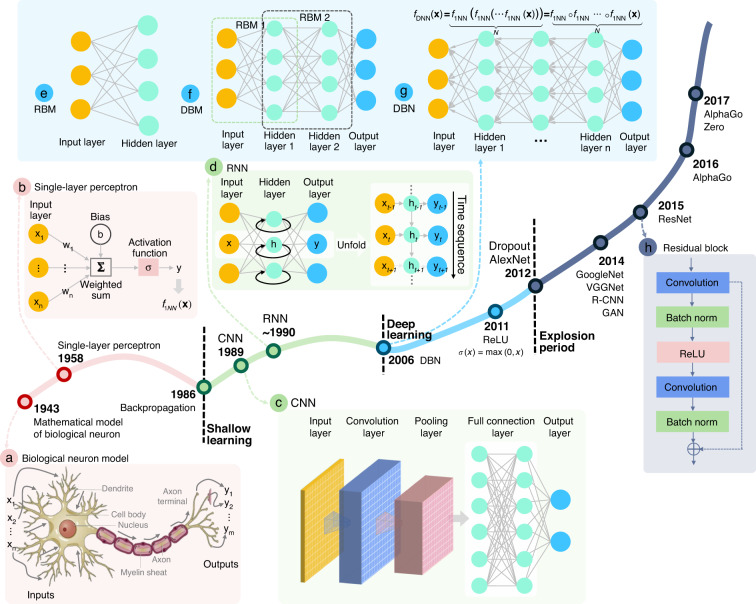
Historical evolution of artificial neural networks and deep learning, where the horizontal axis represents time, and the vertical axis represents research and development activities. **a** Biological neuron model^458^. **b** The single-layer perceptron: an artificial neuron calculates the weighted sum (∑) of the inputs (based on weights *θ*_1_ − *θ*_*n*_), and maps them to the output through an activation function. **c** CNN: convolutional neural network, consists of input layer, convolution layer, pooling layer, full connection layer and output layer. **d** RNN: recurrent neural network, the input of the hidden layer includes not only the output of the input layer but also the output of the hidden layer at the previous moment. **e** RBM: Restricted Boltzmann Machines, an undirected probability graph model with an input layer and a hidden layer. **f** DBM: Deep Boltzmann Machine, consists of several RBM units stacked. The connections between all layers are undirected. **g** DBN: Deep Belief Network, consists of several DBM units stacked. The connection between the right two layers is undirected, while other connections are directed. **h** Residual block: consists of two sets of convolutional layers activated by ReLU stacked one above the other

Inspired by the biological neural network (Fig. 4a), ANNs are composed of interconnected computational units called artificial neurons. As illustrated in Fig. 4b, the simplest neural network following the above concept is the perceptron, which consists of only one single artificial neuron^231^. An artificial neuron takes a bias *b* and weight vector ${{{\mathbf{w}}}} = \left( {w_1,w_2, \cdots ,w_n} \right)^T$ as parameters ${{{\mathbf{\theta }}}} = \left( {b,w_1,w_2, \cdots ,w_n} \right)^T$ to map the input ${{{\mathbf{x}}}} = \left( {x_1,x_2, \cdots ,x_n} \right)^T$ to the output $f_P\left( {{{\mathbf{x}}}} \right)$ through a nonlinear activation function *σ* as

3 $$f_P\left( {{{\mathbf{x}}}} \right) = \sigma \left( {{{{\mathbf{w}}}}^T{{{\mathbf{x}}}} + b} \right)$$

Typical choices for such activation functions are the sign function $\sigma \left( x \right) = sgn\left( x \right)$, sigmoid function $\sigma \left( x \right) = \frac{1}{{1\, + \,e^{ - x}}}$, hyperbolic tangent function $\sigma \left( x \right) = \frac{{e^x\, - \,e^{ - x}}}{{e^x\, + \,e^{ - x}}}$, and rectified linear unit (ReLU) $\sigma \left( x \right) = \max \left( {0,x} \right)$^232^. A single perceptron can only model a linear function, but because of the activation functions and in combination with other neurons, the modeling capabilities will increase dramatically. Arranged in a single layer, it has already been shown that neural networks can approximate any continuous function *f*(**x**) on a compact subset of ${\Bbb R}^n$. A single-layer network, also called single-layered perceptron (SLP), is represented as a linear combination of *M* individual neurons:

4 $$f_{1NN}\left( {{{\mathbf{x}}}} \right) = \mathop {\sum}\limits_{i = 1}^M {v_i} \sigma \left( {{{{\mathbf{w}}}}_i^T{{{\mathbf{x}}}} + b_i} \right)$$

where *v*_*i*_ is the combination weight of the *i*th neuron. We can further extend the mathematical specification of SLP by stacking several single-layer networks into a multi-layered perceptron (MLP)^233^. As the network goes deeper (number of layers increase), the number of free parameters increases, as well as the capability of the network to represent highly nonlinear functions^234^. We can formalize this mathematically by stacking several single-layer networks into a deep neural network (DNN) with *N* layers, i.e.

5 $$f_{DNN}\left( {{{\mathbf{x}}}} \right)\, = \,\underbrace {f_{1NN}\left( {f_{1NN}\left( { \cdots f_{1NN}\left( {{{\mathbf{x}}}} \right)} \right)} \right)}_N\, = \,\underbrace {f_{1NN} \circ f_{1NN} \cdots \circ f_{1NN}\left( {{{\mathbf{x}}}} \right)}_N$$

where the circle ◦ is the symbol for the composition of functions. The first layer is referred to as the input layer, the last as the output layer, and the layers in between the input and output are termed as hidden layers. We refer to these using the term “deep”, when a neural network contains many hidden layers, hence the term “deep learning”.

### Neural network training

Having gained basic insights into neural networks and their basic topology, we still need to discuss how to train the neural network, i.e., how its parameters **θ** are actually determined. In this regard, we need to select the appropriate model topology for the problem to be solved and specify the various parameters associated with the model (known as “hyper-parameters”). In addition, we need to define a function that assesses the quality of the network parameter set **θ**, the so-called loss function *L*, which quantifies the error between the predicted value ${{{\hat{\mathbf y}}}} = f_{{{\mathbf{\theta }}}}\left( {{{\mathbf{x}}}} \right)$ and the true observation **y** (label)^235^.

Depending on the type of task accomplished by the network, the loss function can be divided into classification loss and regression loss. Commonly used classification loss functions include hinge loss ($L_{Hinge} = \mathop {\sum}\nolimits_{i = 1}^n {\max [0,1 - {{{\mathrm{sgn}}}}(y_i)\hat y_i]}$) and cross-entropy loss $L_{CE} = - \mathop {\sum}\nolimits_{i = 1}^n {[y_i\log \hat y_i + (1 - y_i)\log (1 - \hat y_i)]}$)^236^. Since the optical metrology tasks involved in this review mainly belong to regression tasks, here we focus on the regression loss functions. The mean absolute error (MAE) loss ($L_{MAE} = \frac{1}{n}\mathop {\sum}\nolimits_{i = 1}^n {\left| {y_i - \hat y_i} \right|}$) and the mean squared error (MSE) loss ($L_{MSE} = \frac{1}{n}\mathop {\sum}\nolimits_{i = 1}^n {(y_i - \hat y_i)^2}$) are the two most commonly used loss functions, which are also known as *L*1 loss and *L*2 loss, respectively. In image-processing tasks, MSE is usually converted into a peak signal-to-noise ratio (PSNR) metric: $L_{PSNR} = 10\,{{{\mathrm{log}}}}_{10}\frac{{MAX^2}}{{L_{MSE}}}$, where *MAX* is the maximum pixel intensity value within the dynamic range of the raw image^237^. Other variants of *L*1 and *L*2 loss include RMSE, Euclidean loss, smooth *L*1, etc.^238^. For natural images, the structural similarity (SSIM) index is a representative image fidelity measurement, which judges the structural similarity of two images based on three metrics (luminance, contrast, and structure): $L_{SSIM} = l({{{\mathbf{y}}}},\widehat {{{\mathbf{y}}}})c({{{\mathbf{y}}}},\widehat {{{\mathbf{y}}}})s({{{\mathbf{y}}}},\widehat {{{\mathbf{y}}}})$^239^, where $l({{{\mathbf{y}}}},\widehat {{{\mathbf{y}}}})$, $c({{{\mathbf{y}}}},\widehat {{{\mathbf{y}}}})$, and $s({{{\mathbf{y}}}},\widehat {{{\mathbf{y}}}})$ are the similarities of the local patch luminances, contrasts, and structures, respectively. For more details about these loss functions, readers may refer to the article by Wang and Bovik^240^. With the defined loss function, the objective behind the training process of ANNs can be formalized as an optimization problem^241^

6 $${\widehat{\mathbf{\theta}}} = {\mathop{{\arg}\,{\min}}\limits_{{\mathbf{\theta}}\in \Theta}}{L}(f_{\theta}({\bf{x}},{\bf{y}}))$$

The learning schemes can be broadly classified into three categories, supervised learning, semi-supervised learning, and unsupervised learning^36,242–244^. Supervised learning dominates the majority of practical applications, in which a neural network model is optimized based on a large amount dataset of labeled data pairs (**x**, **y**), and the training process amounts to find the model parameters $\widehat {{{\mathbf{\theta }}}}$ that best predict the data based on the loss function $L\left( {\widehat {{{\mathbf{y}}}},{{{\mathbf{y}}}}} \right)$. In unsupervised learning, training algorithms process input data **x** without corresponding labels **y**, and the underlying structure or distribution in the data has to be modeled based on the input itself. Semi-supervised learning sits in between both supervised and unsupervised learning, where a large amount of input data **x** is available and only some of the data is labeled. More detailed discussions about semi-supervised and unsupervised learning can be found in the “Future directions” section.

### From perceptron to deep learning

As summarized in Fig. 4, despite the overall upward trend, a broader look at the history of deep learning reveals three major waves of development. Concepts of machine learning and deep learning commenced with the research into the artificial neural network, which was originated from the simplified mathematical model of biological neurons established by McCulloch and Pitts in 1943^245^. In 1958, Rosenblatt^231^ proposed the idea of perceptron, which was the first ANN that allows neurons to learn. The emergence of perceptron marked the first peak of neural network development. However, a single-layer perceptron model can only solve linear classification problems and cannot solve simple XOR and XNOR problems^246^. These limitations caused a major dip in their popularity and stagnated the development of neural networks for nearly two decades.

In 1986, Rumelhart et al.^247^ proposed the idea of a backpropagation algorithm (BP) for MLP, which constantly updates the network parameters to minimize the network loss based on a chain rule method. It effectively solves the problems of nonlinear classification and learning, leading neural networks into a second development phase of “shallow learning” and promoting a boom of shallow learning. Inspired by the mammalian visual cortex (stimulated in the restricted visual field)^248^, LeCun et al.^249^ proposed the biologically inspired CNN model based on the BP algorithm in 1989, establishing the foundation of deep learning for modern computer vision. During this wave of development, various models like long short-term memory (LSTM) recurrent neural network (RNN), distributed representation, and processing were developed and continue to remain key components of various advanced applications of deep learning to this date. Adding more hidden layers to the network allows a deep architecture to be built, which can accomplish more complex mappings. However, training such a deep network is not trivial because once the errors are back-propagated to the first few layers, they become negligible (so-called gradient vanishing), making the learning process very slow or even fails^250^. Moreover, the limited computational capacity of the available hardware at that time could not support training large-scale neural networks. As a result, deep learning suffered a second major roadblock.

In 2006, Hinton et al.^251,252^ proposed a Deep Belief Network (DBN) (the composition of simple, unsupervised networks such as Deep Boltzmann Machines (DBMs)^253^ (Fig. 4f) or Restricted Boltzmann Machines (RBMs)^254^ (Fig. 4e)) training approach based on the brain graphical models, trying to overcome the gradient-vanishing problem. They gave the new name “deep learning” to multilayer neural network-related learning methods^251,252^. This milestone revolutionized the approaching prospects in machine learning, leading neural networks into the third upsurge along with the development of computer hardware performance, the development of GPU acceleration technology, and the availability of massive labeled datasets.

In 2012, Krizhevsky et al.^255^ proposed a deep CNN architecture — AlexNet, which won the 2012 ImageNet competition, making CNN^249,256^ become the dominant framework for deep learning after more than 20 years of silence. Meanwhile, several new deep-learning network architectures and training approaches (e.g., ReLU^232^ given by $\sigma (x) = \max (0,x)$, and Dropout^257^ that discards a small but random portion of the neurons during each iteration of training to prevent neurons from co-adapting to the same features) were developed to further combat the gradient vanishing and ensure faster convergence. These factors have led to the explosive growth of deep learning and its applications in image analysis and computer vision-related problems. Different from CNN, RNN is another popular type of DNN inspired by the brain’s recurrent feedback system. It provides the network with additional “memory” capabilities for previous data, where the inputs of the hidden layer consist of not only the current input but also the output from the previous step, making it a framework specialized in processing sequential data^258–260^ (Fig. 4d). CNNs and RNNs usually operate on Euclidean data like images, videos, texts, etc. With the diversification of data, some non-Euclidean graph-structured data, such as 3D-point clouds and biological networks, are also considered to be processed by deep learning. Graph neural networks (GNNs), where each node aggregates feature vectors of its neighbors to compute its new feature vector (a recursive neighborhood aggregation scheme), are effective graph representation learning frameworks specifically for non-Euclidean data^261,262^.

With the focus of more attention and efforts from both academia and industry, different types of deep neural networks have been continuously proposed in recent years with exponential growth, such as VGGNet^263^ (VGG means “Visual Geometry Group”), GoogLeNet^264^ (using “GoogLe” instead of “Google” is a tribute to LeNet, one of the earliest CNNs developed by LeCun^256^), R-CNN (regions with CNN features)^265^, generative adversarial network (GAN)^266^, etc. In 2015, the emergence of the residual block (Fig. 4h), containing two convolutional layers activated by ReLU that allow the information (from the input or those learned in earlier layers) to penetrate more into the deeper layers, significantly reduces the vanishing gradient problem as the network gets deeper, making it possible to train large-scale CNNs efficiently^267^. In 2016, the Google-owned AI company DeepMind shocked the world by beating Lee Se-dol with its AlphaGo AI system, alerting the world to deep learning, a new breed of machine learning that promised to be smarter and more creative than before^268^. For a more detailed description of the history and development of deep learning, readers can refer to the chronological review article by Schmidhuber^39^.

### Convolutional neural network (CNN)

In the subsection “Artificial neural network”, we talked about the simplest DNN, so-called MLPs, which basically consist of multiple layers of neurons, each fully connected to those in the adjacent layers. Each neuron receives some inputs, which are multiplied by their weights, with nonlinearity applied via activation functions. In this subsection, we will talk about CNNs, which are considered an evolution of the MLP architecture that is developed to process data in single or multiple arrays, and thus are more appropriate to handle image-like input. Given the prevalence of CNNs in image processing and analysis tasks, here we briefly review some basic ideas and concepts widely used in CNNs. For a comprehensive introduction to CNN, we refer readers to the excellent book by Goodfellow et al.^36^.

**Fig. 5:**
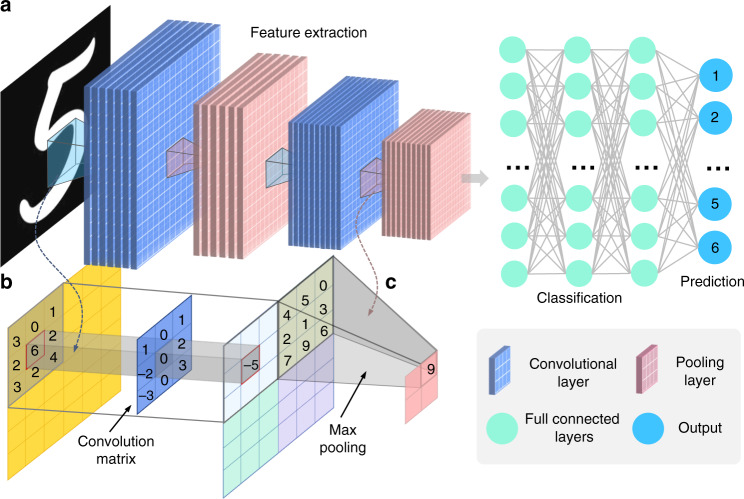
The typical CNN architecture for image-classification tasks. **a** The typical CNN architecture for image classification tasks consists of the input layer, convolutional layers, fully connected layers, and output prediction. **b** Convolution operation. **c** Pooling operation

CNN follows the same pattern as MLP: artificial neurons are stacked in hidden layers on top of each other; parameters are learned during network training with nonlinearity applied via activation functions; the loss $L\left( {\widehat {{{\mathbf{y}}}},{{{\mathbf{y}}}}} \right)$ is calculated and back-propagated to update the network parameters. The major difference between them is that instead of regular fully connected layers, CNN uses specialized convolution layers to model locality and abstraction (Fig. 5b). At each layer, the input image ${{{\mathbf{x}}}}$ (lexicographically ordered) is convolved with a set of convolutional filters **W** (note here **W** represents block-Toeplitz convolution matrix) and added biases **b** to generate a new image, which is subjected to an elementwise nonlinear activation function *σ* (normally use ReLU function $\sigma (x) = \max (0,x)$), and the same structure is repeated for each convolution layer *k*:

7 $${{{\mathbf{x}}}}_{}^k = \sigma \left( {{{{\mathbf{W}}}}_{}^{k - 1}{{{\mathbf{x}}}}_{}^{k - 1} + {{{\mathbf{b}}}}_{}^{k - 1}} \right)$$

The second key difference between CNNs and MLPs is the typical incorporation of pooling layers in CNNs, where pixel values of neighborhoods are aggregated by applying a permutation invariant function, such as the max or mean operation, to reduce the dimensionality of the convolutional layers and allows significant features to propagate downstream without being affected by neighboring pixels (Fig. 5c). The major advantage of such an architecture is that CNNs exploit spatial dependencies in the image and only consider a local neighborhood for each neuron, i.e., the network parameters are shared in such a way that the network performs convolution operations on images. In other words, the idea of a CNN is to take advantage of a pyramid structure to first identify features at the lowest level before passing these features to the next layer, which, in turn, create features of a higher level. Since the local statistics of images are invariant to location, the model does not need to learn weights for the same feature occurring at different positions in an image, making the network equivariant with respect to translations of the input. It makes CNNs especially suitable for processing images captured in optical metrology, e.g., a fringe pattern consisting of sinusoidal signal repeated over different image locations. In addition, it also drastically reduces the number of parameters (i.e., the number of weights no longer depends on the size of the input image) that need to be learned.

Figure 5a shows a CNN architecture for the image-classification task. Every layer of a CNN transforms the input volume to an output volume of neuron activation, eventually leading to the final fully connected layers, resulting in a mapping of the input data to a 1D feature vector. A typical CNN configuration consists of a sequence of convolution and pooling layers. After passing through a few pairs of convolutional and pooling layers, all the features of the image have been extracted and arranged into a long tube. At the end of the convolutional stream of the network, several fully connected layers (i.e., regular neural network architecture, MLP, that discussed in the previous subsection) are usually added to fatten the features into a vector, with which tasks, such as classifications, can be performed. Starting with LeNet^256^, developed in 1998 for recognizing handwritten characters with two convolutional layers, CNN architectures have evolved since then to deeper CNNs like AlexNet^264^ (5 convolutional Layers) and VGGNet^263^ (19 convolutional Layers) and beyond to more advanced and super-deep networks like GoogLeNet^264^ and ResNet^267^, respectively. These CNNs have been extremely successful in computer vision applications, such as object detection^269^, action recognition^270^, motion tracking^271^, and pose estimation^272^.

### Fully convolutional network architectures for image processing

Conventionally, CNNs have been used for solving classification problems. Due to the presence of a parameter-rich fully connected layer at the end of the network, typical CNNs throw away spatial information and produce non-spatial outputs. However, for most image-processing tasks that we encountered earlier in the Section “Image processing in optical metrology”, the network must have a whole-resolution output with the same or even larger size compared with the input, which is commonly referred to as dense prediction (contrary to the single target category per image)^273^. Specifically, fully convolutional network architectures without fully connected layers should be used for this purpose, which accepts input of any size, is trained with a regression loss, and produces an output of the corresponding dimensions^273,274^. Here, we briefly review three representative network architectures with such features.

**Fig. 6:**
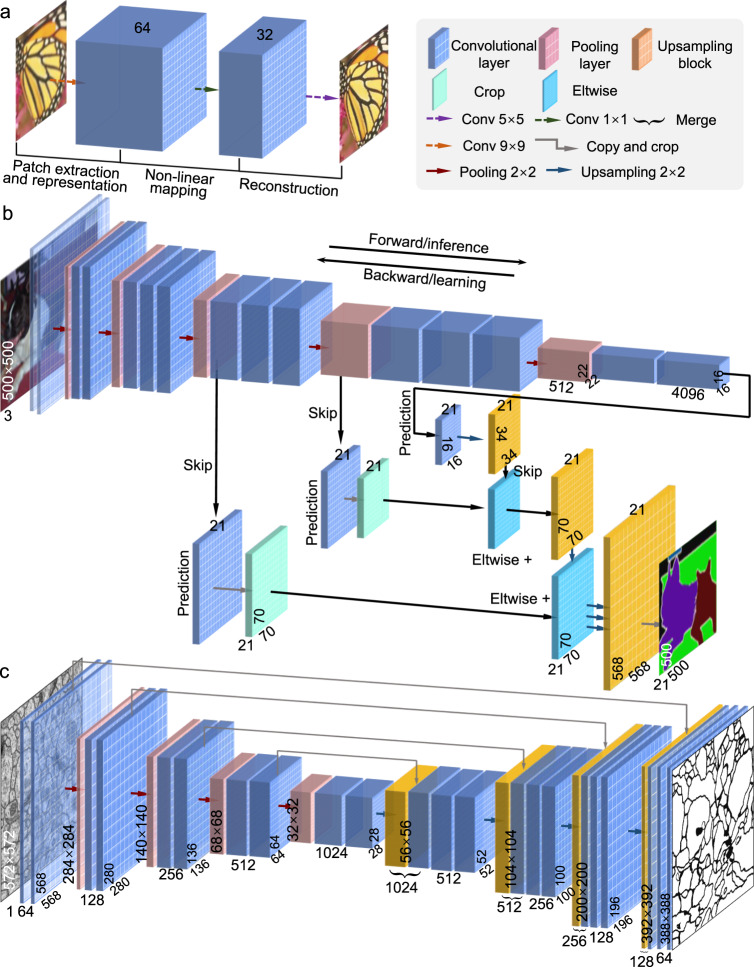
Three typical CNN structures for image-processing tasks with pixel-level image output. **a** SRCNN. **b** FCN. **c** U-Net
- **FCN**: The fully convolutional network (FCN) proposed by Long et al.^273^ is a popular strategy and baseline for semantic-segmentation tasks. FCN is inspired by the fact that the fully connected layers in classification CNN (Fig. 5) can also be viewed as convolutions with kernels that cover their entire input regions. As illustrated in Fig. 6b, FCN uses the existing classification CNN as the encoder module of the network and replace these fully connected layers into 1 × 1 convolution layers (also termed as deconvolution layers) as the decoding module, enabling the CNN to upsample the input feature maps and get pixel-wise output. In FCN, skip connections combining (simply adding) information in fine layers and coarse layers enhances the localization capability of the network, allowing for the reconstruction of accurate fine details that respect global structure. FCN and its variants have achieved great success in the application of dense pixel prediction as required in many advanced computer vision understanding tasks^280^.
- **U-Net**: Ronneberger et al.^281^ took the idea of FCN one step further and proposed the U-Net architecture, which replaces the one-step upsampling part with a bunch of complimentary upsampling convolutions layers, resulting in a quasi-symmetrical encoder-decoder model architecture. As illustrated in Fig. 6c, the basic structure of U-Net consists of a contractive branch and an expansive branch, which enables multiresolution analysis and general multiscale image-to-image transforms. The contractive branch (encoder) downsamples the image using conventional strided convolution, producing a compressed feature representation of the input image. The expansive branch (decoder), complimentary to the contractive branch, uses upsampling methods like transpose convolution to provide the processed result with the same size as the input. In addition, U-Net features skip connections that concatenate the matching resolution levels of the contractive branch and the expansive branch. Ronneberger’s U-Net is a breakthrough toward automatic image segmentation and has been successfully applied in many tasks that require image-to-image transforms^282^.

- **SRCNN**: In conventional CNN, the downsampling effect of pooling layers results in an output with a far lower resolution than the input. Thus, a relatively naive and straightforward solution is simply stacking several convolutions layers while skipping pooling layers to preserve the input dimensions. Dong et al.^275^ firstly adopt this idea and propose SRCNN for the image super-resolution task. SRCNN utilizes traditional upsampling algorithms to obtain low-resolution images and then refine them by learning an end-to-end mapping from interpolated coarse images to high-resolution images of the same dimension but with more details, as illustrated in Fig. 6a. Due to its simple ideal and implementation, SRCNN has gradually become one of the most popular frameworks in image super-resolution^276^ and been extended to many other tasks such as radar image enhancing^277^, underwater image high definition display^278^, and computed tomography^279^. One major disadvantage of SRCNN is the cost of time and space to keep the whole resolution through the whole network, limiting SRCNN only practical for relatively shallow network structures.

Since the feature extraction is only performed in low-dimensional space, the computation and spatial complexity of the above encoder-decoder structured networks (FCN and U-Net) can be much reduced. Therefore, the encoder-decoder CNN structure has become the mainstream for image segmentation and reconstruction^283^. The encoder is usually a classic CNN (Alexnet, VGG, Resnet, etc.) in which downsampling (pooling layers) is adopted to reduce the input dimension so as to generate low-resolution feature maps. The decoder tries to mirror the encoder to upsample these feature representations and restore the original size of the image. Thus, how to perform upsampling is of great importance. Although traditional upsampling methods, e.g., nearest neighbor, bilinear, and bicubic interpolations, are easy to implement, deep-learning-based upsampling methods, e.g., unpooling^284^, transpose convolution^273^, subpixel convolution^285^, has gradually become a trend. All these approaches can be combined with the model mentioned above to prevent the decrease in resolution and obtain a full-resolution image output.

**Fig. 7:**
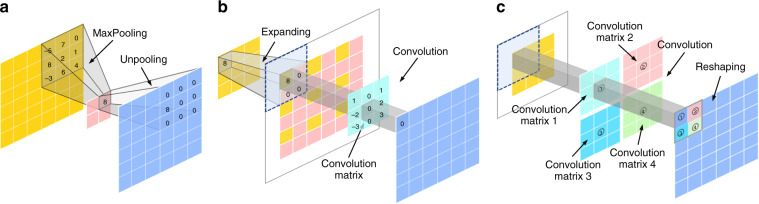
Three deep-learning-based upsampling methods typically used in CNN. **a** Unpooling. **b** Transposed convolution. **c** Sub pixel convolution

- **Unpooling upsampling**: Unpooling upsampling reverts maxpooling by remembering the location of the maxima in the maxpooling layers and in the unpooling layers copy the value to exactly this location, as shown in Fig. 7a.
- **Transposed convolution**: The opposite of the convolutional layers are the transposed convolution layers (also misinterpreted as deconvolution layers^280^), i.e., predicting the possible input based on feature maps sized like convolution output. Specifically, it increases the image resolution by expanding the image by inserting zeros and performing convolution, as shown in Fig. 7b.
- **Sub pixel convolution**: The subpixel layer performs upsampling by generating a plurality of channels by convolution and then reshaping them, as Fig. 7c shows. Within this layer, a convolution is firstly applied for producing outputs with M times channels, where M is the scaling factor. After that, the reshaping operation (*a.k.a*. shuffle) is performed to produce outputs with size *M* times larger than the original.

As discussed in the Section “Image processing in optical metrology”, despite their diversity, the image-processing algorithms used in optical metrology share a common characteristic—they can be regarded as a mapping operator that transforms the content of arbitrary-sized inputs into pixel-level outputs, which fits exactly with DNNs with a fully convolutional architecture. In principle, any fully convolutional network architectures presented here can be used for a similar purpose. By applying different types of training datasets, they can be trained for accomplishing different types of image-processing tasks that we encountered in optical metrology. This provides an alternative approach to process images such that the produced results resemble or even outperform conventional image-processing operators or their combinations. There are also many other potential desirable factors for such a substitution, e.g., accuracy, speed, generality, and simplicity. All these factors were crucial to enable the fast rise of deep learning in the field of optical metrology.

## Invoking deep learning in optical metrology: principles and advantages

Let us return to optical metrology. It is essential that the image formation is properly understood in order to reconstruct the required geometrical or mechanical quantities of the sample, as we discussed in Section “Image formation in optical metrology”. In general, the relation between the observed images ${{{\mathbf{I}}}} \in {\Bbb R}^m$ (frame-stacked lexicographically ordered with *m* × 1 in dimension) and the desired sample parameter (or information-bearing parameter that clearly reflects the desired sample quantity, e.g., phase or displacement field) ${{{\mathbf{P}}}} \in {\Bbb R}^m$ (or ${\Bbb C}^n$) can be described as

8 $${{{\mathbf{I}}}} = {{{\mathcal{N}}}}\left\{ {{{{\mathcal{A}}}}\left( {{{\mathbf{p}}}} \right)} \right\}$$

where ${{{\mathcal{A}}}}$ is the (possibly nonlinear) forward measurement operator mapping from the parameter space to the image space, which is given by the physics laws governing the formation of data; ${{{\mathcal{N}}}}$ represents the effect of noise (not necessarily additive). This model seems general enough to cover almost all image formation processes in optical metrology. However, this does not mean that **p** can be directly obtained from **I**. More specifically, we have to conclude in general from the effect (i.e., the intensity at the pixel) to its cause (i.e., shape, displacement, deformation, or stress of the surface), suggesting that an inverse problem has to be solved.

### Solving inverse optical metrology problems: issues and challenges

**Fig. 8:**
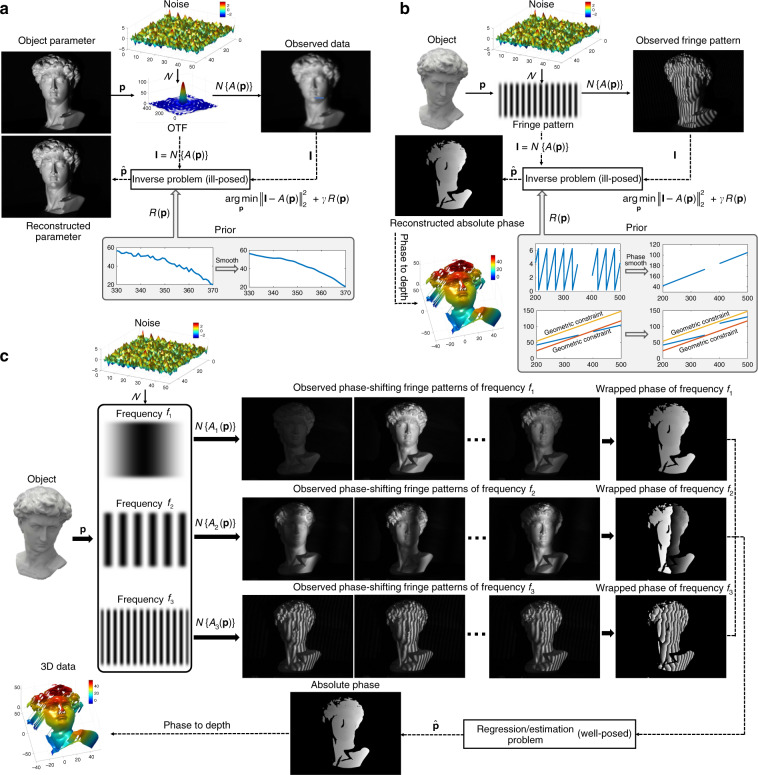
Inverse problems in computer vision and optical metrology. **a** In computer vision, such as image deblurring, the resulting inverse problem is ill-posed since the forward measurement operator ${{{\mathcal{A}}}}$ mapping from the parameter space to the image space is usually poorly conditioned. The classical approach is to impose certain prior assumptions (smoothing) about the solution **p** that helps in regularizing its retrieval. **b** In optical metrology, absolute phase demodulation from a single-fringe pattern exhibits all undesired difficulties of an inverse problem: ill-posedness and ambiguity, which can also be formed as a regularized optimization problem with proper prior assumptions (phase smoothness, geometric constraints) imposed. **c** Optical metrology uses an “active” approach to transform the ill-posed inverse problem into a well-posed estimation or regression problem: by acquiring additional phase-shifted patterns of different frequencies, absolute phase can be easily determined by multi-frequency phase-shifting and temporal phase unwrapping methods

Given the forward model represented by Eq. (8), our task is to find the parameters by an approximate inverse of ${{{\mathcal{A}}}}$ (denoted as $\tilde {{{\mathcal{A}}}}^{ - 1}$) such that $\widehat {{{\mathbf{p}}}} = \widehat {{{\mathcal{R}}}}\left( {{{\mathbf{I}}}} \right) = \tilde {{{\mathcal{A}}}}^{ - 1}\left( {{{\mathbf{I}}}} \right) \approx {{{\mathbf{p}}}}$. However, in real practice, there are many problems involved in this process:

- **Unknown or mismatched forward model**. The success of conventional optical metrology approaches relies heavily on the precise pre-knowledge about the forward model ${{{\mathcal{A}}}}$, so they are often regarded as model-driven or knowledge-driven approaches. In practical applications, the forward model ${{{\mathcal{A}}}}$ used is always an approximate description of reality, and extending it might be challenging due to a limited understanding of experimental perturbations (noise, aberrations, vibration, motion, nonlinearity, saturation, and temperature variations) and non-cooperative surfaces (shiny, translucent, coated, shielded, highly absorbent, and strong scattering). These problems are either difficult to model or result in a too complicated (even intractable) model with a large number of parameters.
- **Error accumulation and suboptimal solution**. As described in the section “Image processing in optical metrology”, “divide-and-conquer” is a common practice for solving complex problems with a sequence of cascaded image-processing algorithms to obtain the desired object parameter. For example, in FPP, the entire image-processing pipeline is generally divided into several sub-steps, i.e., image pre-processing, phase demodulation, phase unwrapping, and phase-to-height conversion. Although each sub-problem or sub-step becomes simpler and easier to handle, the disadvantages are also apparent: error accumulation and suboptimal solution, i.e., the aggregation of optimum solutions to subproblems may not be equivalent to the global optimum solution.
- **Ill-posedness of the inverse problem**. In many computer vision and computational imaging tasks, such as image deblurring^24^, sparse computed tomography^25^, and imaging through scattering media^27^, the difficulty in retrieving the desired information **p** from the observation **I** arises from the fact that the operator ${{{\mathcal{A}}}}$ is usually poorly conditioned, and the resulting inverse problem is ill-posed, as illustrated in Fig. 8a. Due to the similar indirect measurement principle, there are also many important inverse problems in optical metrology that are ill-posed, among which the phase demodulation from a single-fringe pattern and phase unwrapping from single wrapped phase distributions are the best known for specialists in optical metrology (Fig. 8b). The simplified model for the intensity distribution of fringe patterns (Eq. (1)) suggests that the observed intensity **I** results from the integration of several unknown components: the average intensity *A*(*x*, *y*), the intensity modulation *B*(*x*, *y*), and the desired phase function *ϕ*(*x*, *y*). Simply put, we do not have enough information to solve the corresponding inverse problem uniquely and stably.

In the fields of computer vision and computational imaging, the classical approach in solving an ill-posed inverse problem is to reformulate the ill-posed original problem into a well-posed optimization problem by imposing certain prior assumptions about the solution **p** that helps in regularizing its retrieval:

9 $$\widehat {{{\mathbf{p}}}}\mathop {{{{{\mathrm{ = }}}}\arg \min }}\limits_{{{\mathbf{p}}}} \left\| {{{{\mathbf{I}}}} - {{{\mathcal{A}}}}\left( {{{\mathbf{p}}}} \right)} \right\|_2^2 + \gamma R\left( {{{\mathbf{p}}}} \right)$$

where || ||_2_ indicates the Euclidean norm, *R*(**p**) is a regularization penalty function that incorporates the prior information about **p**, such as smoothness^286^, sparsity in some basis^287^ or dictionary^288^. *γ* is a real positive parameter (regularization parameter) that governs the weight given to the regularization against the need to fit the measurement and should be selected carefully to make an admissible compromise between the prior knowledge and data fidelity. Such an optimization problem can be solved efficiently with a variety of algorithms^289,290^ and provide theoretical guarantees on the recoverability and stability of the approximate solution to an inverse problem^291^.

Instead of regularizing the numerical solution, in optical metrology, we prefer to reformulate the original ill-posed problem into a well-posed and adequately stable one by actively controlling the image acquisition process so as to add systematically more knowledge about the object to be investigated into the evaluation process^31^. Due to the fact that the optical measurements are frequently carried out in a highly controlled environment, such a solution is often more practical and effective. As illustrated by Fig. 8c, by acquiring additional multi-frequency phase-shifted patterns, absolute phase retrieval becomes a well-posed estimation or regression problem, and the simple standard (unconstrainted, regularization-free) least-square methods in regression analysis provides a stable, precise, and efficient solution^292,293^:

10 $$\widehat {{{\mathbf{p}}}}\mathop {{{{{\mathrm{ = }}}}\arg \min }}\limits_{{{\mathbf{p}}}} \left\| {{{{\mathbf{I}}}} - {{{\mathcal{A}}}}\left( {{{\mathbf{p}}}} \right)} \right\|_2^2$$

The situation may become very different when we step out of the laboratory and into the complicated environment of the real world^294^. The active strategies mentioned above often impose stringent requirements on the measurement conditions and the object under test. For instance, high-sensitivity interferometric measurement in general needs a laboratory environment where the thermal-mechanical settings are carefully controlled to preserve beam path conditions and minimize external disturbances. Absolute 3D shape profilometry usually requires multiple fringe pattern projections, which requires that the measurement conditions remain invariant while sequential measurements are performed. However, harsh operating environments where the object or the metrology system cannot be maintained in a steady-state may make such active strategies a luxurious or even unreasonable request. Under such conditions, conventional optical metrology approaches will suffer from severe physical and technical limitations, such as a limited amount of data and uncertainties in the forward model.

To address these challenges, researchers have made great efforts to improve state-of-the-art methods from different aspects over the past few decades. For example, phase-shifting techniques were optimized from the perspective of signal processing to achieve high-precision robust phase measurement and meanwhile minimize the impact of experimental perturbations^32,153^. Single-shot spatial phase-demodulation methods have been explicitly formulated as a constrained optimization problem similar to Eq. (9) with an extra regularization term enforcing a priori knowledge about the recovered phase (spatially smooth, limited spectral extension, piecewise constant, etc.)^140,148^. Multi-frequency temporal phase unwrapping techniques have been optimized by utilizing the inherent information redundancy in the average intensity and the intensity modulation of the fringe images, allowing for absolute phase retrieval with the reduced number of patterns^32,295^. Geometric constraints were introduced in FPP to solve the phase ambiguity problem without additional image acquisition^175,183^. Despite these extensive research efforts for decades, how to extract the absolute (unambiguous) phase information, with the highest possible accuracy, from the minimum number (preferably single shot) of fringe patterns remains one of the most challenging open problems in optical metrology. Consequently, we are looking forward to innovations and breakthroughs in the principles and methods of optical metrology, which are of significant importance for its future development.

### Solving inverse optical metrology problems via deep learning

**Fig. 9:**
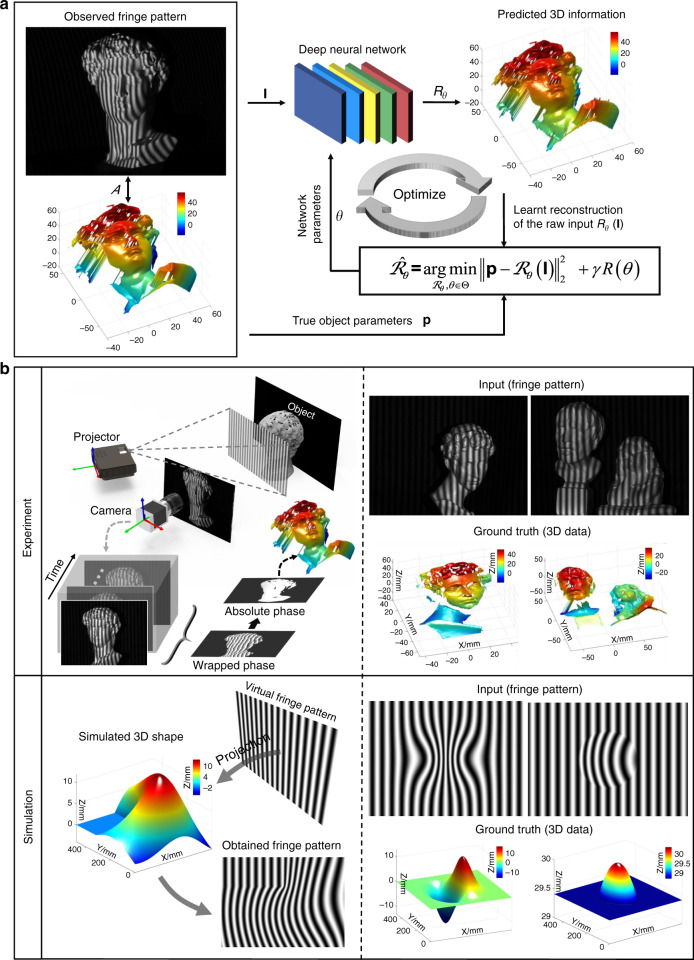
Deep-learning-based optical metrology as a constraint optimization problem. **a** In deep-learning-based optical metrology, a set of true object parameters **p** and the corresponding raw measured data **I** are created at the training stage, and their mapping relation (learn a reconstruction algorithm) ${{{\mathcal{R}}}}_\theta$ is established by training a deep neural network with all network parameters *θ* (neural network weights) learned from the dataset. **b** The principle of obtaining the dataset by real experiments or simulations with the knowledge of the forward model ${{{\mathcal{A}}}}$ (left) and the obtained dataset (right)

As a “data-driven” technology that has emerged in recent years, deep learning has received increasing attention in the field of optical metrology and made fruitful achievements in very recent years. Different from the conventional physical model and knowledge-driven approaches that the objective function (Eqs. (9) and (10)) is built based on the image formation model ${{{\mathcal{A}}}}$, in deep-learning approaches, we create a set of true object parameters **p** and the corresponding raw measured data **I**, and establish their mapping relation ${{{\mathcal{R}}}}_\theta$ based on a deep neural network with all network parameters *θ* learned from the dataset by solving the following optimization problem (Fig. 9):

11 $$\widehat {{{{\mathcal{R}}}}_\theta } = \mathop {{{{{\mathrm{argmin}}}}}}\limits_{{{{\mathcal{R}}}}_\theta ,\theta \in \Theta } \left\| {{{{\mathbf{p}}}} - {{{\mathcal{R}}}}_\theta \left( {{{\mathbf{I}}}} \right)} \right\|_2^2\, +\, R\left( \theta \right)$$

with $\left\| {}\, \right\|_2^2$ being the *L*2-norm error (loss) function once again (different types of loss functions discussed in the subsection “Neural network training” can be specified depending on the type of training data) and *R* is a regularizer of the parameters to avoid overfitting. A key element in deep-learning approaches is to parameterize $\widehat {{{{\mathcal{R}}}}_\theta }$ by parameters $\theta \in \Theta$. The “learning” process refers to finding an “optimal” set of network parameters from the given training data by minimizing Eq. (11) over all possible network parameters $\theta \in \Theta$. And the “optimality” is quantified through the loss function that measures the quality of the learned ${{{\mathcal{R}}}}_\theta$. Different deep-learning approaches can be thought of as different ways to parameterize the reconstruction network ${{{\mathcal{R}}}}_\theta$. Different from conventional approaches that solving the optimization problem directly gives the final solution $\widehat {{{{\mathcal{R}}}}_\theta }$ to the inverse problem corresponding to a current given input, in deep-learning-based approaches, the optimization problem is phrased as to find a “reconstruction algorithm” $\widehat {{{{\mathcal{R}}}}_\theta }$ satisfying the pseudo-inverse property $\widehat {{{\mathbf{p}}}} = \widehat {{{{\mathcal{R}}}}_\theta }\left( {{{\mathbf{I}}}} \right) = \tilde {{{\mathcal{A}}}}^{ - 1}\left( {{{\mathbf{I}}}} \right) \approx {{{\mathbf{p}}}}$ from the prepared (previous) dataset, which is then used for the reconstruction of the future input.

Most of the deep-learning techniques currently used in optical metrology belong to supervised learning, i.e., a matched dataset of ground-truth parameters **p** and corresponding measurements **I** should be created to train the network. Ideally, the dataset should be collected by physical experiments based on the same metrology system to account for all experimental conditions (which are usually difficult to be fully described by the forward image formation model). The ground truth can be obtained by measuring various samples that one is likely to encounter by employing active strategies mentioned above, without considering the ill-posedness of the real problem. To be more precise, in deep-learning-based optical metrology approaches, active strategies frequently used in conventional optical metrology approaches are shifted from the actual measurement stage to the preparation (network training) stage. Although the situation faced during the preparation stage may be different from that in the actual measurement stage, the information obtained in the former can be transferred to the latter in many cases. What we should do during the training stage is to reproduce the sample (using representative test objects), the system (using the same measurement system), and the error sources (noise, vibration, background illumination) during the measurement stage to ensure that the captured input data is as close as possible to those in the real measurement. On the other hand, we should make the remaining environmental variables as controllable as possible so that more active strategies (sample manipulation, illumination changing, multiple acquisitions) can be involved in the training stage to derive the ground truth corresponding to these captured data. Once the network is trained, we can then strip out these ideal environment variables and make the network run in a realistic experimental condition.

For example, for an interferometric system working in a harsh environment or a FPP system designed for measuring dynamic objects, phase demodulation from a single-fringe pattern is the most desirable choice. The inherent ill-posedness of the problem makes it a very good example for deep learning in this regard. In the training stage, we reproduce all the experimental conditions except that we employ the multi-frame phase-shifting technique with large phase-shifting steps to obtain the ground truth for the training samples. Once the network is established, it can map from only one single-fringe pattern to the desired phase distribution, and thus can be used in harsh environments where the single-shot phase-demodulation technique should be applied. Note that in this example, all the training data is fully generated by experiments, so the reconstruction algorithm (inverse mapping) $\widehat {{{{\mathcal{R}}}}_\theta }$ can be established without the knowledge of the forward model ${{{\mathcal{A}}}}$ in principle. Even though, since we have sufficient real-world training observations of the form (**p**, **I**), it can be expected that those experimental data can reflect the true ${{{\mathcal{A}}}}$ in a complete and realistic way.

It should be noted that there are also many cases that the ground truth corresponding to the experimental data is inaccessible. In such cases, the matched dataset can be obtained by a “learning from simulation” scheme — simulating the forward operator (with the knowledge of the forward image formation model ${{{\mathcal{A}}}}$) on ideal sample parameters. However, due to the complexity of real experimental conditions, we typically only know an approximation of ${{{\mathcal{A}}}}$. Subsequently, the inconsistency or uncertainty in the forward operator ${{{\mathcal{A}}}}$ may lead to a compromised performance in real experiments (see the “Challenges” section for detailed discussions). On the other hand, partial knowledge of the forward model ${{{\mathcal{A}}}}$ can be leveraged and incorporated in the deep neural network design to alleviate the “black box” nature of conventional neural network architectures, which may reduce the amount of required training data and provide more accurate and reliable network reconstruction (see the “Future directions” section for more details).

### Advantages of invoking deep learning in optical metrology

**Fig. 10:**
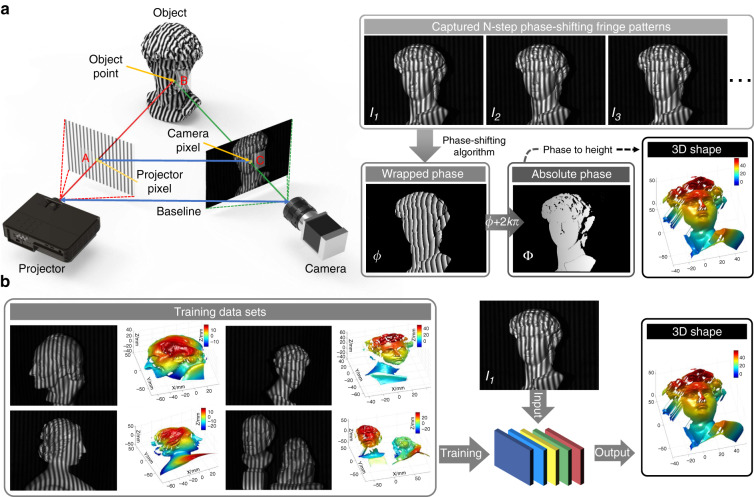
The advantages of deep-learning method compared with the traditional algorithm in the field of FPP. **a** The basic principle of FPP relies on the physical model of optical triangulation (left). The basic reconstruction steps generally include fringe projection, phase retrieval, phase unwrapping, and phase-to-height conversion based on calibrated system parameters. **b** The deep-learning-based FPP technology is driven by extensive training data. A well-trained deep-learning model can directly predict the depth information from a single-fringe image

In light of the above discussions, we summarize the potential advantages that can be gained by using a deep-learning approach in optical metrology. Figure 10 shows the advantages of deep-learning techniques compared to traditional optical metrology algorithms by taking FPP as an example. One may have noticed that FPP has appeared a few times, and in fact, it will appear more times. The reason is that FPP is currently one of the most promising and well-researched areas at the intersection of deep learning and optical metrology, offering a representative and convincing example of the use of deep learning in optical metrology.

**(1) From “physics-model-driven” to “data-driven”** Deep learning subverts the conventional “physics-model-driven” paradigm and opens up the “data-driven” learning-based representation paradigm. The reconstruction algorithm (inverse mapping) $\widehat {{{{\mathcal{R}}}}_\theta }$ can be learned from the experimental data without resorting to the pre-knowledge of the forward model ${{{\mathcal{A}}}}$. If the training data is collected under an environment that reproduces the real experimental conditions (including metrology system, sample types, measurement environment, etc.), and the amount (diversity) of data are sufficient, the trained model $\widehat {{{{\mathcal{R}}}}_\theta }$ should reflect the true ${{{\mathcal{A}}}}$ more precisely and comprehensively and is expected to produce better reconstruction results than conventional physics-model-driven or knowledge-driven approaches. The “data-driven” learning-based paradigm eliminates the need to design different processing flows for specific image-processing algorithm based on experience and pre-knowledge. By applying different types of training datasets, one specific class of neural network can be trained to perform various types of transformation for different tasks, significantly improving the universality and reducing the complexity of solving new problems.

**(2) From “divide-and-conquer” to “end-to-end learning”** In contrast to the traditional optical metrology approach that solves the sequence of tasks independently, deep learning allows for an “end-to-end” learning structure, where the neural network can learn the direct mapping relation between the raw image data and the desired sample parameters in one step, i.e., $\widehat {{{\mathbf{p}}}} = \widehat {{{{\mathcal{R}}}}_\theta }\left( {{{\mathbf{I}}}} \right)$, as illustrated in Fig. 10b. Compared with the “divide-and-conquer” scheme, the “end-to-end” learning allows to jointly solve multiple tasks, with great potential to alleviate the total computational burden. Such an approach has the advantage of synergy: it enables sharing information (features) between parts of the network that perform different tasks, which is more likely to get better overall performance compared to solving each task independently.

**(3) From “solving ill-posed inverse problems” to “learning pseudo-inverse mapping”** Deep learning utilizes complex neural network structures and nonlinear activation functions to extract high-dimensional features of the sample data, remove irrelevant information, and finally establish a nonlinear pseudo-inverse mapping model that is sufficient to describe the entire measurement process. The major reason for the success of deep learning is the abundance of training data and the explicit agnosticism from a priori knowledge of how such data are generated. Instead of hand-crafting a regularization function or specifying prior, deep learning can automatically learn it from the example data. Consequently, the learned prior *R*(*θ*) is tailored to the statistics of real experimental data and, in principle, provides stronger and more reasonable regularization to the inverse problem pertaining to a specific metrology system. Consequently, the obstacle of “solving nonlinear ill-posed inverse problems” can be bypassed, and the pseudo-inverse mapping relation between the input and the desired output can be established directly.

## The use of deep learning in optical metrology

### Deep-learning-enabled image processing in optical metrology

**Fig. 11:**
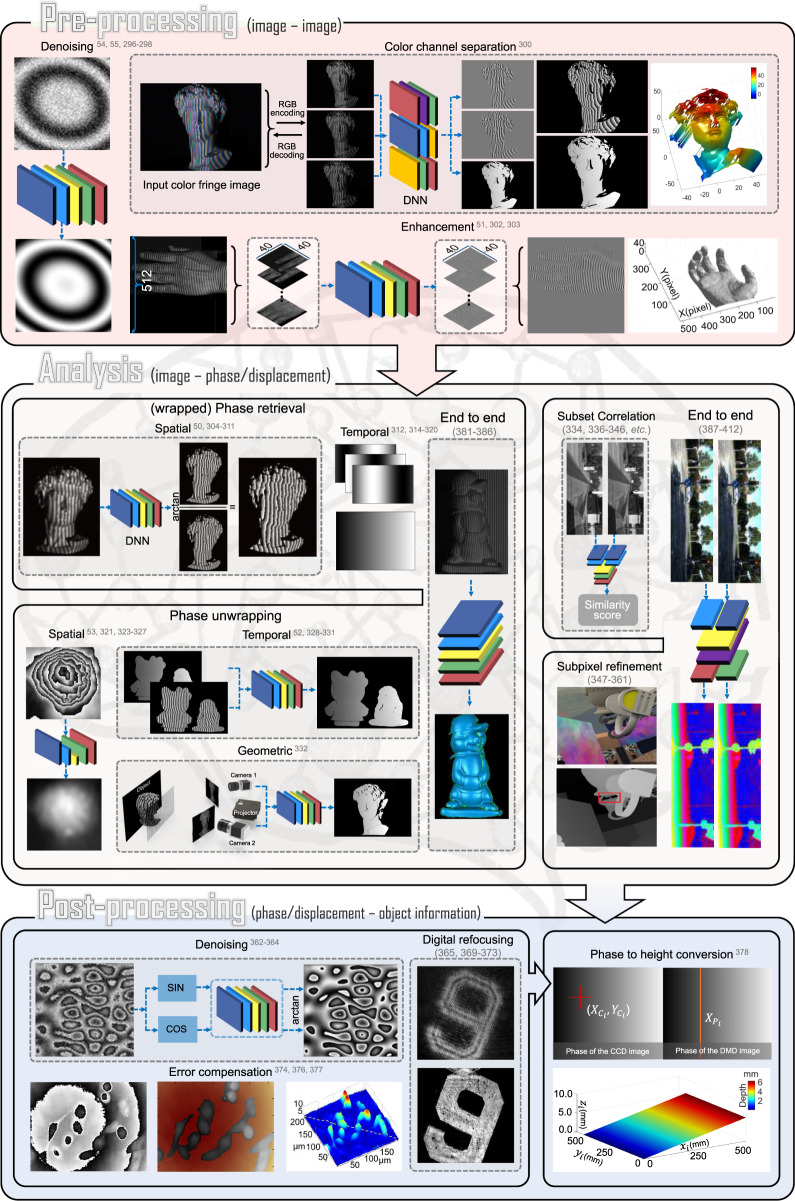
Deep learning in optical metrology. Because of the significant changes that deep learning brings to the concept of optical metrology technology, almost all elementary tasks of digital image processing in optical metrology have been reformed by deep learning

Owing to the above-mentioned advantages, deep learning has been gaining increasing attention in optical metrology, demonstrating promising performance in various optical metrology tasks and in many cases exceeding that of classic techniques. In this section, we review these existing researches leveraging deep learning in optical metrology according to an architecture similar to that introduced in the section “Image processing in optical metrology”, as summarized in Fig. 11. The basic network types, loss functions, and data acquisition methods of some representative examples are listed in Table 1.

**Fig. 12:**
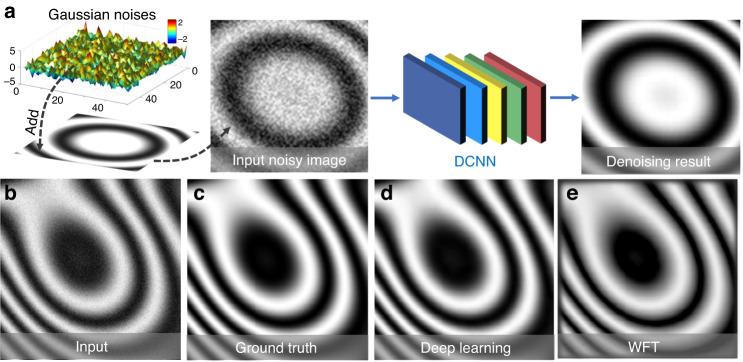
Flowchart of deep-learning-based fringe pattern denoising and the denoising results of different methods. **a** The flowchart of deep-learning-based fringe pattern denoising method: taking noisy fringe patterns as input to DCNN and predicting the denoised image directly. **b** The noisy input pattern. **c** Ground truth. **d** The predicted result of deep learning. **e** The denoising result of WFT^114^. **a**–**e** Adapted with permission from ref. ^55^, Copyright (2021), with permission from Elsevier

**Fig. 13:**
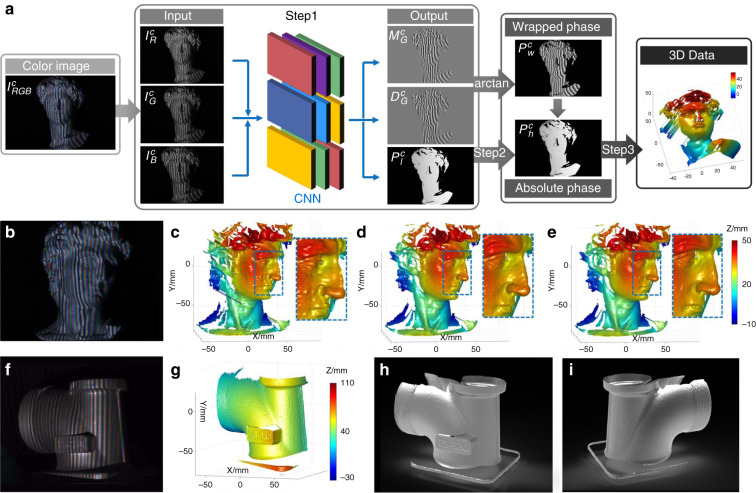
Flowchart of deep-learning-based color FPP and the 3D reconstruction results of different approaches. **a** The flowchart of deep-learning-based color FPP: CNN predicts the sine and cosine terms related to high-quality wrapped phase map from the input 3-channel fringe images of different frequencies, as well as a “coarse” absolute phase map. Then the outputs of CNN are used to obtain a high-accuracy absolute phase for further 3D reconstruction. **b** The input color fringe pattern of a David plaster model. **c** The 3D reconstruction result of the color-coded approach proposed by Zhang et al.^301^. **d** The 3D reconstruction result of our deep-learning-based method. **e** Ground truth. **f** One frame of the color fringe patterns of a 360° rotated workpiece. **g** 3D result of (**f**). **h**, **f** Registration results viewed from two different perspectives. **a**–**i** Adapted with permission from ref. ^300^, Optica Publishing

**Fig. 14:**
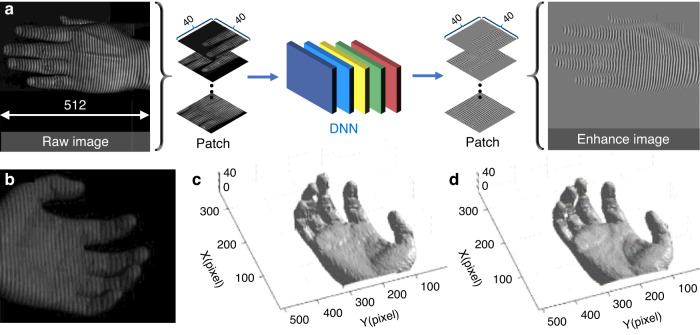
Flowchart of the deep-learning-based fringe-enhancement method and the 3D reconstruction results of different approaches. **a** The flowchart of the deep-learning-based fringe enhancement: the captured raw fringe images and the quality-enhanced versions are used to learn the mapping between the input fringe image and the output enhanced fringe part of the constructed DnCNN. **b** Input raw fringe pattern of a moving hand. **c** 3D reconstruction result obtained by traditional FT^138^. **d** 3D reconstruction result obtained by the deep-learning method. **a**–**d** Adapted with permission from ref. ^51^, Optica Publishing

**Fig. 15:**
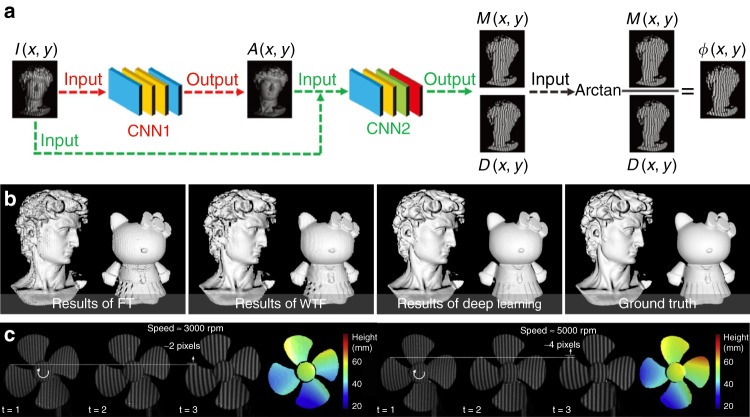
Flowchart of the single-frame phase retrieval approach using deep learning and the 3D reconstruction results of different approaches. **a** The principle of deep-learning-based phase retrieval method: the background image A is first predicted from the single-frame fringe image I through CNN1; then CNN2 is employed to realize the mapping between the fringe pattern I and the predicted background map A to the numerator term M and the denominator term D of the arctangent function; finally, the high-accuracy wrapped phase map can be obtained by the arctangent function. **b** Comparison of the 3D reconstructions of different fringe analysis approaches (FT^138^, WFT^114^, the deep-learning-based method, and 12-step phase-shifting profilometry). **c** The measurement results of a desk fan rotating at different speeds using our deep-learning method. **a**, **b** Adapted from ref. ^50^. Distributed under Creative Commons (CC BY 4.0) license https://creativecommons.org/licenses/by/4.0/legalcode. **c** Adapted with permission from ref. ^304^, Copyright (2021), with permission from Elsevier

**Fig. 16:**
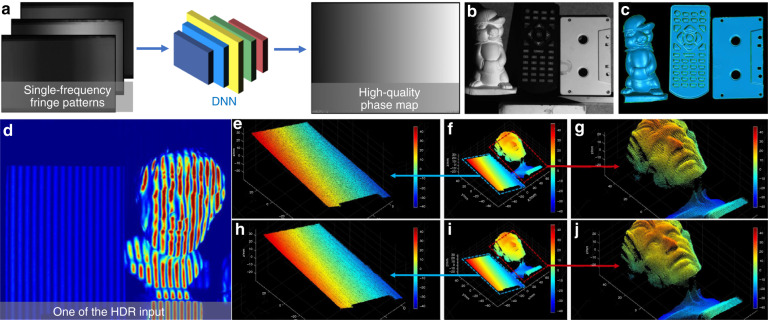
Temporal phase retrieval based on deep learning. **a** Schematic of using deep learning for temporal phase retrieval using three phase-shifted sinusoidal fringe images. **b**, **c** The reconstruction results of a complex scene based on the deep-learning method illustrated in (**a**). **d** One raw image containing both low-modulation and saturated fringes. **e**–**g** The 3D reconstruction results using the traditional phase-shifting method. **h**–**j** The 3D reconstruction results using the deep-learning-based HDR method. **a**–**c** Adapted with permission from ref. ^312^, Optica Publishing. **d**–**j** Adapted with permission from ref. ^317^, Copyright (2021), with permission from Elsevier

**Fig. 17:**
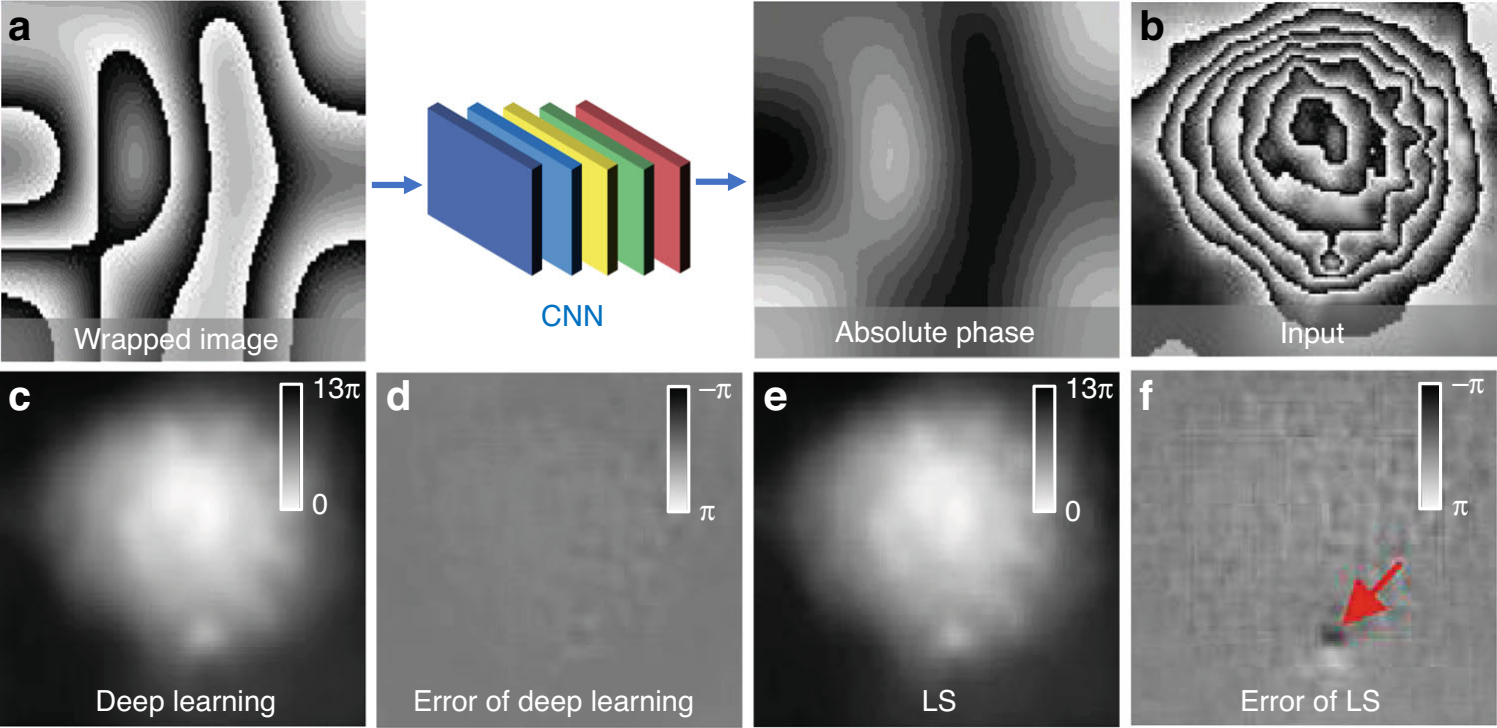
Flowchart of the one-step deep-learning-based phase unwrapping approach and the unwrapping results of different methods. **a** The flowchart of the one-step deep-learning-based phase unwrapping approach: the absolute phase can be predicted directly from a noisy wrapped phase based on the trained CNN. **b** The wrapped phase map of living mouse osteoblast. **c** Unwrapped phase of (**b**) obtained by deep learning. **d** Phase errors of (**c**). **e** Unwrapped phase of (**b**) obtained by the conventional LS method^322^. **f** Phase errors of (**e**). **a**–**f** Adapted with permission from ref. ^321^, Optica publishing

**Fig. 18:**
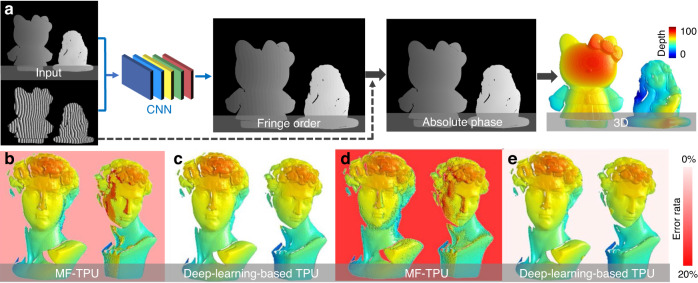
Flowchart of deep-learning-assisted temporal phase unwrapping method and the 3D reconstructions of different phase unwrapping approaches. **a** The flowchart of deep-learning-based temporal phase unwrapping. **b** The 3D reconstruction obtained from phase unwrapping of (1 + 32)-frequency combination by MF-TPU^164^. **c** The 3D reconstruction obtained from phase unwrapping of (1 + 32)-frequency combination by the deep-learning-based method. **d** The 3D reconstruction obtained from phase unwrapping of (1 + 64)-frequency combination by MF-TPU. **e** The 3D reconstruction after phase unwrapping of (1 + 64)-frequency combination by deep-learning-based TPU. **a**–**e** Adapted by permission from Springer Nature: Scientific Reports^52^, Copyright (2021)

**Fig. 19:**
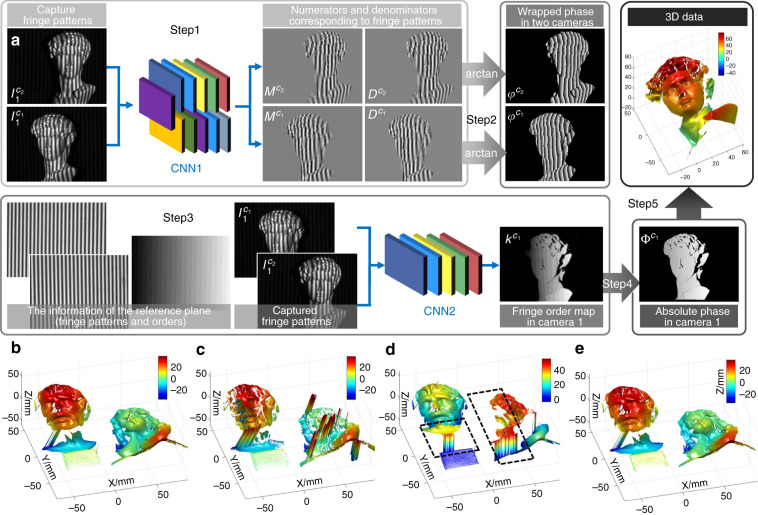
Flowchart of deep-learning-assisted geometric constraints and phase unwrapping and the 3D reconstruction results of different methods. **a** The flowchart of deep-learning-assisted geometric constraints and phase unwrapping: Feng’s method^50^ is applied to extract the wrapped phases from two perspectives through CNN1. Then the single-frame dual-view fringe patterns, as well as the reference information are fed into CNN2 to output the fringe orders. Through the predicted wrapped phase data and fringe orders, the absolute phase in the left perspective can be recovered, followed by 3D reconstruction. **b** The result obtained by combining phase-shifting, triple-camera geometric phase unwrapping, and adaptive depth-constraint methods^186^. **c** The result obtained by combining phase-shifting and dual-camera geometric phase unwrapping methods. **d** The result obtained by An’s depth-constraint method^179^. **e** The result obtained by the deep-learning-enabled geometric constraint method. **a**–**e** Adapted from ref. ^332^, with the permission of AIP Publishing

1. **Pre-processing:** Many early works applying deep learning to optical metrology focused on image pre-processing tasks, such as denoising and enhancement. This is mainly due to the fact that the successful use cases of deep learning to such pre-processing tasks can be easily found in the computer vision community. Many image pre-processing algorithms in optical metrology could receive a performance upgrade by simply reengineering these existing neural network architectures for a similar kind of problem.
  **Denoising**: Yan et al.^55^ constructed a CNN composed of 20 convolutional layers for fringe denoising (Fig. 12a). Simulated fringe patterns with artificial Gaussian noise were generated as the training dataset, and corresponding noise-free versions were used as ground truth. Figure 12d, e shows the denoising results of WFT^114^ and the deep-learning-based method, showing that their method was free of the boundary artifacts in WFT and achieved comparable denoising performance in the central region. Jeon et al.^296^ proposed a fast speckle-noise reduction method based on U-Net, which showed robust and excellent denoising performance for digital holographic images. Hao et al.^54^ constructed a fast and flexible denoising convolutional neural network (FFDNet) for batch denoising of ESPI fringe images. Lin et al.^297^ developed a denoising CNN (DnCNN) for speckle-noise suppression of fringe patterns. Reyes-Figueroa and Rivera^298^ proposed a fringe pattern filtering and normalization technique based on autoencoder^299^. The autoencoder was able to fine-tune the U-Net network parameters and reduce residual errors, thereby improving the stability and repeatability of the neural network. Since it is difficult to access noise-free ground-truth images in real experimental conditions, the training datasets of these deep-learning-based denoising methods are all generated based on simulations.
  **Color channel separation:** Our group reported a single-shot 3D shape measurement approach with deep-learning-based color fringe projection profilometry that can automatically eliminate color cross-talk and channel imbalance^300^. As shown in Fig. 13a, the network predicted the sine and cosine terms related to high-quality cross-talk-free phase information from the input 3-channel fringe images of different wavelengths. In order to get rid of color cross-talk and chromatic aberration, the green monochromatic fringe patterns were projected and only the green channel of the captured patterns was used to generate labels. Figure 13b–d shows 3D reconstruction results of a David plaster model measured by the traditional color-coded method^301^ and our method, showing that the deep-learning-based method yielded more accurate surface details. The quality of the 3D reconstruction was comparable to the ground truth (Fig. 13e) obtained by the non-composite (monochromatic) multi-frequency phase-shifting method^174^. The deep-learning-based method was applied for dynamic 360° 3D digital modeling, demonstrating its potential in rapid reverse engineering and related industrial applications (Fig. 13f–i).
  **Enhancement**: Shi et al.^51^ proposed a fringe-enhancement method based on deep learning, and the flowchart of which is given in Fig. 14a. The captured fringe image and the corresponding enhanced one obtained by the subtraction of two fringe patterns with *π* relative phase shift were used to establish the mapping between the raw fringe and the desired enhanced versions. Figure 14b–d shows the 3D reconstruction results of a moving hand using the traditional FT method^138^ and the deep-learning method, suggesting that the deep-learning method outperformed FT in terms of detail preservation and SNR. Goy et al.^302^ proved that DNN could recover an image with decent quality under low-photon conditions, and successfully applied their method to phase retrieval. Yu et al.^303^ proposed a fringe-enhancement method in which the fringe modulation was improved by deep learning, facilitating high-dynamic 3D shape measurement without resorting to conventional multi-exposure schemes.
2. **Analysis**: Image analysis is the most critical step in the image-processing architecture of optical metrology. Consequently, most deep-learning techniques applied to optical metrology are proposed to accomplish the tasks associated with image analysis. For phase measurement techniques, deep learning is extensively explored for (both spatial and temporal) phase demodulation and (spatial, temporal, and geometric) phase unwrapping.
  **Phase demodulation:**
    **Spatial phase retrieval**: To address the contradiction between the measurement efficiency and accuracy of traditional phase retrieval methods, our group, for the first time, introduced deep learning to fringe pattern analysis, substantially enhancing the phase-demodulation accuracy from a single-fringe pattern^50^. As illustrated in Fig. 15a, the background image A was first predicted from the acquired fringe image I through CNN1. Then CNN2 was employed to realize the mapping from I and A to the numerator (sine) term M and denominator (cosine) term D. Finally, the wrapped phase information can be acquired by computing the arctangent of M/D. Figure 15b compares the phases retrieved by two representative traditional single-frame phase retrieval methods (FT^138^, WFT^114^) and the deep-learning method, revealing that our deep-learning-based single-frame phase retrieval method achieved the highest reconstruction quality, which almost visually reproduced the ground-truth information obtained by the 12-step phase-shifting method. We have incorporated the deep-learning-based phase retrieval technique into the micro-Fourier transform profilometry (*μ*FTP) technique to eliminate the need for additional uniform patterns, doubling the measurement speed and achieving an unprecedented 3D imaging frame rate up to 20,000 Hz^304^. Figure 15c shows the 3D measurement results of a rotating fan at different speeds (3000 and 5000 revolutions per minute (RPM)), suggesting that the 3D shape of fan blades can be intactly reconstructed without any motion-induced artifacts visible. Qiao et al.^305^ applied this deep-learning-based phase extraction technique for phase measuring deflectometry, and achieved single-shot high-accuracy 3D shape measurement of specular surfaces. Some other network structures, such as structured light CNN (SL-CNN)^306^ and deep convolutional GAN^307^ were also adopted for single-frame phase retrieval. In addition, deep learning can also be applied to Fourier transform profilometry for automatic spectrum extraction by identifying the carrier frequency components bearing the object information in the Fourier domain, facilitating automatical spectrum extraction, and achieving higher phase retrieval accuracy without human intervention^308^. Wang et al.^309^ proposed an automatical holographic reconstruction framework (Y-Net) consisting of two symmetrical U-Nets, allowing for simultaneous recovery of phase and intensity information from a single off-axis digital hologram. They also doubled the capability of Y-Net, extending it to the reconstruction of dual-wavelength complex amplitudes, while overcoming the spectral overlapping issue in common-path dual-wavelength digital holography^310^. Recently, our group used U-Net to realize aliasing-free phase retrieval from a dual-frequency composite fringe pattern^311^. Compared with the traditional Fourier transform profilometry, the deep-learning-enabled approach avoids the complexities associated with dual-frequency spectra separation and extraction, allowing for higher-quality single-shot absolute 3D shape reconstruction.
    **Temporal phase retrieval**: Wang et al.^312^ introduced a deep-learning scheme to the phase-shifting technique in FPP. As shown in Fig. 16a, by introducing a fully connected DNN, the link between three low- and unit-frequency phase-shifting fringe patterns and high-quality absolute phases calculated from high-frequency fringe images were established, and thus, the 3D measurement accuracy could be significantly enhanced. The three unit-frequency phase-shifting patterns were encoded in three monochrome channels of a color image and projected by a 3LCD projector. The individual fringe patterns were then decoded and projected by the projector sequentially and rapidly^313,314^. Consequently, the hardware system allowed for real-time 3D surface imaging of multiple objects at a speed of 25.6 fps. Zhang et al.^315^ developed a deep-phase-shift network (DPS-Net) based on GAN, with which multi-step phase-shifting interferograms with accurate arbitrary phase shifts for calculating high-quality phase information were predicted from a single interferogram. Besides random intensity noise, conventional phase-shifting algorithms are also sensitive to other experimental imperfections, such as phase-shifting error, illumination fluctuations, intensity nonlinearity, lens defocusing, motion-induced artifacts, and detector saturation. Deep learning also provides a potential solution to eliminate or at least partially alleviate the impact of these error sources on phase measurement. For example, Li et al.^316^ proposed a deep-learning-based phase-shifting interferometric phase recovery approach. The constructed U-Net was capable of predicting the accurate wrapped phase map from two interferogram inputs with unknown phase shifts. Zhang et al.^317^ applied CNN to extract a high-accuracy wrapped phase map from conventional 3-step phase-shifting fringe patterns. In the training stage, low-modulation or saturated fringe patterns were used as the raw dataset, and the relation between these imperfect raw fringe and high-quality error-free unwrapped phase (obtained by 12-step phase-shifting algorithms) were established based on CNN. Consequently, the deep-learning-based approach could accommodate both dark and reflective surfaces, and the related phase errors (noise and saturation) in the conventional three-step phase-shifting method were significantly suppressed, making it a promising approach for high-dynamic-range (HDR) 3D measurement of surfaces with large reflectivity variations (Fig. 16d–g). Wu et al.^318^ proposed a deep-learning-based phase-shifting approach to overcome the phase errors associated with intensity nonlinearity. Through a well-trained FCN, the distortion-free high-quality phase map could be reconstructed conveniently and efficiently from the raw phase-shifting fringe patterns with a strong gamma effect. Yang et al.^319^ constructed a three-to-three deep-learning framework (Tree-Net) based on U-Net to compensate for the nonlinear effect in the phase-shifting images, which effectively and robustly reduced the phase errors by about 90%. Recently, our group demonstrated that the nonsinusoidal errors (e.g., residual high-order harmonics in binary defocusing projection, intensity saturation, gamma effect of projectors and cameras, and their coupling) in phase-shifting profilometry could be handled by an integrated deep-learning framework. A well-trained U-Net could effectively suppress the phase errors caused by different types of nonsinusoidal fringe with only a minimum of three fringe patterns as input^320^.
  **Phase unwrapping:**
    **Spatial phase unwrapping:** Wang et al.^321^ proposed a one-step phase unwrapping approach based on deep learning. Various ideal (noise-free) continuous phase distributions and the corresponding wrapped phase maps with different types of noises (Gaussian, salt and pepper, or multiplicative noises) were simulated and used as the training dataset for a CNN based on U-Net. Upon completion of the training, the absolute phases can be predicted directly from a noisy wrapped phase map, as illustrated in Fig. 17a. Figure 17b–f shows the comparisons of phase unwrapping results obtained by the traditional least-square (LS) method^322^ and deep-learning-based method, demonstrating that deep learning can directly fulfill the complicated nonlinear phase unwrapping task in one step with improved anti-noise and anti-aliasing ability. Spoorthi et al.^323^ developed a CNN-based phase unwrapping framework-PhaseNet. The fringe order (2*π* integer phase jumps) used for phase unwrapping can be obtained pixel by pixel through a semantic segmentation-based deep-learning framework of the encoder-decoder structure. Recently, they developed an enhanced phase unwrapping framework—PhaseNet 2.0, which could directly map a noisy wrapped phase to a denoised absolute one^324^. Zhang et al.^325^ transferred the task of phase unwrapping to a multi-class classification problem and generated fringe orders by feeding the wrapped phase into a convolutional segmentation network. Zhang et al.^53^ proposed a deep-learning-based approach for rapid 2D phase unwrapping, which demonstrated good denoising and unwrapping performance and outperformed the conventional path-dependent and path-independent methods. Kando et al.^326^ applied U-Net to achieve absolute phase prediction from a single interferogram, and the quality of the recovered phase was superior to that obtained by the conventional FT method, especially for closed-fringe patterns. Li et al.^327^ proposed a deep-learning-based phase unwrapping strategy for closed fringe patterns. They compared four different network structures for phase unwrapping and found that the improved FCN architecture performed the best in terms of accuracy and speed. However, it should be mentioned that, similar to the case of fringe denoising, true absolute phase maps corresponding to the real experimentally obtained wrapped phase maps are generally quite hard to obtain in many interferometric techniques (which requires sophisticated multi-wavelength illuminations and heterodyne operations). Therefore, the training datasets used in the above-mentioned deep-learning-based spatial phase unwrapping methods are generated based on numerical simulation instead of real experiments. Moreover, since only one single wrapped phase map is used as input, the above-mentioned deep-learning-based spatial phase unwrapping methods still suffers from the 2*π* ambiguity problem inherent in traditional phase measurement techniques.
    **Temporal phase unwrapping:** Our group developed a deep-learning-based temporal phase unwrapping framework, as illustrated in Fig. 18a^52^. The inputs of the network are a single-period (wrap-free) phase map and a high-frequency wrapped phase map, from which the constructed CNN could directly predict the fringe orders corresponding to the high-frequency phase to be unwrapped. Figure 18b–e gives the comparison between the traditional multi-frequency temporal phase unwrapping (MF-TPU) method^174^ and the deep-learning-based approach for the 3D reconstructions obtained by unwrapping the wrapped phase maps using the (1–32) and (1–64) frequency combination of fringe patterns, respectively. In comparison with MF-TPU, the deep-learning-assisted method produced phase unwrapping results with higher accuracy and robustness even in the case of different types of error sources (low SNR, intensity nonlinearity, and object motion). Liu et al.^328^ further improved this approach by using a lightweight classification CNN to extract the fringe orders from a pair of low-high-frequency phase maps, which saved a large amount of training time and made it possible to deploy the network on mobile devices. Li et al.^329^ proposed a deep-learning-based dual-wavelength phase unwrapping approach in which only a single-wavelength interferogram was used to predict another interferogram recorded at a different wavelength with a conditional GAN (CGAN). Though their approach still suffered from the phase ambiguity problem when measuring discontinuous surface or isolated objects, it provided an effective and potential solution to phase unwrapping and extended the measurement range of single-wavelength interferometry and holography techniques. Yao et al. designed FCNs by incorporating residual layers to predict the fringe orders of wrapped phases from only two^330^ or even single^331^ Gray-code image(s), significantly reducing the required images compared with the conventional Gray-code technique.
    **Geometric phase unwrapping:** Our group proposed a deep-learning-assisted geometric phase unwrapping approach for single-shot 3D surface measurement^332^. The flowchart of this approach is shown in Fig. 19a. Two CNNs (CNN1 and CNN2) were constructed for phase retrieval and phase unwrapping, respectively. Based on a stereo camera system, dual-view single-shot fringe patterns, as well as the reference plane images, were fed into CNN2 to determine the fringe orders. With the predicted wrapped phases and fringe orders, the absolute phase map can be recovered. Figure 19b–e is the comparison of 3D reconstructions obtained through different conventional geometric phase unwrapping methods^175,179,186^ and the deep-learning-based method, demonstrating that the deep-learning-based method can robustly unwrap the wrapped phases of dense fringe patterns within a larger measurement volume under the premise of single-frame projection. It should be mentioned that it is indeed a straightforward idea to establish the relationship between the fringe pattern to the corresponding absolute phase directly. However, since the rationality of the deep-learning-based approach is largely dependent on the input data, when the input fringe itself is ambiguous, the network can never always produce reliable phase unwrapped results. For example, in Yu’s work^333^, when there exist large depth discontinuities and isolated objects, even with the assistance of deep learning, one fringe image is insufficient to eliminate the 2*π* phase ambiguity.

**Table 1:** Basic network structures, loss functions, and data acquisition methods for deep-learning methods applied to optical metrology tasks

	Task	Reference	Network structure	Training database	Loss function
Pre-processing	Denoising	Yan et al.^55^	SRCNN	Simulation	MAE
		Jeon et al.^296^	U-Net + ResNet	Simulation	MAE
		Hao et al.^54^	SRCNN + ResNet	Simulation	Euclidean loss
		Lin et al.^297^	SRCNN + ResNet	Simulation	Euclidean loss
	Color channel separation	Qian et al.^300^	FCN + ResNet	Experiment	MSE
	Enhancement	Shi et al.^51^	SRCNN	Experiment	MSE
		Yu et al.^303^	FCN	Experiment	MSE
Analysis	Phase demodulation	Feng et al.^50,304^	FCN + ResNet	Experiment	MSE
		Yang et al.^307^	GAN	Experiment	GAN loss
		Li et al.^311^	U-Net	Experiment	MSE
		Zhang et al.^315^	GAN	Simulation	GAN loss
	Phase unwrapping	Wang et al.^321^	U-Net	Simulation	SSIM
		Spoorthi et al.^323^	FCN	Simulation	MSE
		Zhang et al.^325^	FCN + U-Net	Simulation	Cross-entropy
		Kando et al.^326^	U-Net	Simulation	RMSE
		Yin et al.^52^	FCN + ResNet	Experiment	MSE
	Subset correlation	Žbontar and LeCun^334^	CNN	KITTI^459^, Middlebury^460^	Hinge loss
		Luo et al.^336^	CNN	KITTI^459^	Cross-entropy
		Guo et al.^344^	FCN	Scene Flow^388^, KITTI^459^	Smooth L1
	Subpixel refinement	Pang et al.^347^	FCN	FlyingThings3D^387^, Middlebury^460^, KITTI^459^	MAE
		Hartmann et al.^338^	CNN + ResNet	Scene Flow^388^, KITTI^459^	Smooth L1
	Denoising	Montresor et al.^362^	SRCNN + ResNet	Simulation	MSE
		Yan et al.^363^	SRCNN + ResNet	Simulation	MSE
	Digital refocusing	Ren et al.^365^	SRCNN + ResNet	Experiment	MSE
		Wang et al.^309^	U-Net	Experiment	MSE
		Lee et al.^370^	CNN	Simulation	MSE
		Shinmobaba et al.^371^	CNN	Experiment	MSE
	Error compensation	Nguyen et al.^374^	U-Net	Experiment	Cross-entropy
		Aguénounon et al.^377^	U-Net	Experiment	Mse
Postprocessing	Phase to height conversion	Li et al.^378^	BP neural network	Experiment	–
End-to-end	From fringe to 3D shape	Nguyen et al.^381^	FCN, U-Net	Experiment	MSE
		Van et al.^382^	SRCNN	Simulation	RMSE
		Machineni et al.^384^	FCN + ResNet	Simulation	Smooth L1
		Zheng et al.^385^	U-Net	Simulation	RMSE
	From stereo images to disparity	Kendall et al.^389^	SRCNN + ResNet	Scene Flow, KITTI	MSE
		Chang et al.^390^	FCN + ResNet	Scene Flow, KITTI	Smooth L1

**Fig. 20:**
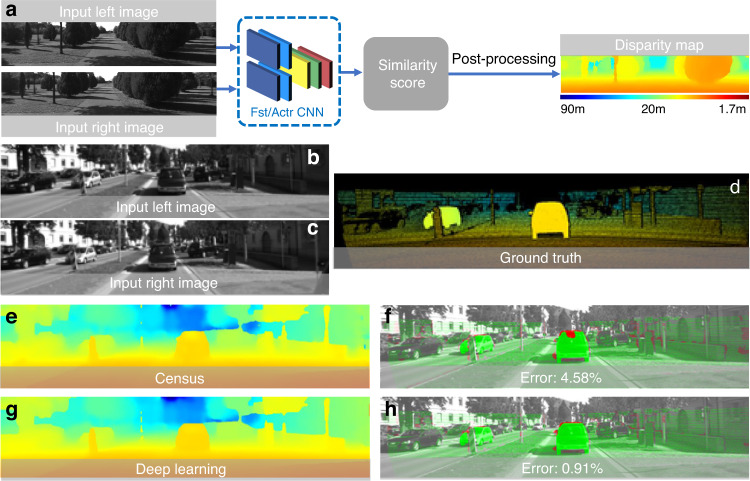
Flowchart of the deep-learning-based method for extracting depth information and the estimated disparity images using different methods. **a** The flowchart of deep-learning-based method for extracting depth information: two network architectures (one tuned for speed, the other for accuracy) are trained to learn the matching cost computation. The output of CNN is applied to initialize the stereomatching cost, followed by a series of postprocessing processes. **b**, **c** The input stereo images. **d** Ground truth. **e**, **g** The disparity estimation results using Census^335^ and CNN. **f**, **h** The disparity errors of (**e**, **g**). **a**–**h** Adapted from ref. ^334^. © 2016 Jure Zbontar and Yann LeCun, Microtome Publishing

**Fig. 21:**
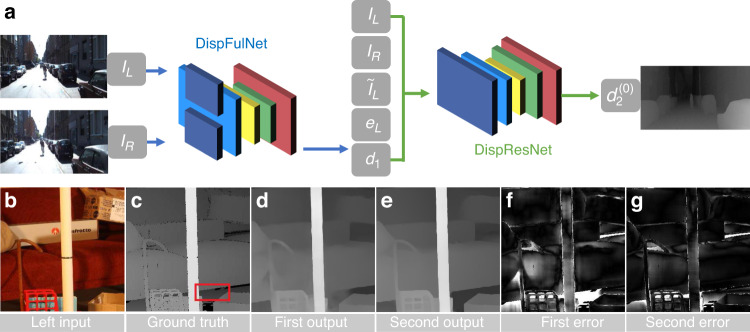
Flowchart of the cascade CNN architecture consisting of two stages for disparity estimation and the corresponding predicted disparities. **a** Flowchart of the cascade CNN architecture consisting of two stages for disparity estimation: the first stage outputs the disparity image with more details from the input stereo images through the DispFulNet, where *I*_*L*_ and *I*_*R*_ are the stereo pairs, *d*_1_ is the initial disparity, $\tilde I_L$ represents the synthesized left image and *e*_*L*_ is the error map between *I*_*L*_ and $\tilde I_L$. The second stage rectifies *d*_1_ and generates residual signals across multiple scales through the DispResNet, where $d_2^{(0)}$ is the new disparity of the scale of full resolution. The final disparity map is obtained by combining the outputs of the above two stages. **b** Left input. **c** Ground truth. **d**, **e** Outputs of the first stage and the second stage. **f**, **g** Error distributions between **c**, **d** and between (**c**) and (**e**). **a**–**g** ©(2021) IEEE. Adapted, with permission, from ref. ^347^

**Fig. 22:**
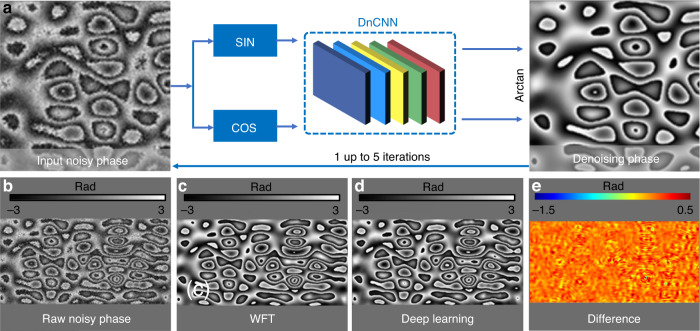
Flowchart of computational denoising based on deep learning for phase information and phase denoising results of different methods. **a** The flowchart of DnCNN-based phase denoising approach: the sine and cosine images of the noisy phase map is fed into a DnCNN to achieve the denoised phase information. To improve performance, 1–5 iterations are introduced in the denoising process. **b** The raw noisy phase. **c** The denoised phase processed with WFT^114^. **d** The denoised phase processed with deep learning. **e** The phase difference between (**c**) and (**d**). **a**–**e** Adapted with permission from ref. ^362^, AIP Publishing

**Fig. 23:**
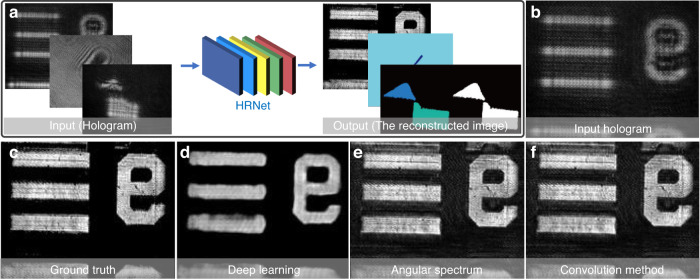
Flowchart of a holographic reconstruction network---HRNet and the hologram reconstruction results of different methods. **a** The flowchart of HRNet: Three different types of holograms (an amplitude object, a phase object, or a two-sectional object) can be used as input to obtain the corresponding reconstructions. **b** The input hologram image. **c** Ground truth. **d** The reconstructed images with deep learning. **e** The reconstructed images with the angular spectrum method.^368^ **f** The reconstructed images with the convolution method.^366^ **a**–**f** Adapted with permission from ref. ^365^. Distributed under Creative Commons (CC BY 4.0) license https://creativecommons.org/licenses/by/4.0/legalcode

**Fig. 24:**
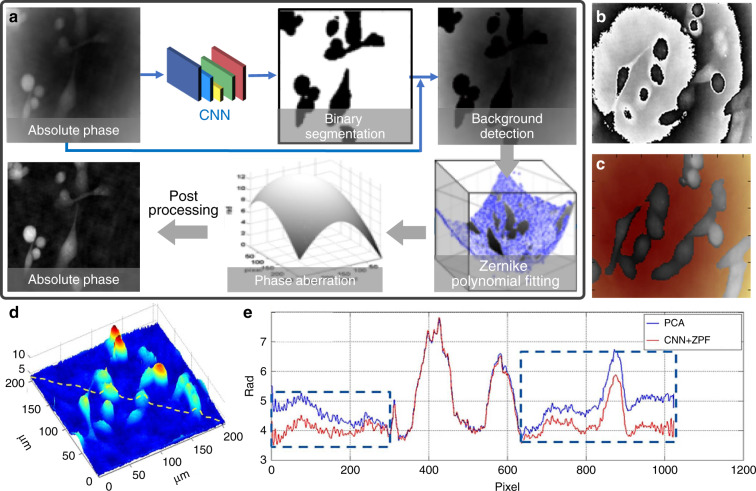
Flowchart of the phase-aberration compensation method combining Zernike polynomial fitting and deep learning, and the phase-aberration compensation results of different methods. **a** The flowchart of CNN + (Zernike polynomial fitting) phase-aberration compensation. **b** The input phase-aberration map. **c** The unwrapped phase overlaid with CNN’s output, where the background (color denoted) is fed into Zernike polynomial fitting. **d** The 3D phase after compensation by CNN + Zernike polynomial fitting. **e** The phase profile of PCA^226^ and CNN + Zernike polynomial fitting along the dashed line in (**d**). **a**–**e** Adapted with permission from ref. ^374^, Optica Publishing

**Fig. 25:**
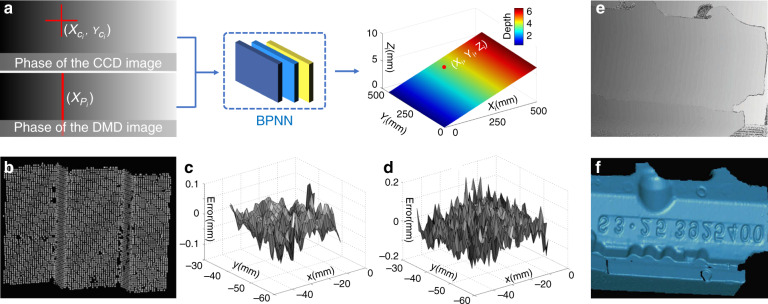
Flowchart of the learning-based phase-height mapping approach and 3D reconstruction results of different methods. **a** Flowchart of the learning-based phase-height mapping approach: the image coordinates (*X*_*ci*_, *Y*_*ci*_) and the corresponding projector lateral coordinates *X*_*pi*_ are fed into a 3-layer BP neural network, and the outputs are 3D coordinates (*X*_*i*_, *Y*_*i*_, *Z*_*i*_). **b** The 3D result of a standard stair sample predicted by the learning-based method. **c** The plane errors of the measurement result of a stair sample by the learning-based method. **d** The plane errors of the measurement result of a stair sample by traditional calibration method^380^. **e**, **f** The input absolute phase map of a workpiece and the corresponding 3D reconstruction. **a**–**e** Adapted with permission from ref. ^378^, Copyright (2021), with permission from Elsevier

**Fig. 26:**
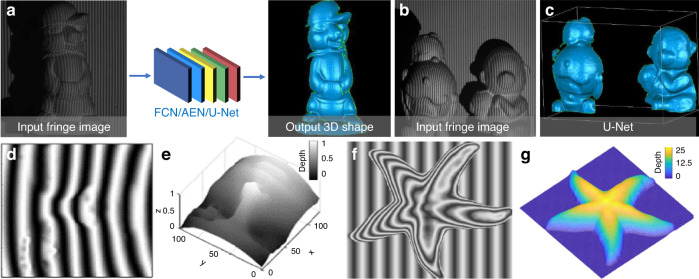
Flowchart of the single-shot end-to-end 3D shape reconstruction based on deep learning and the 3D reconstruction results. **a** Flowchart of the single-shot end-to-end 3D shape reconstruction based on deep learning: three different deep CNNs, including FCN, AEN^299^, and U-Net are constructed to perform the mapping of 2D images to its corresponding 3D shape^381^. **b**, **c** The input and output of Nguyen’s method^381^. **d**, **e** The input and output of Van’s method^382^. **f**, **g** The input and output of Machineni’s method^384^. **a**–**c** Adapted with permission from ref. ^381^, MDPI Publishing. **d**, **e** Adapted with permission from ref. ^382^, Optica Publishing. **f**, **g** Adapted with permission from ref. ^384^, Copyright (2021), with permission from Elsevier

**Fig. 27:**
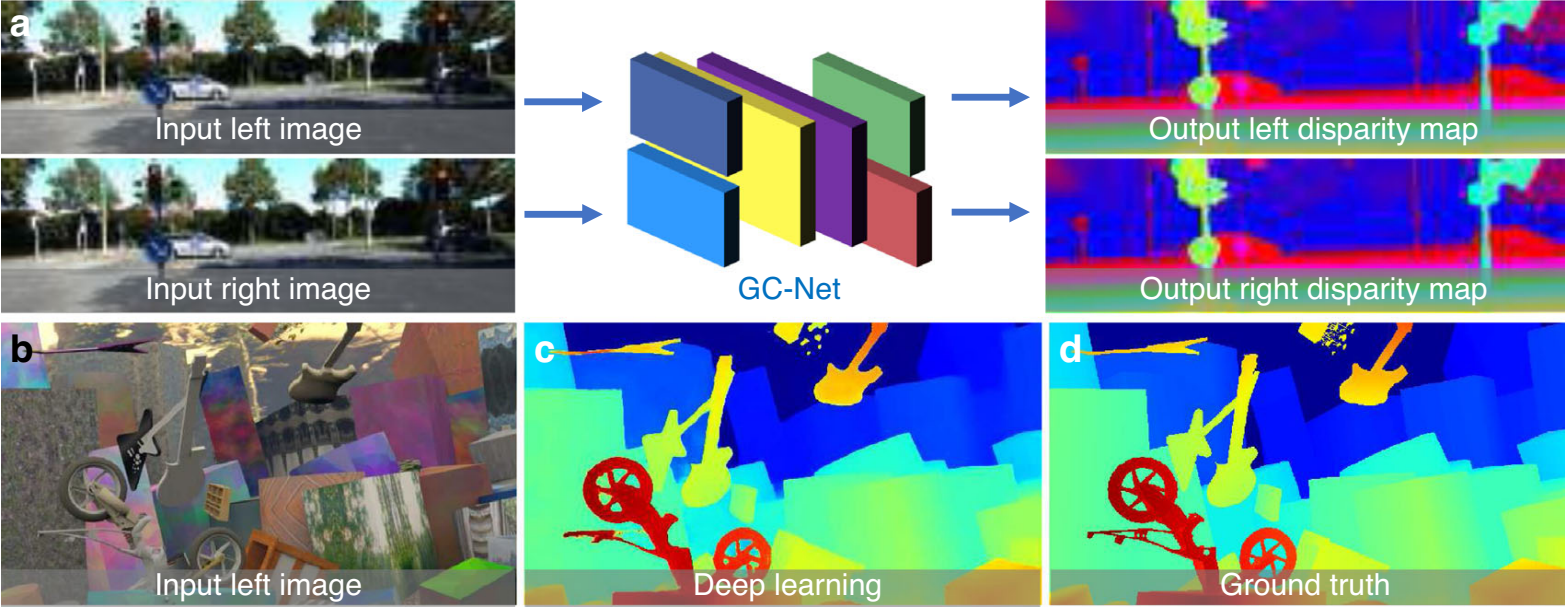
Flowchart of the deep-learning-based end-to-end disparity prediction method and the predicted disparity map result. **a** The Flowchart of the deep-learning-based end-to-end disparity prediction method: stereo images are fed into the constructed GC-Net to directly output disparity images of two perspectives. **b** The left input. **c** The disparity predicted by deep learning. **d** Ground truth. **a**–**d** ©(2021) IEEE. Adapted, with permission, from ref. ^389^

**Fig. 28:**
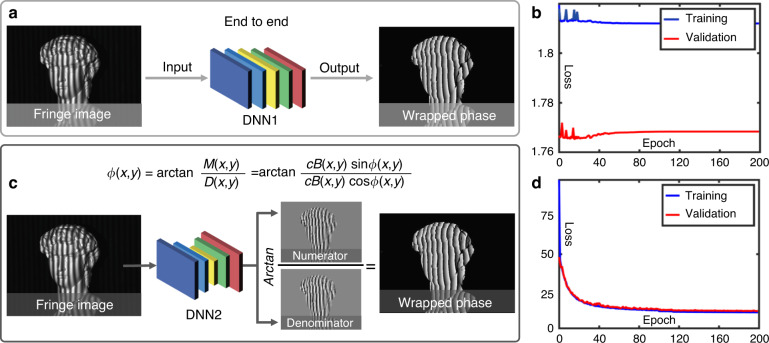
Performance comparison between the fringe-to-phase linked deep-learning method and the deep-learning approach combining the physical model of the phase-shifting method. **a** For the end-to-end network structure, the fringe image can be fed into DNN1 to directly output the corresponding wrapped phases. **b** However, such an end-to-end approach makes the training process fails to converge because it is difficult to follow the 2*π* phase truncation. **c** Our group proposed to incorporate the physical model of the traditional phase-shifting method into deep learning and applied deep learning to predict from the fringe image the numerator and denominator of the arctangent function used to calculate the phase information^50^. **d** Such a physics-informed strategy results in a stable convergence to the minimum training and validation loss. **b**, **d** Adapted with permission from ref. ^50^. Distributed under Creative Commons (CC BY 4.0) license https://creativecommons.org/licenses/by/4.0/legalcode

**Fig. 29:**
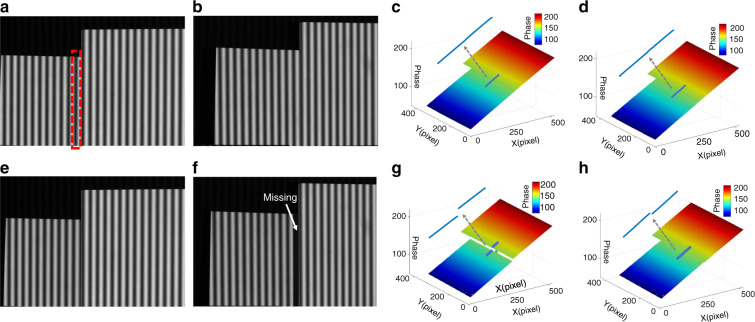
The well-trained deep-learning model for stereophase unwrapping will fail when there is depth ambiguity in a certain perspective^332^. **a** The left fringe image input of two flat plates (no ambiguity). **b** The right fringe-image input of two flat plates (no ambiguity). **c** Absolute phase map of (**a**) (ground truth). **d** Absolute phase map of (**a**) obtained by deep-learning method. **e** The left fringe pattern input of two flat plates, where the surface discontinuity leads to the absence of fringe orders (the fringe in the red dotted box in **a**) but visually presents the illusion of continuity. **f** The right fringe pattern input of two flat plates. The absence of a fringe order can be seen from this perspective. **g** Absolute phase map of (**e**) (ground truth). **h** Absolute phase map of (**e**) obtained by the deep-learning method. **a**–**h** Adapted with permission from ref. ^332^, AIP Publishing

**Fig. 30:**
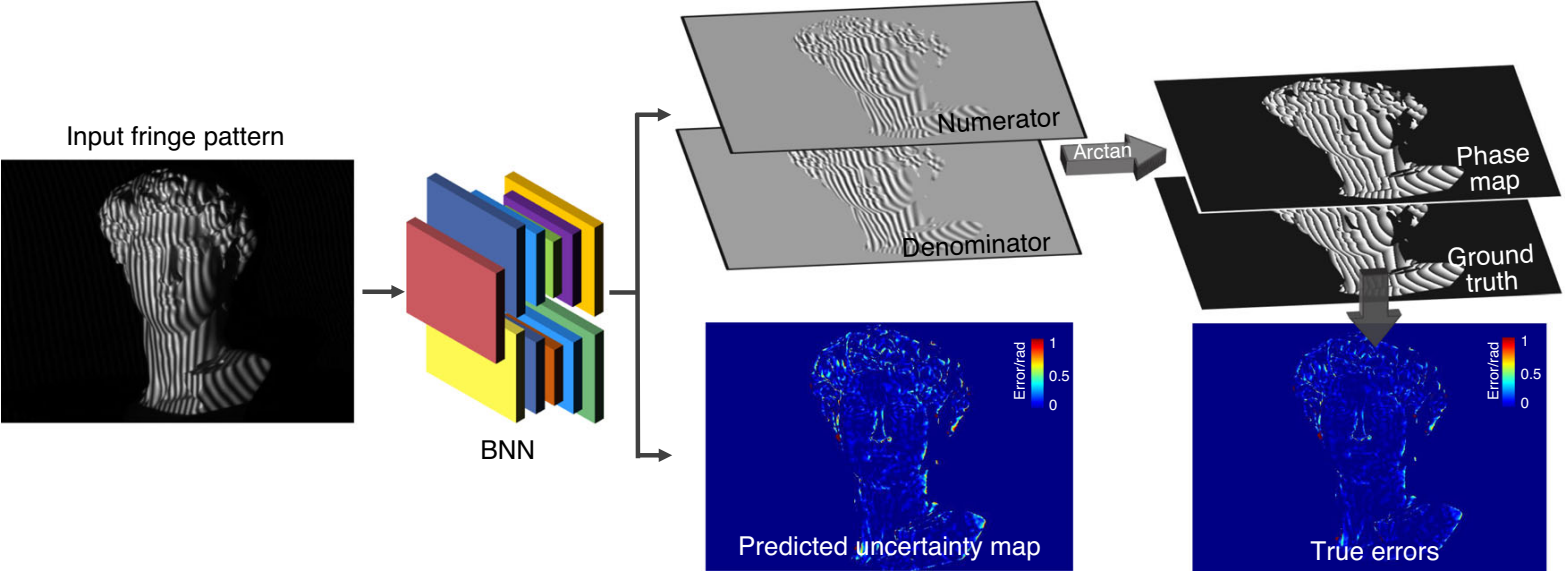
Phase retrieval uncertainty quantification based on Bayesian deep learning. Through BNN, the uncertainty map associated with a predicted wrapped phase map can be obtained, which gives direct information about the phase error

**Fig. 31:**
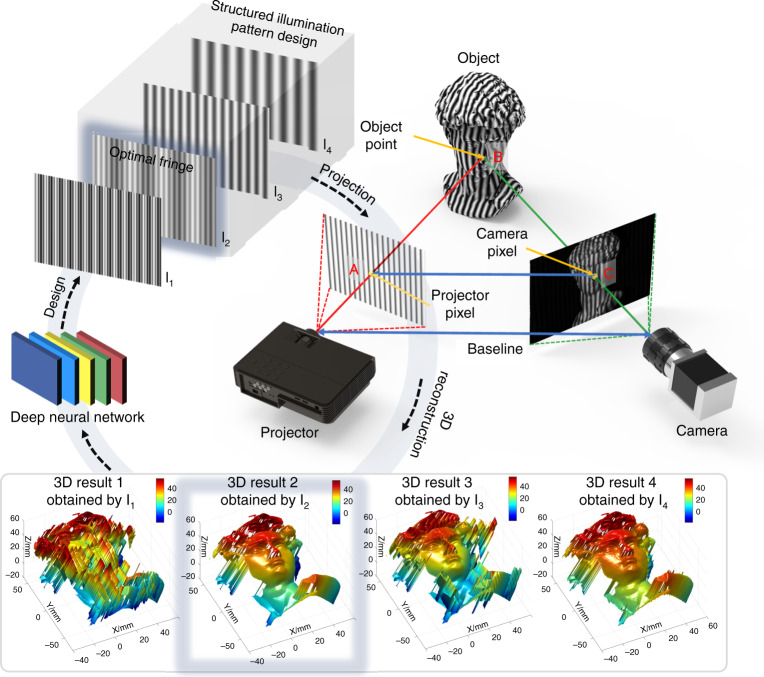
Optimization of structured light pattern for single-shot 3D shape measurement based on deep learning. Deep neural network allows to determine which pattern design can yield the optimal 3D reconstruction results

**Fig. 32:**
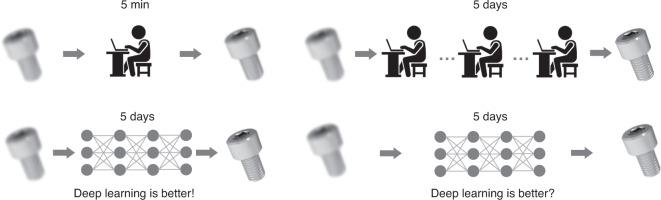
Comparison between deep learning and traditional algorithms should be objective. For several problems where traditional methods based on physics models, if implemented properly, can deliver straightforward and more than satisfying solutions, there is no need to use deep learning. However, sometimes this kind of “unnecessary” may not be recognized easily
  In DIC and stereophotogrammetry, image analysis aims to determine the displacement vector of each pixel point between a pair of acquired images. Recently, deep learning has also been extensively applied to stereomatching in order to achieve improved performance compared with traditional subset correlation and subpixel refinement methods.
  **Subset Correlation:** Zbontar and LeCun^334^ presented a deep-learning-based approach for estimating the disparity map from a rectified stereo image pair. A siamese-structured CNN was reconstructed to address the matching cost computation problem through learning the similarity measure from small image patches. The output of CNN was utilized for initializing the stereomatching cost, followed by some postprocessing processes, as shown in Fig. 20a. Figure 20d–h is the disparity images obtained from the traditional Census transform method^335^ and the deep-learning-based method, from which we can see that the deep-learning-based approach achieved a lower error rate and better prediction result. Luo et al.^336^ exploited a siamese CNN connected by point product layer to speed up the calculation of matching score and obtained improved matching performance. Recently, our group improved Luo’s network by introducing additional residual blocks and convolutional layers to the head of the neural network and replacing the original inner product with the fully connected layers with shared weights^337^. The improved network can extract a more accurate initial absolute disparity map from speckle image blocks after epipolar correction, and showed better matching capability than Luo’s network. Hartmann et al.^338^ constructed a CNN with five siamese branches to learn a matching function, which could directly predict a scalar similarity score from multiple image patches. It should be noted that the siamese CNN is one of the most widely used network structures in stereovision applications, which has been frequently employed and continuously improved for subset correlation tasks^339–343^. On a different note, Guo et al.^344^ improved the 3D-stacked hourglass network to obtain the cost volume by group-wise correlation and then realized stereomatching. Besides conventional supervised learning approaches, unsupervised learning was also introduced to subset correlation. Zhou et al.^345^ proposed an unsupervised deep-learning framework for learning the stereomatching cost, using a left-right consistency check to guide the training process to converge to a stable state. Kim et al.^346^ constructed a semi-supervised network to estimate stereo confidence. First, the matching probability was calculated according to the matching cost with residual networks. Then, the confidence measure was estimated based on a unified deep network. Finally, the confidence feature of the disparity map is extracted by synthesizing the results obtained by the two networks.
  **Subpixel refinement**: Pang et al.^347^ proposed a cascade (two-stage) CNN architecture for subpixel stereomatching. Figure 21a shows the flowchart of their method. In the first stage, the disparity image with more details was obtained from the input stereo images through DispFulNet (“Ful” means full resolution) equipped with extra upsampling modules. Then the initialized disparity was rectified and the residual signals across multiple scales were generated through the hourglass structure DispResNet (“Res” means residual) in the second stage. According to the combination of the outputs from the two stages, the final disparity with subpixel accuracy can be obtained. Figure 21d–g shows the predicted disparity images and error distributions of the input stereo image pairs (Fig. 21b) obtained by DispFulNet and DispResNet. It can be seen from the experimental results that after the second stage of optimization, the quality of the disparity was significantly improved. Based on different considerations, a large variety of network structures were proposed for subpixel refinement, e.g., StereoNet^348^, LGC-Net^349^, DeepMVS^350,351^, StereoDRNet^352^, DeepPruner^353^, LAF-Net^354^, 3D CNN^355^, MADNet^356^, Unos^357^, left-right comparative recurrent model^358^, CNN-based disparity map optimization^359^, deep-learning-based fringe-image-assisted stereomatching method^360^, and UltraStereo^361^.
3. **Postprocessing**: Deep-learning techniques also play an important role in the final postprocessing stage of the image-processing architecture of optical metrology. Examples of applying deep learning for postprocessing are very diverse, including further optimization of the measurement results (e.g., phase denoising, error compensation, and refocusing) and converting the measured intermediate variable to the desired physical quantity (e.g., system calibration and phase-to-height mapping in FPP).

- **Denoising**: Montrésor et al.^362^ proposed to use DnCNN for phase denoising. As illustrated in Fig. 22a, the sine and cosine components of the noisy phase map were fed into a DnCNN to produce the corresponding denoised version, and the resultant phase information was calculated by the arctangent function. The phase was then fed back into and refined by DnCNN again, and this process was repeated several times to achieve a better denoising performance. In order to generate more realistic training datasets via simulation, the additive amplitude-dependent speckle noise was carefully modeled by taking its non-Gaussian statistics, non-stationary properties, and a correlation length into account. Figure 22b–e shows the comparison of the denoising results obtained by WFT^114^ and the deep-learning methods, suggesting that DnCNN yielded comparable standard deviation but lower peak-to-valley phase error than WFT. Yan et al.^363^ proposed a CNN-based wrapped phase denoising method. By filtering the original numerator and denominator of the arctangent function, phase denoising results can be achieved without tuning any parameters. They also presented a deep-learning-based phase denoising technique for digital holographic speckle pattern interferometry^364^. Their approach could obtain an enhanced wrapped phase map by significantly suppressing the speckle noise, and outperformed traditional phase denoising methods when processing phases with steep spatial gradients.
- **Digital refocusing**: Ren et al.^365^ proposed the holographic reconstruction network (HRNet) to deal with the holographic reconstruction problem, which could perform automatic digital refocusing without employing any prior knowledge. Figure 23a gives the schematic of their deep-learning workflow, where a hologram input (the first block) was fed into HRNet, and then the reconstructed image (the third block) corresponding to the specific input was directly predicted. A typical lens-free Mach-Zehnder interferometer was constructed to acquire training input images, and traditional convolution method^366^, PCA aberration compensation^226^, manual artifacts removal, and phase unwrapping^367^ were successively employed to obtain the corresponding label images. Figure 23b–f shows the results of refocusing and hologram reconstruction with different methods, proving that the predicted images by HRNet were precisely in-focus and noise-free, whereas there are significant noises and artifacts in the reconstruction results obtained by traditional convolution and angular spectrum method^368^. Alternatively, the autofocusing problem in DH could be recast as a regression problem, with the focal distance being a continuous response corresponding to a digital hologram. Ren et al.^369^ constructed a CNN to achieve nonparametric autofocusing for digital holography, which could accurately predict the focal distance without knowing the physical parameters of the optical imaging system. Lee et al.^370^ constructed a CNN-based estimator combined with Discrete Fourier Transform (DFT) to realize the automatic focusing of off-axis digital holography. Their method can automatically determine the object-to-image distance rapidly and effectively, and a sharp in-focus image of the object can be reconstructed accurately. Shimobaba et al.^371^ used the regression-based CNN for holographic reconstruction, which could directly predict the sample depth position with millimeter accuracy from the power spectrum of the hologram. Jaferzadeh et al.^372^ proposed a regression-layer-toped CNN to determine the optimal focus position for numerical reconstruction of micro-sized objects, which can be extended to the study of biological samples such as cancer cells. Pitkäaho et al.^373^ constructed a CNN based on AlexNet and VGG16 to learn the defocus distances from a large number of holograms. The well-trained network can determine the high-accuracy in-focus position of a new hologram without resorting to conventional numerical propagation algorithms.
- **Error compensation**: Nguyen et al.^374^ proposed a phase-aberration compensation framework combining CNN and Zernike polynomial fitting, as illustrated in Fig. 24a. The unwrapped phase aberration map of the hologram was fed into a CNN with the U-Net structure to detect the background regions, which were then sent into the Zernike polynomial fitting^375^ to determine the conjugated phase aberration. For training data collection/preparation, the PCA method^226^ was used for training data collection/preparation. Figure 24b–e gives the phase aberration compensation results of PCA and the deep-learning method, showing that the phase aberrations were completely eliminated by using the deep-learning technique, while they were still visible in the phase results obtained by the PCA method. In addition, the deep-learning-based technique was fully automatic, and the robustness and accuracy were shown to be superior to PCA. Lv et al.^376^ used DNN to compensate projector distortion-induced measurement errors in a FPP system. By learning the mapping between the 3D coordinates of the object and their corresponding distortion-induced error distribution, the distortion errors of the original test 3D data can be accurately predicted. Aguenounon et al.^377^ leveraged a DNN with a double U-Net structure to provide the single snapshot of optical properties imaging with the additional function of real-time profile correction. The real-time visualization of the resulting profile-corrected optical property (absorption and reduced scattering) map has the potential to be deployed to guide surgeons.
- **Quantity transformation**: Li et al.^378^ proposed an accurate phase-height mapping approach for fringe projection based on a “shallow” (3-layer) BP neural network. The flowchart of their method is shown in Fig. 25a, where the camera image coordinates (*X*_*ci*_, *Y*_*ci*_) and their corresponding horizontal ones *X*_*pi*_ of the projector image were fed into the network to predict the desired 3D information (*X*_*i*_, *Y*_*i*_, *Z*_*i*_). To obtain the training data, a standard calibration board with circle marks fixed on a high-precision displacement stage was captured at different *Z*-direction positions. With the captured images, the marks’ centers coordinates (*X*_*ci*_, *Y*_*ci*_)with subpixel accuracy were extracted with the conventional circle center detection algorithm^379^, and the horizontal coordinates *X*_*pi*_ of the corresponding projector image for each mark center were calculated through the absolute phase value. Figure 25b shows the 3D reconstruction result of a standard stair sample predicted by the neural network. Figure 25c and d shows the error distributions of the measurement results obtained by traditional phase-height conversion method^380^ and neural network, showing that the learning-based method was insensitive to the fringe intensity nonlinearity and could recover the 3D shape of a workpiece with high accuracy.

### End-to-end learning in optical metrology

As mentioned earlier, “divide and conquer” is a core idea of solving complex optical metrology problems by breaking the whole image-processing pipeline into several modules or sub-steps. On a different note, deep learning enables direct mapping between the original input and the desired output, and the whole process can be trained as a whole, in an end-to-end fashion. Although somewhat brute-force, such a straightforward treatment has been extensively used in deep learning, and gradually introduced to many subfields of optical metrology, e.g., FPP and DIC.

- **From fringe to 3D shape:** In FPP, the imaging processing pipeline generally consists of pre-processing, phase demodulation, phase unwrapping, and phase-to-height conversion. Deep learning provides a viable and efficient way to reconsider the whole problem from a holistic perspective, taking human intervention out of the loop and solving the “fringe to 3D shape” problem in a purely data-driven manner. Based on this idea, Nguyen et al.^381^ proposed an end-to-end neural network to directly perform the mapping from a fringe pattern to its corresponding 3D shape, the flowchart of which is shown in Fig. 26a. Three different deep CNNs, including FCN, autoencoder^299^, and U-Net, were trained based on the datasets obtained by the conventional multi-frequency phase-shifting profilometry method. Figure 26b, c gives an input and its corresponding ground-truth 3D shape. Figure 26c shows the best 3D reconstruction results predicted by the three networks with the depth measurement accuracy of 2*mm*. Van et al.^382^ presented an SRCNN-based DNN to directly extract absolute height information from a single-fringe image. Through simulated fringe and depth image pairs, the trained network was able to obtain high-accuracy full-field depth information from a single-fringe pattern. Recently, they compared the effect of different loss functions (MAE, MSE, and SSIM) on a modified U-Net for mapping a fringe image to the corresponding depth, and designed a new mixed gradient loss function that yielded higher-quality 3D reconstructions than conventional ones^383^. Machineni et al.^384^ constructed a CNN with multiresolution similarity assessment to directly reconstruct the object’s shape from the corresponding deformed fringe image. Their proposed method can achieve promising results under various challenging conditions such as low SNR, low fringe density, and high dynamic range. Zheng et al.^385^ utilized the calibration matrix from a real-world FPP system to construct its “digital twin”, which provided abundant simulation data (fringe pattern and corresponding depth map) required for the model training. The trained U-Net can then be employed to the real-world FPP system to extract the 3D geometry encoded in the fringe pattern in one step. Similarly, Wang et al.^386^ constructed a virtual FPP system for the training dataset generation. A modified loss function based on SSIM index was employed, providing improved performance in terms of measurement accuracy and detail preservation.
- **From stereo images to disparity:** Deep learning can also be applied to DIC and stereophotogrammetry to bypass all intermediate image-processing steps in the pipeline for displacement and 3D reconstruction. Mayer et al.^387^ presented end-to-end networks for the estimation of disparity (DispNet) and optical flow (FlowNet). In DispNet, a 1D correlation was proposed along the disparity line corresponding to the stereo cost volume. In addition, they also offered a large synthetic dataset, Scene Flow^388^, for training large-scale stereomatching networks. Kendall et al.^389^ established an end-to-end Geometry and Context Network (GC-Net) mapping from a rectified pair of stereo images to disparity maps with subpixel accuracy (Fig. 27a). Stereo images were fed into the network to directly output disparity images of two perspectives. Figure 27b–d shows the test results on Scene Flow, where Fig. 27b is the left input, Fig. 27c is the disparity predicted by deep learning, and Fig. 27d is the ground truth. Experimental results show that the end-to-end learning method produced high-resolution disparity images and could tolerate large occlusions. Chang et al.^390^ developed a pyramid stereomatching network (PSMNet) to enhance the matching accuracy by using the 3D CNN-based spatial pyramid pooling and multiple hourglass networks. Zhang et al.^391^ proposed a cost aggregation network incorporating the local guided filter and semi-global-matching-based cost aggregation, achieving higher matching quality as well as better network generalization. Recently, our group proposed an end-to-end speckle correlation strategy for 3D shape measurement, where a multiscale residual subnetwork was utilized to obtain feature maps of stereo speckle images, and the 4D cost volume at one-fourth of the original^392^. Besides, a saliency detection network was integrated to generate a pixel-wise mask to exclude the shadow-noised regions. Nguyen et al.^393^ used three U-Net-based networks to convert a single speckle image into its corresponding 3D information. It should be mentioned that stereophotogrammetry is a representative field that deep learning has been extensively applied. Many other end-to-end deep-learning structures directly mapping stereo images to disparity have been proposed, such as hybrid CNN-CRF models^394^, Demon (CNN-based)^395^, MVSNet (CNN-based)^396^, CNN-based disparity estimation through feature constancy^397^, Segstereo^398^, EdgeStereo^399^, stereomatching with explicit cost aggregation architecture^400^, HyperDepth^401^, practical deep stereo (PDS)^402^, RNN-based stereomatching^403,404^, and unsupervised learning^405–409^. For DIC, Boukhtache et al.^410^ presented an enhanced FlowNet (so-called StrainNet) to predict displacement and strain fields from pairs of deformed and reference images of a flat speckled surface. Their experimental results demonstrated the feasibility of the deep-learning approach for accurate pixel-wise subpixel measurement over full displacement fields. Min et al.^411^ proposed a 3D CNN-based strain measurement method, which allowed simultaneous characterization in spatial and temporal domains from the surface images obtained during a tensile test of BeCu thin film. Rezaie et al.^412^ compared the performance of conventional DIC method and their deep-learning method based on U-Net for detecting cracks on stone masonry wall images, showing that the learning-based method could detect most visible cracks and better preserve the crack geometry.

It should be mentioned that, not just limited to phase or correlation measurement techniques, deep learning has also been widely adopted in many other fields of optical metrology. However, due to space limitations, it is not possible to describe or discuss all of them. Examples include but are not limited to the time of flight (ToF)^413–418^, photometric stereo^419–425^, wavefront sensing^426–429^, aberrations characterization^430^, and fiber optic imaging^431–435^, etc.

After reviewing hundreds of recent works leveraging deep learning for different optical metrology tasks, readers may still be interested to know to apply these new data-driven approaches to their own problems or projects. To help the reader, we present a step-by-step guide to applying deep learning to optical metrology in the Supplementary Information, taking phase demodulation from a single-fringe pattern as an example. We explain how to build a DNN with fully convolutional network architectures and train it with the experimentally collected training dataset. We also distribute the source code and the corresponding datasets for this example. Based on this example, we demonstrate that a well-trained DNN can accomplish the phase-demodulation task in an accurate and efficient manner, using only a single-fringe pattern as input. Thus, it is capable of combining the single-frame strength of the spatial phase demodulation methods with the high-measurement accuracy of the temporal phase-demodulation methods. The interested reader may refer to the Supplementary Information for the step-by-step tutorial.

## Deep learning in optical metrology: challenges

Our review in the last section shows that the deep-learning solutions in optical metrology are straightforward, but have led to improved performance compared with the state-of-the-art. In this session, we will shift our attention to reveal some challenges of the use of deep learning in optical metrology, which require further attention and careful consideration:

- **High cost of collecting and labeling experimental training data**: Most of the deep-learning techniques reviewed belong to supervised learning, which requires a large amount of labeled data to train the network. To account for real experimental conditions, deep-learning approaches can benefit from large amounts of experimental training data. Since these data serve as ground truth with sufficiently high accuracy, they are usually expensive to collect^436^. In addition, since the optical metrology system is highly customized, training data collected by one system may not be suitable for another system of the same type. This may explain why there were far fewer publicly available datasets in the field of optical metrology (especially compared with the computer vision community). Without such public benchmark datasets, it is difficult to make a fair and standardized comparison between different algorithms. Although some emerging machine learning approaches, such as transfer learning^437^, few-shot learning^438^, unsupervised learning^244^, and weak-supervised learning^439^), can decrease the reliance on the amount of data to some extent, their performance is not comparable to that of supervised learning with large data numbers so far.
- **Ground truth inaccessible for experimental data**: In many areas of optical metrology, e.g., fringe or phase denoising, it is infeasible or even impossible to get the actual ground truth of the experimental data. As discussed in previous sections, generating a training dataset by simulating the forward image formation process can bypass this difficulty^362,385^, often at the price of compromised actual performance when the knowledge of the forward image formation model ${{{\mathcal{A}}}}$ is imprecise or simulated dataset fails to reflect the real experimental system realistically and comprehensively. An alternative approach to this issue is to create a “quasi-experimental” dataset by collecting experimental raw data and then using the conventional state-of-the-art solutions to get the corresponding labels^308–310^. Essentially, the network is trained to “duplicate” the approximate inverse operator $\tilde {{{\mathcal{A}}}}^{ - 1}$ corresponding to the conventional algorithm that is used to generate the labels. After training, the network is able to emulate the conventional reconstruction algorithm $\widehat {{{{\mathcal{R}}}}_\theta }\left( {{{\mathbf{I}}}} \right) \approx {{{\mathrm{ }}}}\tilde {{{\mathcal{A}}}}^{ - 1}\left( {{{\mathbf{I}}}} \right)$, but the improvement in performance over conventional approaches becomes an unreasonable expectation.
- **Empiricism in network design and training:** So far, there is no standard paradigm for selecting appropriate DNN architectures because it requires a comprehensive understanding of the topology, training methods, and other parameters. In practice, we usually determine our network structure by evaluating different available candidate models, or comparing similar task-specific models by training them with different hyperparameters settings (network layers, neural units, and activation functions) on a specific validation dataset^440^. However, the overwhelming number of deep-learning models often limits one to evaluating only a few of the most trustworthy models, which may lead to suboptimal results. Therefore, one should learn how to quickly and efficiently narrow down the range of available models to find those most likely to be best performing on a specific type of problem. In addition, training a DNN is generally laborious and time-consuming, and becomes even worse with repetitive adjustments in the network architecture or hyperparameters to prevent overfitting and convergence issues.
- **Lack of generalization ability after specific sample training**: The generalization ability of deep-learning approaches is closely related to the size and diversity of training samples. Generally, deep-learning architectures used in optical metrology are highly specialized to a specific domain, and they should be implemented with extreme care and caution when solving issues that do not pertain to the same domain. Thus, we cannot ignore the risk that when a never-before-experienced input differs even slightly from what they encountered at the training stage, the mapping $\widehat {{{{\mathcal{R}}}}_\theta }$ established by deep networks may quickly stop making sense^441^. This is quite different from the traditional optical metrology solutions in which the reliability of the reconstruction can be secured for diverse types of samples as long as “the forward model ${{{\mathcal{A}}}}$ is accurate” and “the corresponding reconstruction algorithm $\tilde {{{\mathcal{A}}}}^{ - 1}$ is effective”.
- **“Deep learning in computer vision” ≠ “Deep learning in optical metrology”**: Deep learning is essentially the process of using computers to help us find the underlying patterns within the training dataset. Since the information cannot be “born out of nothing”, DNNs cannot always produce a provably correct solution. Compared with many computer vision tasks, optical metrology concerns more on accuracy, reliability, repeatability, and traceability^442^. For example, surface defect inspection is an indispensable quality-control procedure in manufacturing processes^443^. When using deep learning for optical metrological inspection, one may face the risk that a defect in an industrial component is “smoothed out” and undetected by an overfitted DNN in the inspection stage, which will make the entire production run defective. Since the success of deep learning depends on the “common” features learned and extracted from the training samples, which may lead to unsatisfactory results when facing “rare samples”.
- **“Deep learning” lacks the ability of “deep understanding”**: The “black box” nature of DNNs, which is arguably one of their most well-known disadvantages, prevents us from knowing how the neural network generates expected results from specific inputs by learning a large amount of training data. For example, when we send a fringe pattern into a neural network, and it outputs a poor phase image, it is not easy to comprehend what makes it arrive at such a prediction. Interpretability is critical in optical metrology because it ensures the traceability of the mistake. Consequently, most researchers in optical metrology community use deep-learning approaches in a pragmatic fashion without the possibility to explain why it provides good results or without the ability to explain the logical bases and apply modifications in the case of underperformance.

## Deep learning in optical metrology: future directions

Although the above challenges have not been adequately addressed, optical metrology is now surfing the wave of deep learning, following a trend similarly being experienced in many other fields. This field is still young, but is expected to play an increasingly prominent role in the future development of optical metrology, especially with the evolution of computer science and AI technology.

- **Hybrid, composite**, **and automated learning**: It must be admitted that at this stage, deep-learning methods for optical metrology are still limited to some elementary techniques. There is further untapped potential as a number of latest innovations in deep learning can be directly introduced into the context of optical metrology. (1) Hybrid learning methods, such as semi-supervised^242^, unsupervised^244^, and self-supervised learning^444^, are capable of extracting valuable insights from unlabeled data, which is extremely attractive as the availability of ground-truth or labeled data in optical metrology is very limited. For example, GANs utilize two networks in a competitive manner, generator and discriminator, to deceive each other during the training process to generate the final prediction without specific labels^266^. In stereovision, the network models trained by unsupervised methods have been shown to produce better disparity prediction results in real scenes^345^. (2) Composite learning approaches attempt to combine different models pretrained on a similar task to produce a composite model with improved performance^437^ or search for the optimal network architecture in the reinforcement learning environment for a certain dataset^445^. They are premised on the idea that a singular model, even very large, cannot outperform a compositional model with several small models/components, each being delegated to specialize in part of the task. As optical metrology tasks are getting more and more complicated, composite learning can deconstruct one huge task into several simpler, or single-function components and make them work together, or against each other, producing a more compressive and powerful model. (3) Automated machine learning (AutoML) approaches, such as Google AutoML^446^ and Azure AutoML^447^, is developed to execute tedious modeling tasks that once performed by professional scientists^440,448^. It burns through an enormous number of models and the associated hyperparameters on the raw input data to decide what model is best applied to it. Consequently, AutoML is expected to permit even “citizen” AI scientists with their background in optical metrology to make streamlined use cases by only utilizing their domain expertise, offering practitioners a competitive advantage with minimum investments.
- **Physics-informed deep learning**: Unlike traditional physics-model-based optical metrology methods for which the domain knowledge is carefully engineered into solutions, most of the current deep-learning-based optical metrology methods do not benefit so much from such prior knowledge but rather learn the solution from scratch by making use of massive training data. In contrast, if the physics laws governing the image formation (the knowledge about the forward image formation model ${{{\mathcal{A}}}}$) are known—even partially, they should be naturally incorporated into the DNN model so that the training data and network parameters are not wasted on “learning the physics”. For example, in fringe analysis, inspired by the conventional phase-shifting techniques, Feng et al.^50^ proposed to learn the sine and cosine components of the fringe pattern, based on which the wrapped phase can be calculated by the arctangent function (Fig. 28c, d). This method shows a significant gain in performance than directly using an end-to-end network structure^50^ (Fig. 28a, b). Goy et al.^302^ suggested a method for low-photon count phase retrieval where the noisy input image was converted into an approximant. As the approximant obtained by prior knowledge is much closer to the final prediction than the raw low-photon image, the phase reconstruction accuracy by using deep learning can be improved significantly. Wang et al.^449^ incorporated the diffraction model of numerical propagation into a DNN for phase retrieval. By minimizing the difference between the actual input image and the predicted input image, DNN learns how to reconstruct the phase that best matches the measurements without any ground-truth data.
- **Interpretable deep learning**: As we have already highlighted in the previous sections, most researchers in optical metrology use deep-learning approaches intuitively without the possibility to explain why it produces such “good” results. This can be very problematic in high-stakes settings such as industrial inspection, quality control, and medical diagnose where the decisions of algorithms must be explainable, or where accountability is required. Academics in deep learning are acutely aware of this interpretability problem, and there have been several developments in recent years for visualizing the features and representations they have learned by DNNs^284^. On the other hand, often applied to high-risk scenarios, optical metrology is among the most significant deep-learning challenges—we are dealing with unknown, uncertain, ambiguous, incomplete, noisy, inaccurate, and missing datasets in high-dimensional spaces. The unexplainability and incomprehensibility of deep learning also imply the predictions are at risk of failure. Figure 29 illustrates one such example, where a well-trained deep-learning model for stereophase unwrapping fails when there exists depth ambiguity in a certain perspective^332^. Therefore, explainability will become a key strength in deep-learning techniques to interpret and explain models, which would significantly expand the usefulness of deep-learning methods in optical metrology.
- **Uncertainty quantification**: Characterizing uncertainty in deep-learning solutions can help make better decisions and take precautions against erroneous predictions, which is essential for many optical metrology tasks^450^. However, most deep-learning methods reviewed in this work cannot provide uncertainty estimates. In recent years, Bayesian deep learning has emerged as a unified probabilistic framework that tightly integrates deep learning with Bayesian models^451^. By using a GAN training framework to estimate a posterior distribution of images fitting a given measurement dataset (or estimation statistics derived from the posterior), Bayesian convolutional neural networks (BNNs) can quantify the reliability of predictions through two predictive uncertainties, including model uncertainty and data uncertainty, akin to epistemic and revelation uncertainty in Bayesian analysis, respectively^452^. It is expected to be adopted in optical metrology applications, e.g., fringe pattern analysis, to give pixel-wise variance estimates and data uncertainty evaluation (Fig. 30)^453^. The latter further allows assessment of the randomness of predictions stemming from data imperfections, including noise, incompleteness of the training data, and other experimental perturbations. Incorporating similar uncertainty quantification into other deep-learning-based optical metrology methods, especially when the ground truth is unavailable, is an interesting direction for future research.
- **Guiding the metrology system design**: Most of the current work using deep learning in optical metrology only considers how to reconstruct the measured data as a postprocessing algorithm while ignoring the way how the image data should be formed. However, an important feature of optical metrology methods is their active nature, especially with respect to the way of manipulating the illumination. For example, in FPP, the structure of the illumination is modulated systematically throughout the object surface to deliver high accuracy and robustness in establishing the triangulation. The design of the illumination coding strategy is curial to improving the measurement accuracy removing the ambiguity of the depth reconstruction with a minimum number of image acquisitions. However, this problem has long been tackled using heuristics like composite coding, frequency multiplexing, and color multiplexing, which does not guarantee optimality (in terms of facilitating the recovery of desired information). Deep learning provides a mechanism to optimize the system design in a more principled way. By integrating the image formation model (with trainable parameters controlling the image acquisition) into the reconstruction network, the system design and the reconstruction algorithm (i.e., both ${{{\mathcal{A}}}}$ and the corresponding $\widehat {{{{\mathcal{R}}}}_\theta }$) can be jointly optimized with the training data^454^. It allows us to determine which type of system design can yield the best results for a particular deep-learning-driven task. Such an idea has been successfully demonstrated in designing optimal illumination patterns for computational microscopes^455–457^. We hope that this “joint optimization” network can effectively bridge the gap between how images should be acquired and how these images should be post-processed by deep learning, and can be widely adopted in designing the optical metrology systems, such as the fringe pattern design in FPP (Fig. 31), and the speckle pattern design in DIC, etc.
- **Both “deep” and “in-depth”:** Should we use deep learning or traditional optical metrology algorithms? It is a tough question to answer because it depends heavily on the problem to be solved. Considering the “no free lunch theorem”, the selection between deep-learning and traditional algorithms should be considered rationally. For several problems where traditional methods based on physics models, if implemented properly, can deliver straightforward and more than satisfying solutions, there is no need to use deep learning. However, sometimes this kind of “unnecessary” may not be recognized easily. While being functionally effective, we should keep in mind that “how best deep learning can do” generally depends on “how reliable the training data we can provide.” For example, though the popular “learning from simulation” scheme used in optical metrology eliminates the dependence on huge labeled experimental data, the inconsistency between the image formation model and actual experimental condition leads to additional challenges of “domain adaptation”. Therefore, our personal view is that deep learning does not (at least at the current stage) make our research easier. On the contrary, it raises the threshold for optical metrology research because it requires researchers not only need to use and understand deep learning deeply but also need to take “in-depth” research in traditional algorithms so as to make an impartial and objective assessment between deep learning and traditional optical metrology algorithms (Fig. 32).

## Conclusions

A brief summary of this review indicates that there has been significant interest in the advancement of optical metrology technologies using deep-learning architectures. The rapid development of deep-learning technology has led to a paradigm shift from physics- and knowledge-based modeling to data-driven learning for solving a wide range of optical metrology tasks. In general, deep learning is particularly advantageous for many problems in optical metrology whose physical models are complicated and acquired information is limited, e.g., in harsh environments and many challenging applications. Strong empirical and experimental evidence suggests that using problem-specific deep-learning models outperforms conventional knowledge or physical model-based approaches.

Despite the promising—in many cases pretty impressive—results that have been reported in the literature, potential problems and challenges remain. For model training, we need to acquire large amounts of experimental data with labels, which, even if available, is laborious and requires professional experts. We have been looking for the theoretical groundwork that would clearly explain the mechanisms and ways to the optimal selection of network structure and training algorithm for a specific task, or to profoundly comprehend why a particular network structure or algorithm is effective in a given task or not. Furthermore, deep-learning approaches have often been regarded as “black boxes”, and in optical metrology, accountability is essential and can cause severe consequences. Combining Bayesian statistics with deep neuron networks to obtain quantitative uncertainty estimates allows us to assess when the network yields unreliable predictions. A synergy of the physics-based models that describe the a priori knowledge of the image formation and data-driven models that learn a regularizer from the experimental data can bring our domain expertise into deep learning to provide more physically plausible solutions to specific optical metrology problems. Leveraging these emerging technologies in the application of deep-learning methods to optical metrology could promote and accelerate the recognition and acceptance of deep learning in more application areas. These are among the most critical issues that will continue to attract the interest of deep-learning research in the optical metrology community in the years to come.

In summary, although for different optical metrology tasks, deep-learning techniques can bring substantial improvements compared to traditional methods, the field is still at the early stage of development. Many researchers are still skeptical and maintain a wait-and-see attitude towards its applications involving industrial inspection and medical care, etc. Shall we accept deep learning as the key problem-solving tool? Or should we reject such a black-box solution? These are controversial issues in the optical metrology community today. Looking on the bright side, it has promoted an exciting trend and fostered expectations of the transformative potential it may bring to the optical metrology society. However, we should not overestimate the power of deep learning by considering it as a silver bullet for every challenge encountered in the future development of optical metrology. In practice, we should assess whether the large amount of data and computational resources required to use deep learning for a particular task is worthwhile, especially when other conventional algorithms may yield comparable performance with lower complexity and higher interpretability. We envisage that deep learning will not replace the role of traditional technologies within the field of optical metrology for the years to come, but will form a cooperative and complementary relationship, which may eventually become a symbiotic relationship in the future.

## Supplementary information

Supplementary Information

Supplemental Material File #1
