# Correction: Deep learning in optical metrology: a review

**DOI:** 10.1038/s41377-022-00757-0

**Published:** 2022-03-27

**Authors:** Chao Zuo, Jiaming Qian, Shijie Feng, Wei Yin, Yixuan Li, Pengfei Fan, Jing Han, Kemao Qian, Qian Chen

**Affiliations:** 1 Smart Computational Imaging (SCI) Laboratory, Nanjing University of Science and Technology, 210094 Nanjing, Jiangsu Province China; 2 Jiangsu Key Laboratory of Spectral Imaging & Intelligent Sense, Nanjing University of Science and Technology, 210094 Nanjing, Jiangsu Province China; 3 School of Engineering and Materials Science, Queen Mary University of London, London, E1 4NS UK; 4 School of Computer Science and Engineering, Nanyang Technological University, Singapore, 639798 Singapore

**Keywords:** Imaging and sensing, Optical metrology

Correction to: *Light: Science & Applications*

10.1038/s41377-022-00714-x, published online 23 February 2022

Following publication of this article^1^, it is noticed that some brackets are missing in the mathematical expressions in Fig. 1. Updated Fig. 1 is provided in this Correction.
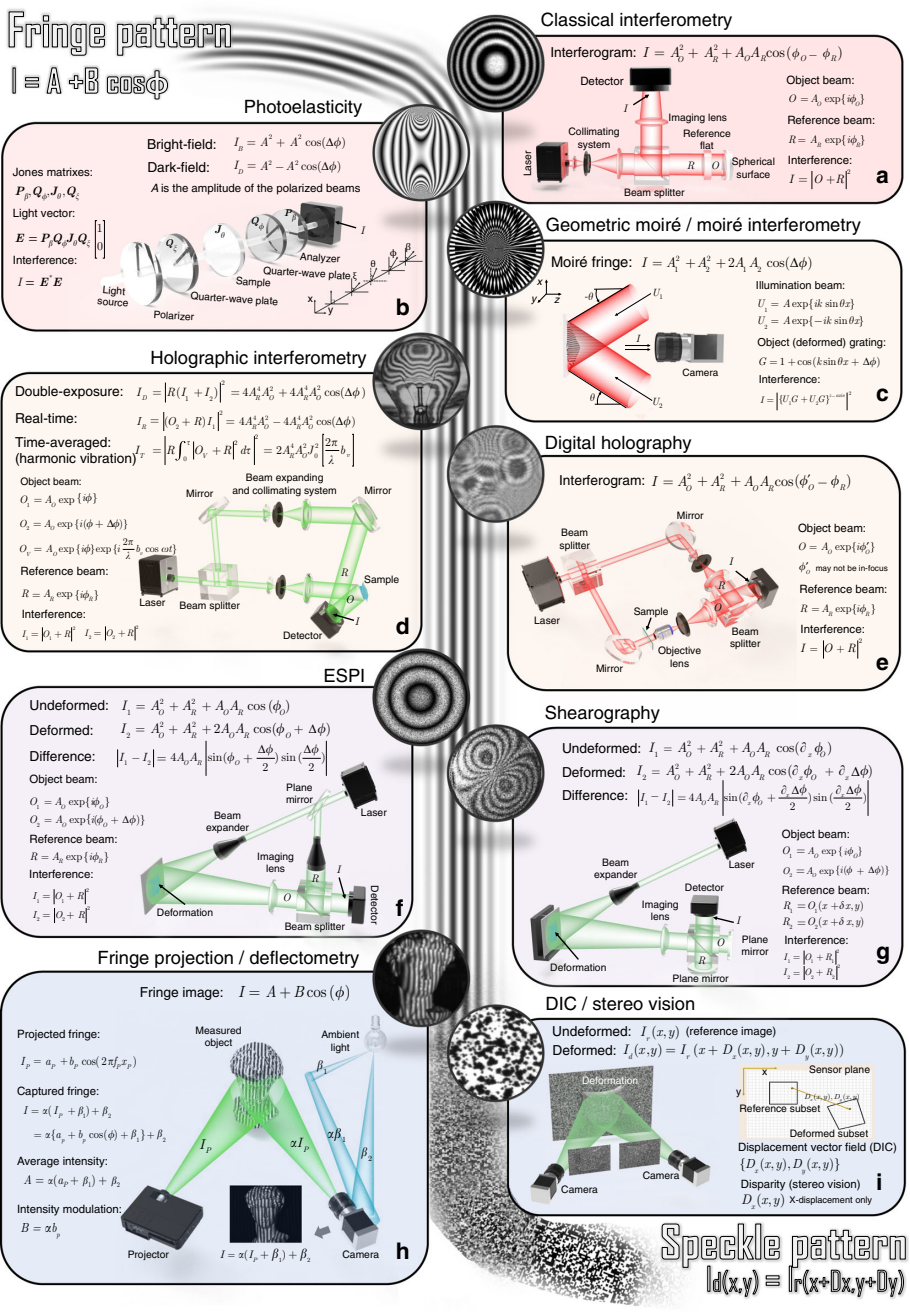


The original article has been updated.
