# Supplementary material for: Deep learning in optical metrology: a review
Source: Light Sci Appl. 2022 Feb 23;11:39. doi: 10.1038/s41377-022-00714-x (PMC8866517; doi:10.1038/s41377-022-00714-x)
Supplement: Supplementary file 1 — Supplementary Information [file 41377_2022_714_MOESM1_ESM.pdf]

Supplementary Information:

Supplementary Information for Deep learning in optical metrology: a review

Chao Zuo[1,2,*,+], **Jiaming Qian**[1,2,+], **Shijie Feng**[1,2], **Wei Yin**[1,2], **Yixuan Li**[1,2], **Pengfei Fan**[1,3], **Jing Han**[2], **Kemao Qian**[4,**], **and Qian Chen**[2,***]

[1]Smart Computational Imaging (SCI) Laboratory, Nanjing University of Science and Technology, Nanjing, Jiangsu Province 210094, China
[2]Jiangsu Key Laboratory of Spectral Imaging & Intelligent Sense, Nanjing University of Science and Technology, Nanjing, Jiangsu Province 210094, China
[3]School of Engineering and Materials Science, Queen Mary University of London, London E1 4NS, UK.
[4]School of Computer Science and Engineering, Nanyang Technological University, Singapore 639798, Singapore
[*]zuochao@njust.edu.cn
[**]mkmqian@ntu.edu.sg
[***]chenqian@njust.edu.cn
[+]these authors contributed equally to this work

ABSTRACT

This document provides supplementary information for "Deep learning in optical metrology: a review". We present a step-by-step guide to applying deep learning to optical metrology, taking phase demodulation from a single fringe pattern as an example.

Contents:

A. Overview of phase demodulation using deep learning
B. Design and implementation
C. Result analysis
D. Supplementary code and data

A. Overview of phase demodulation using deep learning

For many phase measuring optical metrology techniques, including optical interferometry, digital holography, electronic speckle pattern interferometry, Moiré profilometry, and fringe projection profilometry, the physical quantities to be measured (such as the surface shape, displacement, strain, roughness, defects, *etc.*) are directly or indirectly encoded in the phase information of the fringes formed by means of interference or projection. Consequently, phase demodulation, which analyzes the quasi-periodic fringe pattern for the wrapped phase extraction, is the most critical step because the measurement accuracy of these optical metrology techniques depends directly on the phase demodulation accuracy of recorded fringe patterns. How to extract the phase information with the highest accuracy, fastest speed, and full automation remains a research hotspot in the field of optical metrology.

For many phase measuring optical metrology techniques, including optical interferometry, digital holography, electronic speckle pattern interferometry, Moiré profilometry, and fringe projection profilometry, the physical quantities to be measured (such as the surface shape, displacement, strain, roughness, defects, *etc.*) are directly or indirectly encoded in the phase information of the fringes formed by means of interference or projection. Consequently, phase demodulation, which analyzes the quasi-periodic fringe pattern for the wrapped phase extraction, is the most critical step because the measurement accuracy of these optical metrology techniques depends directly on the phase demodulation accuracy of recorded fringe patterns. How to extract the phase information with the highest accuracy, fastest speed, and full automation remains a research hotspot in the field of optical metrology.

As discussed in the Section *"Image processing in optical metrology"* of the main text, traditional fringe pattern analysis, or phase demodulation techniques, can be broadly classified into two categories: spatial phase demodulation and temporal phase demodulation:

- Temporal phase demodulation techniques detect the phase distribution from the temporal variation of fringe signals[1]. The most well-established phase-shifting technique extracts the phase information using multiple phase-shifted fringe patterns achieving high-resolution pixel-wise phase measurement at the cost of time-sequential data acquisitions. Consequently, such approaches are vulnerable to disturbances such as object motion/environmental vibration, thus making them difficult to be applied to high-speed measurements.

- Spatial phase demodulation methods, such as Fourier transform (FT)[2], windowed Fourier transform (WFT)[3], and wavelet transform (WT)[4] methods, are capable of estimating the phase distribution from a single fringe pattern, making them insensitive to vibration/motion and appropriate for dynamic measurement. However, they are sensitive to fringe discontinuities, isolated samples, and rich details of testing surfaces, preventing them from high-precision and high-resolution phase measurement of complex surfaces. In addition, spatial phase demodulation methods usually have many parameters to adjust in order to achieve better results, making them difficult to be fully automatic in practical applications.

The goal of this *Supplementary Information* is to present a simple but representative example of how to apply deep learning to optical metrology. More specifically, you will learn how to build a deep neural network (DNN) with fully convolutional network architectures (U-Net) and how to train the network for phase demodulation from a single fringe pattern. Instead of adopting an end-to-end learning scheme directly linking the input fringe image to the output phase map, here we choose to **predict the numerator and denominator terms of the arctangent function of the phase map from one input fringe pattern [Fig. S1]** based on two basic considerations: (1) predicting the phase from the arctangent function bypasses the difficulties associated with reproducing abrupt 2π phase wraps, and thus, obtains a high-quality phase estimate; (2) such a strategy refers to the physical model of the traditional phase-shifting method, which removes the influence of the surface reflectivity by the division operation, making the trained model suitable for objects with complex surfaces[5,6].It will be demonstrated that the developed and trained deep neural network can accomplish the phase demodulation task in an accurate and efficient manner, using only a single fringe pattern. Thus, it is capable of combining the single-frame strength of the spatial phase demodulation methods with the high measurement accuracy of the temporal phase demodulation methods.

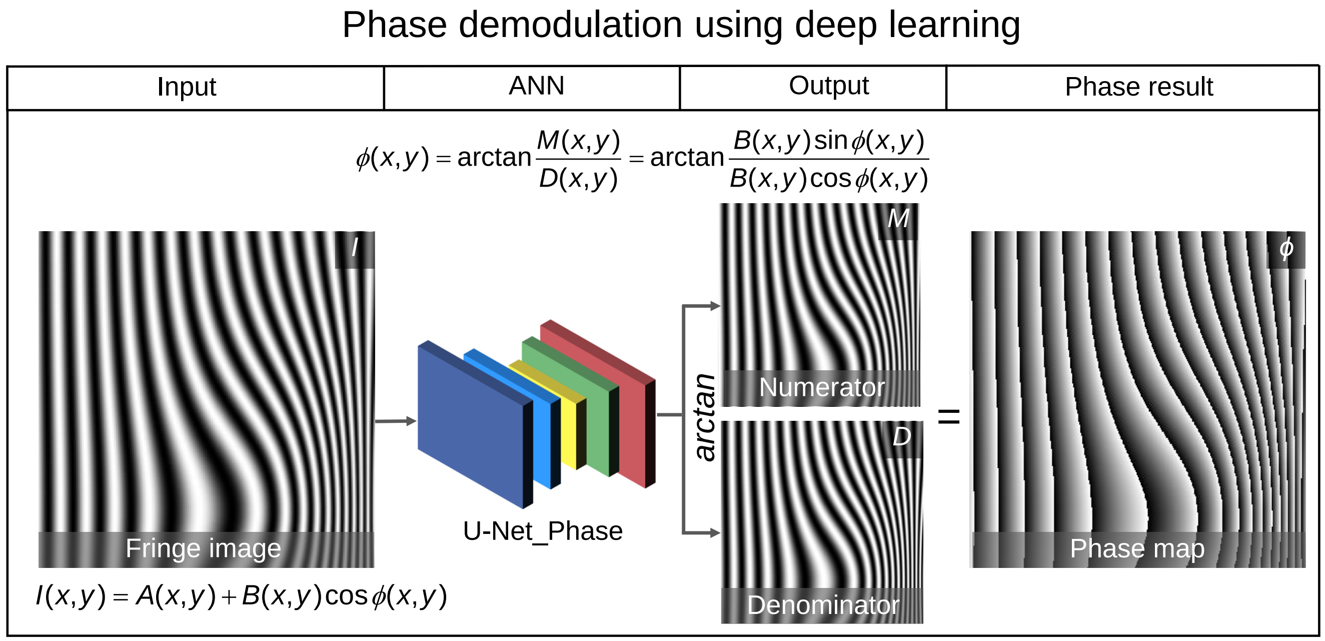

Figure S1. **Flowchart of the fringe analysis using deep neural networks.** With a fringe image as input, a convolutional network (we call it U-Net_Phase) can estimate two parts: sine (M) and cosine (D) part. These two parts as the numerator and denominator are then fed into the arctangent function for the final phase calculation

B. Design and implementation

As summarized in Fig. S2, the workflow of phase demodulation using deep learning comprises three main steps: **(1) designing an appropriate network architecture; (2) collecting training datasets by experiments; (3) training the network until its loss converges**.

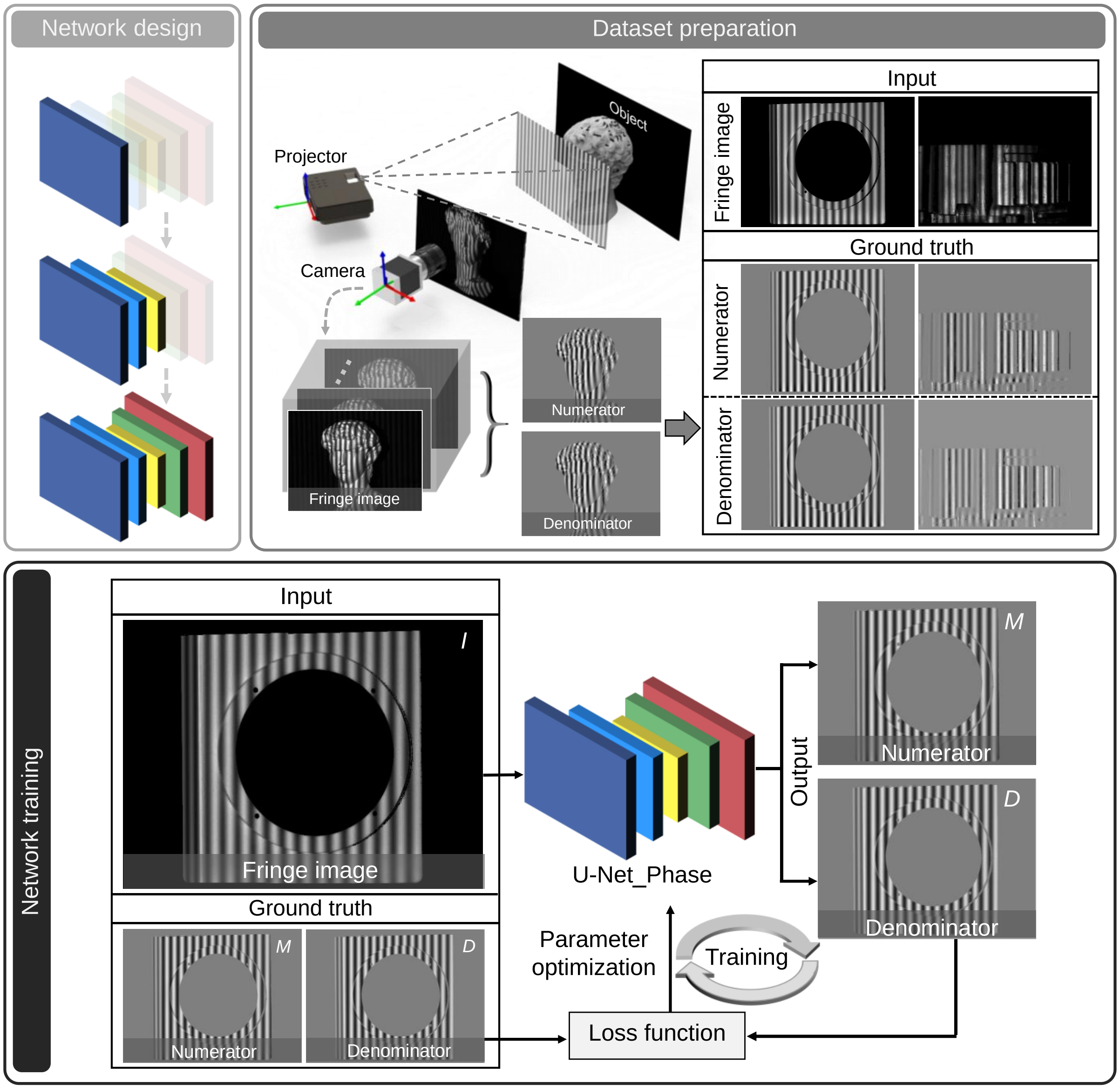

Figure S2. Process of phase demodulation using deep learning.

Source codes for this tutorial in the *"Supplementary code"* folder. In order to run the source codes, the user must navigate to the work directory (main folder *"Supplementary code"*). The main folder contains two Python file (*.py) [Fig. S3]:

- step1_train_phase_demodulation.py: the source codes for training U-Net_Phase;
- step2_test_phase_demodulation.py: the source codes for testing U-Net_Phase.

The sample code provided here was computed on a desktop (Linux system) with Intel Core i7-7800X CPU and a GeForce GTX 1080 Ti GPU (NVIDIA) under the Python 3.6 deep learning framework Keras 2.2.4 with the TensorFlow 1.10.1 platform (Google), and was run in Spyder 5.0.5, which is an open-source cross-platform integrated development environment for scientific computing using the Python language.

The datasets for the phase demodulation task have been uploaded to the figshare (`https://figshare.com/s/f150a36191045e0c1bef`) [Fig. S4]. In this example, the data in the following two subfolders are used:

- Input of CNN1: The input data are in this folder and named "LeftImage";
- Output of CNN1: The corresponding ground truth data are in this folder, and the numerator and denominator are named "M" and "D", respectively.

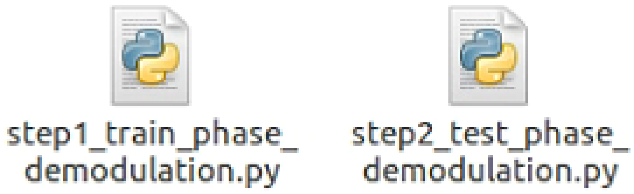

Figure S3. **Source codes for training and testing deep learning for phase demodulation.**

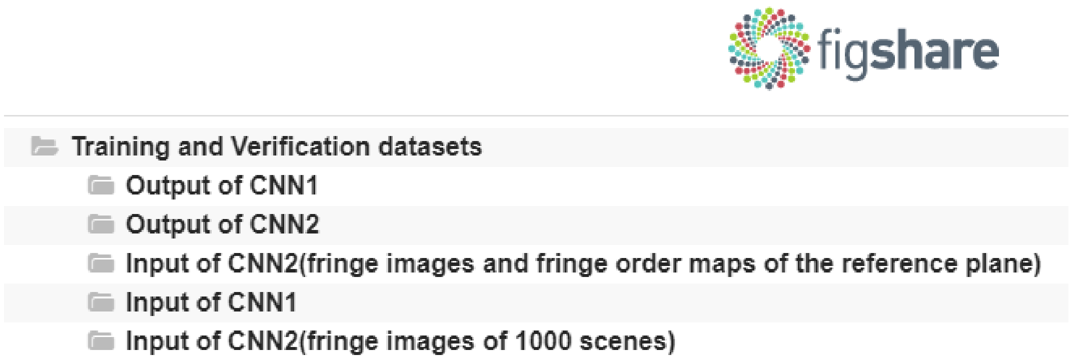

Figure S4. **Datasets for the phase demodulation task.**

Network architecture design

In principle, any fully convolutional network architectures can be used for different types of image processing tasks that we encountered in optical metrology. Just as we discussed in the Subsection *"Advantages of invoking deep learning in optical metrology"*, deep learning subverts the conventional "physics-model-driven" paradigm and opens up the "data-driven" learning-based representation paradigm, which eliminates the need to design different processing flows for specific image processing algorithm based on experience and pre-knowledge. **By applying different types of training datasets, one specific class of neural network can be trained to perform various types of transformation for different tasks, significantly improving the universality and reducing the complexity of solving new problems.**.

In this regard, we adopt a typical fully convolutional network architecture—U-Net[7] to predict the numerator and denominator terms of the arctangent function of the phase map from one input fringe pattern. Considering the task that U-Net aims at, we call the designed network **U-Net_Phase** here. Its detailed inner architecture is shown in Fig. S5. A 3D tensor with size (H,W,C_0) is used as the input of the network, where (H,W) is the size of the input images, and C_0 represents the number the input images, which is one in this case. For each convolutional layer, the kernel size is 3×3 with convolution stride one, zero-padding is used to control the spatial size of the output, and the output is a 3D tensor of shape (H,W,C), where C = 64 represents the number of filters used in each convolutional layer.

- In the first path of U-Net_Phase, the input is processed by a convolutional layer, followed by a group of residual blocks (containing four residual blocks) and another convolutional layer. Each residual block consists of 2 sets of convolutional layers activated by rectified linear unit (ReLU)[8] stacked one above the other, which can solve the degradation of accuracy as the network becomes deeper and ease the training process[9]. Also, implementing shortcuts between residual blocks contributes to the convolution stability.

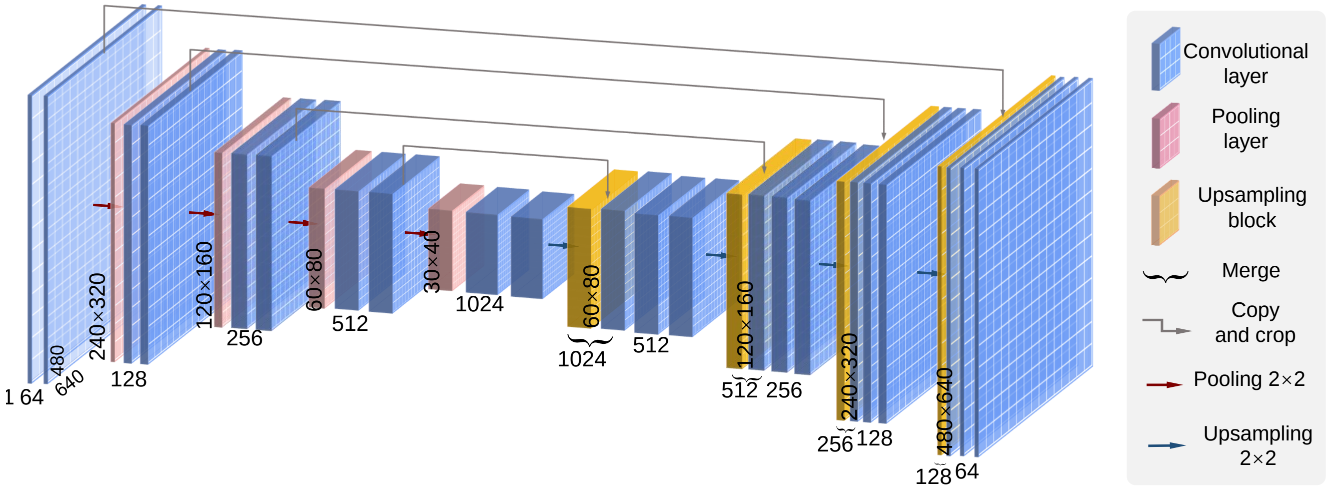

Figure S5. **The detailed structure of U-Net_Phase.**

- In the other three paths, the data is down-sampled by the pooling layers by two, four, and eight times, respectively, for better feature extraction, and then up-sampled by the upsampling blocks to match the original size. The input data passes through a convolutional layer with ReLU activation. We then use quadruple filters to extract features from the input for providing rich information for the following upsampling.

- The outputs of four paths are concatenated into a tensor with quad channels. Finally, two channels are generated in the last convolution layer.

Except for the last convolutional layer which is activated linearly, the rest ones use the ReLU as activation function, *i.e.*, $ReLU\left(x\right) = max\left(0, x\right)$. Here we use a classic loss function—the mean-squared-errors of the outputs with respect to the ground truth, which is computed as:

$$Loss(\theta) = \frac{1}{H * W}\left[\left\|P_M^\theta - G_M\right\|^2 + \left\|P_D^\theta - G_D\right\|^2\right] \tag{S1}$$

where G_M and G_M are the ground truth of the numerator and denominator, and P_M^θ and P_D^θ the numerator and denominator predicted by the neural network with the parameter space θ which includes the weights, bias and convolutional kernels in this layer. In the training, the networks use the score of loss function as a feedback signal to adjust the parameters in θ by a little bit, in a direction that would lower the loss score. To this end, the adaptive moment estimation (ADAM) is used in the network to tune the parameters to find the minimum of the loss function.

The codes on lines 188 to 238 in "step1_train_phase_demodulation.py" [Fig. S6] correspond to the structure of U-Net_Phase. Figure S7 gives an explanation of the "Conv2D" function of constructing the convolution layer, which contains some important parameters:

- num_filter_conv: the number of filters;

- num_conv: convolution kernel size;

- activation: activation function;

- padding: filling type of convolution, including "valid" and "same";

- kernel_initializer: initializer of kernel weight matrix;

Users can modify the network structure or replace the network of other structures according to their needs.

```
"""
Merge the outputs from the above passes and output the final result
"""
num_filter_conv = 64
num_conv = 3


#Block1
conv1 = layers.Conv2D(num_filter_conv, num_conv, activation = 'relu', padding = 'same', kernel_initializer = 'he_normal')(input_tensor)
conv1 = layers.Conv2D(num_filter_conv, num_conv, activation = 'relu', padding = 'same', kernel_initializer = 'he_normal')(conv1)
pool1 = layers.MaxPooling2D(pool_size=(2, 2))(conv1)
#Block2
conv2 = layers.Conv2D(num_filter_conv*2, num_conv, activation = 'relu', padding = 'same', kernel_initializer = 'he_normal')(pool1)
conv2 = layers.Conv2D(num_filter_conv*2, num_conv, activation = 'relu', padding = 'same', kernel_initializer = 'he_normal')(conv2)
pool2 = layers.MaxPooling2D(pool_size=(2, 2))(conv2)
#Block3
conv3 = layers.Conv2D(num_filter_conv*4, num_conv, activation = 'relu', padding = 'same', kernel_initializer = 'he_normal')(pool2)
conv3 = layers.Conv2D(num_filter_conv*4, num_conv, activation = 'relu', padding = 'same', kernel_initializer = 'he_normal')(conv3)
pool3 = layers.MaxPooling2D(pool_size=(2, 2))(conv3)
#Block4
conv4 = layers.Conv2D(num_filter_conv*8, num_conv, activation = 'relu', padding = 'same', kernel_initializer = 'he_normal')(pool3)
conv4 = layers.Conv2D(num_filter_conv*8, num_conv, activation = 'relu', padding = 'same', kernel_initializer = 'he_normal')(conv4)
drop4 = layers.Dropout(0.5)(conv4)
pool4 = layers.MaxPooling2D(pool_size=(2, 2))(drop4)
#Block5
conv5 = layers.Conv2D(num_filter_conv*16, num_conv, activation = 'relu', padding = 'same', kernel_initializer = 'he_normal')(pool4)
conv5 = layers.Conv2D(num_filter_conv*16, num_conv, activation = 'relu', padding = 'same', kernel_initializer = 'he_normal')(conv5)
drop5 = layers.Dropout(0.5)(conv5)
#Block6
up6 = layers.Conv2D(num_filter_conv*8, 2, activation = 'relu', padding = 'same', kernel_initializer = 'he_normal')(layers.UpSampling2D(size = (2,2))(drop5))
merge6 = layers.concatenate([drop4,up6], axis = 3)
conv6 = layers.Conv2D(num_filter_conv*8, num_conv, activation = 'relu', padding = 'same', kernel_initializer = 'he_normal')(merge6)
conv6 = layers.Conv2D(num_filter_conv*8, num_conv, activation = 'relu', padding = 'same', kernel_initializer = 'he_normal')(conv6)
#Block7
up7 = layers.Conv2D(num_filter_conv*4, 2, activation = 'relu', padding = 'same', kernel_initializer = 'he_normal')(layers.UpSampling2D(size = (2,2))(conv6))
merge7 = layers.concatenate([conv3,up7], axis = 3)
conv7 = layers.Conv2D(num_filter_conv*4, num_conv, activation = 'relu', padding = 'same', kernel_initializer = 'he_normal')(merge7)
conv7 = layers.Conv2D(num_filter_conv*4, num_conv, activation = 'relu', padding = 'same', kernel_initializer = 'he_normal')(conv7)
#Block8
up8 = layers.Conv2D(num_filter_conv*2, 2, activation = 'relu', padding = 'same', kernel_initializer = 'he_normal')(layers.UpSampling2D(size = (2,2))(conv7))
merge8 = layers.concatenate([conv2,up8], axis = 3)
conv8 = layers.Conv2D(num_filter_conv*2, num_conv, activation = 'relu', padding = 'same', kernel_initializer = 'he_normal')(merge8)
conv8 = layers.Conv2D(num_filter_conv*2, num_conv, activation = 'relu', padding = 'same', kernel_initializer = 'he_normal')(conv8)
#Block9
up9 = layers.Conv2D(num_filter_conv, 2, activation = 'relu', padding = 'same', kernel_initializer = 'he_normal')(layers.UpSampling2D(size = (2,2))(conv8))
merge9 = layers.concatenate([conv1,up9], axis = 3)
conv9 = layers.Conv2D(num_filter_conv, num_conv, activation = 'relu', padding = 'same', kernel_initializer = 'he_normal')(merge9)
conv9 = layers.Conv2D(num_filter_conv, num_conv, activation = 'relu', padding = 'same', kernel_initializer = 'he_normal')(conv9)

final_result = layers.Conv2D(2, 3,  padding = 'same', kernel_initializer = 'he_normal')(conv9)
model = Model(input = input_tensor, output = final_result)
```

Figure S6. **Codes used for constructing neural network.**

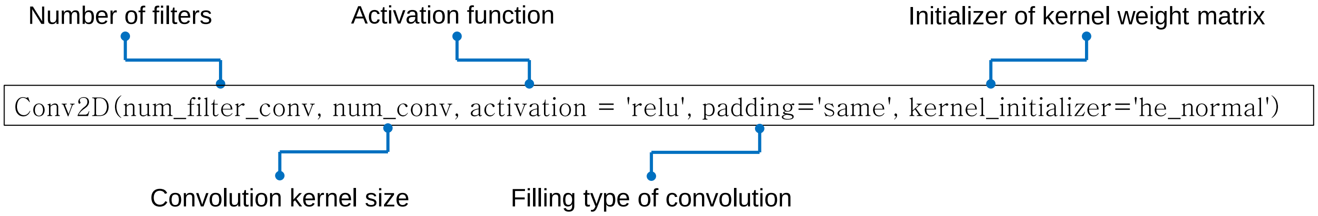

Figure S7. **Explanation of the "Conv2D" function of constructing the convolution layer.**

Dataset preparation

In order to train the network, a matched dataset of ground truth parameters and corresponding raw fringe patterns should be created. In this tutorial, the dataset was collected by physical experiments based on a real fringe projection profilometry system, which includes a LightCrafter 4500Pro (912 $\times$ 1140 resolution) and a Basler acA640-750 μm camera (640 $\times$ 480 resolution). The camera is equipped with a lens of 12 mm focal length. The distance between the measured object and our system was about one meter. To obtain the precise parameters estimates, multi-step phase-shifting algorithm is used to calculate the ground truth data for our neural networks. The multi-step phase-shifting fringe patterns are generated as

$$I_n^p(x^p, y^p) = a + b\cos\left(2\pi f x^p - \frac{2\pi n}{N}\right) \tag{S2}$$

where (x^p, y^p) is the pixel coordinate of the projector, and index $n = 0, 1, 2, ..., N-1$ (N is the number of phase-shifting steps). Parameters a, b, f are the mean value, amplitude and spatial frequency, respectively. In our experiments, we set $a = b = 127.5$ (for projection of 8-bits images) and $f = 48$. The projector projected the generated multi-step phase-shifting fringe patterns onto different measured objects. The camera captured the reflected fringe pattern simultaneously from a different angle and transferred them to our computer. As the performance of the deep neural network largely depends on the quality of collected

training data, we captured 1000 different scenes including simple and complex objects (metal industrial parts, plaster models, paper boxes, *etc.*) placed in arbitrary postures. For each scene, we recorded multi-step phase-shifting fringe patterns. The captured phase-shifting images can be represented as:

$$I_n(x,y) = A(x,y) + B(x,y)\cos[\varphi(x,y) + 2\pi n/N] \tag{S3}$$

where I_n represents the $(n+1)$th captured image, $n = 0,1,...,N-1$, (x,y) is the camera pixel coordinate, A is the average intensity map, B is the fringe amplitude map, φ is the phase, and $2\pi n/N$ is the phase shift. Through the standard N-step phase-shifting algorithm, we then calculated the corresponding ground truth data with the least square method:

$$\varphi(x,y) = \arctan\frac{M(x,y)}{D(x,y)} = \arctan\frac{\sum_{n=0}^{N-1} I_n(x,y)\sin(2\pi n/N)}{\sum_{n=0}^{N-1} I_n(x,y)\cos(2\pi n/N)} \tag{S4}$$

Through Eq. (S4), we can calculate the numerator term M and denominator term D of the arctangent function. It is worth noting that we recommend using the phase-shifting method with a higher number of steps, such as 12-step phase-shifting method, to obtain higher-quality phase-related information. Then we use the first fringe patten of the three-step phase-shifting images and the corresponding numerator and denominator terms as a set of input and ground truth data for training the neural network. The training datasets have been uploaded to the figshare (`https://figshare.com/s/f150a36191045e0c1bef`). The input data are in the "Input of CNN1" folder and named "LeftImage"; the corresponding ground truth data are in the "Output of CNN1" folder, and the numerator and denominator are named "M" and "D" respectively. Figure S8 shows four representative sets of training data. The first column show captured fringe images of the scenes. The second and third rows of Fig. S8 shows the ground truth numerator and denominator for the network training. Moreover, for a preferable selection of training objects, one is suggested choosing objects without very dark or shiny surfaces to insure captured fringe images with enough signal-to-noise ratio or without saturated points.

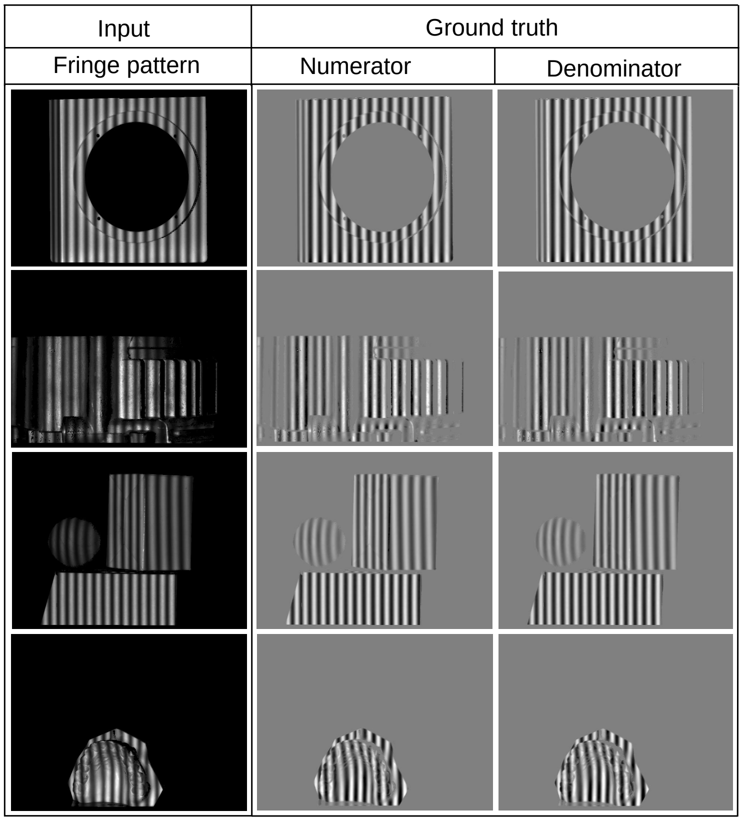

Figure S8. Four representative sets of input and ground truth data.

Network training

Before being fed into the network, the input fringe images are divided by 255 for normalization, making the learning process easier for the network. In this case, 450 sets of data are used for training and 150 sets are used for verification. The codes on lines 56 to 142 in “step1_train_phase_demodulation.py” are used to initialize the input and output tensors for training and verification [Fig. S9]. Users can customize the file names of the datasets and the amounts of data used for training and verification, and even modify different input and output data for other tasks.

```
55  ##########################################################################################
56  start = time.clock()
57  
58  total_images = 600
59  num_feature = 2
60  img_height = 640
61  img_width = 480
62  num_validation = 150
63  num_train = total_images - num_validation
64  
65  #read train_x & train_y
66  dir_base = './Input of CNN1/'
67  dir_valid = './Output of CNN1/'
68  
69  
70  # two inputs and two outputs
71  train_x = np.empty((num_train, img_height, img_width, 1),dtype="float32")
72  train_y = np.empty((num_train, img_height, img_width, 2),dtype="float32")
73  validation_x = np.empty((num_validation, img_height, img_width, 1),dtype="float32")
74  validation_y = np.empty((num_validation, img_height, img_width, 2),dtype="float32")
75  
76  """
77  step1: Disrupting the order of data
78  """
79  ind_list = [i+1 for i in range(num_train)]
80  random.shuffle(ind_list)
81  index_train = ind_list[0: num_train]
82  ind_list = [i+1 for i in range(num_validation)]
83  random.shuffle(ind_list)
84  index_validation = ind_list[0: num_validation]
85  
86  #read train_x & train_y
87  train_x1_dir = dir_base
88  train_y1_dir = dir_valid
89  train_y2_dir = dir_valid
90  
91  cnt = 0
92  for i in range(num_train):
93  
94      img_add = train_x1_dir + 'LeftImage'+str(index_train[i]) + '.mat.mat'
95      mat_train = h5py.File(img_add)
96      img = mat_train['LeftImage']
97      train_x[cnt, :, :, 0] = img[:]/255
98  
99      data_add = train_y1_dir + 'M'+ str(index_train[i]) + '.mat.mat'
100     mat_train = h5py.File(data_add)
101     parallax = mat_train['M']
102     train_y[cnt, :, :, 0] = parallax[:]
103 
104     data_add = train_y2_dir + 'D'+str(index_train[i]) + '.mat.mat'
105     mat_train = h5py.File(data_add)
106     parallax = mat_train['D']
107     train_y[cnt, :, :, 1] = parallax[:]
108 
109     cnt = cnt + 1
110 
111 print('training data has been loaded')
112 elapsed = (time.clock() - start)
113 print("Time used:",elapsed)
114 
115 #read validation_x
116 valid_x1_dir = dir_base
117 valid_y1_dir = dir_valid
118 valid_y2_dir = dir_valid
119 
120 cnt = 0
121 for i in range(num_validation):
122 
123     img_add = valid_x1_dir + 'LeftImage'+str(index_validation[i])  + '.mat.mat'
124     mat_train = h5py.File(img_add)
125     img = mat_train['LeftImage']
126     validation_x[cnt, :, :, 0] = img[:]/255
127 
128     data_add = valid_y1_dir +'M'+ str(index_validation[i]) + '.mat.mat'
129     mat_train = h5py.File(data_add)
130     parallax = mat_train['M']
131     validation_y[cnt, :, :, 0] = parallax[:]
132 
133     data_add = valid_y2_dir +'D'+ str(index_validation[i]) + '.mat.mat'
134     mat_train = h5py.File(data_add)
135     parallax = mat_train['D']
136     validation_y[cnt, :, :, 1] = parallax[:]
137 
138     cnt = cnt + 1
139 
140 print('validation data has been loaded')
141 elapsed = (time.clock() - start)
142 print("Time used for loading data:",elapsed)
```

Figure S9. Codes used for initializing the tensors for training and verification.

Then start training the network. The codes on lines 242 to 275 in “step1_train_phase_demodulation.py” are used to train the network model [Fig. S10], which defines the loss function, optimizer type, training epoch, *etc*. When “step1_train_phase_demodulation.py” file is compiled successfully in Spyder, the Spyder’s console prints the network structure table, as shown in Fig. S11. When training starts, the Spyder’s console prints the time consumption, epoch number and loss of the training, as shown in Fig. S12.

After 300 epochs, the training loss curve converges, and the network training is completed. The training of 300 epochs takes about 5.67 hours. The training and validation losses and the learned model are output as files [Fig. S13], where “phase_demodulation_model.h5” is the learned model, and “traloss_cnn1.mat” and “valloss_cnn1.mat” are training and verification loss data. The loss curve distribution of training and verification will also be printed in Spyder’s console.

```python
"""
step3: training my model
"""
epochs_num = 300
mini_batch = 2

#optimizer = optimizers.Adam(lr=0.0001)
model.summary()
model.compile(optimizer = 'Adam', loss = 'mse', metrics = ['mae'] )#rmsprop


callbacks = [keras.callbacks.ModelCheckpoint(
            filepath='./model.h5', # Path to the destination model file
            # The two arguments below mean that we will not overwrite the
            # model file unless `val_loss` has improved, which
            # allows us to keep the best model every seen during training.
            monitor='val_loss',
            save_best_only=True),

            keras.callbacks.ReduceLROnPlateau(
            monitor='val_loss',
            factor = 0.5,
            patience=10),

            keras.callbacks.TensorBoard(
            # Log files will be written at this location
            log_dir='log_integer/speckle6_integer+20')
            ]

history = model.fit(train_x, train_y, validation_data = (validation_x, validation_y),
          epochs = epochs_num, batch_size = mini_batch, verbose=1, callbacks=callbacks)

val_mae_history = history.history['val_mean_absolute_error']
mae_history = history.history['mean_absolute_error']
```

Figure S10. **Codes used for training neural network.**

Layer (type)	Output Shape	Param #	Connected to
input_1 (InputLayer)	(None, 640, 480, 1)	0	
conv2d_1 (Conv2D)	(None, 640, 480, 64)	640	input_1[0][0]
conv2d_2 (Conv2D)	(None, 640, 480, 64)	36928	conv2d_1[0][0]
max_pooling2d_1 (MaxPooling2D)	(None, 320, 240, 64)	0	conv2d_2[0][0]
conv2d_3 (Conv2D)	(None, 320, 240, 128	73856	max_pooling2d_1[0][0]
conv2d_4 (Conv2D)	(None, 320, 240, 128	147584	conv2d_3[0][0]
max_pooling2d_2 (MaxPooling2D)	(None, 160, 120, 128	0	conv2d_4[0][0]
conv2d_5 (Conv2D)	(None, 160, 120, 256	295168	max_pooling2d_2[0][0]
conv2d_6 (Conv2D)	(None, 160, 120, 256	590080	conv2d_5[0][0]
max_pooling2d_3 (MaxPooling2D)	(None, 80, 60, 256)	0	conv2d_6[0][0]
conv2d_7 (Conv2D)	(None, 80, 60, 512)	1180160	max_pooling2d_3[0][0]
conv2d_8 (Conv2D)	(None, 80, 60, 512)	2359808	conv2d_7[0][0]
dropout_1 (Dropout)	(None, 80, 60, 512)	0	conv2d_8[0][0]
max_pooling2d_4 (MaxPooling2D)	(None, 40, 30, 512)	0	dropout_1[0][0]
conv2d_9 (Conv2D)	(None, 40, 30, 1024)	4719616	max_pooling2d_4[0][0]
conv2d_10 (Conv2D)	(None, 40, 30, 1024)	9438208	conv2d_9[0][0]
dropout_2 (Dropout)	(None, 40, 30, 1024)	0	conv2d_10[0][0]
up_sampling2d_1 (UpSampling2D)	(None, 80, 60, 1024)	0	dropout_2[0][0]
conv2d_11 (Conv2D)	(None, 80, 60, 512)	2097664	up_sampling2d_1[0][0]
concatenate_1 (Concatenate)	(None, 80, 60, 1024)	0	dropout_1[0][0]<br>conv2d_11[0][0]
conv2d_12 (Conv2D)	(None, 80, 60, 512)	4719104	concatenate_1[0][0]
conv2d_13 (Conv2D)	(None, 80, 60, 512)	2359808	conv2d_12[0][0]
up_sampling2d_2 (UpSampling2D)	(None, 160, 120, 512	0	conv2d_13[0][0]
conv2d_14 (Conv2D)	(None, 160, 120, 256	524544	up_sampling2d_2[0][0]
concatenate_2 (Concatenate)	(None, 160, 120, 512	0	conv2d_6[0][0]<br>conv2d_14[0][0]
conv2d_15 (Conv2D)	(None, 160, 120, 256	1179904	concatenate_2[0][0]
conv2d_16 (Conv2D)	(None, 160, 120, 256	590080	conv2d_15[0][0]
up_sampling2d_3 (UpSampling2D)	(None, 320, 240, 256	0	conv2d_16[0][0]
conv2d_17 (Conv2D)	(None, 320, 240, 128	131200	up_sampling2d_3[0][0]
concatenate_3 (Concatenate)	(None, 320, 240, 256	0	conv2d_4[0][0]<br>conv2d_17[0][0]
conv2d_18 (Conv2D)	(None, 320, 240, 128	295040	concatenate_3[0][0]
conv2d_19 (Conv2D)	(None, 320, 240, 128	147584	conv2d_18[0][0]
up_sampling2d_4 (UpSampling2D)	(None, 640, 480, 128	0	conv2d_19[0][0]
conv2d_20 (Conv2D)	(None, 640, 480, 64)	32832	up_sampling2d_4[0][0]
concatenate_4 (Concatenate)	(None, 640, 480, 128	0	conv2d_2[0][0]<br>conv2d_20[0][0]
conv2d_21 (Conv2D)	(None, 640, 480, 64)	73792	concatenate_4[0][0]
conv2d_22 (Conv2D)	(None, 640, 480, 64)	36928	conv2d_21[0][0]
conv2d_23 (Conv2D)	(None, 640, 480, 2)	1154	conv2d_22[0][0]

Total params: 31,031,682
Trainable params: 31,031,682
Non-trainable params: 0

Figure S11. **Spyder's console prints the network structure table.**

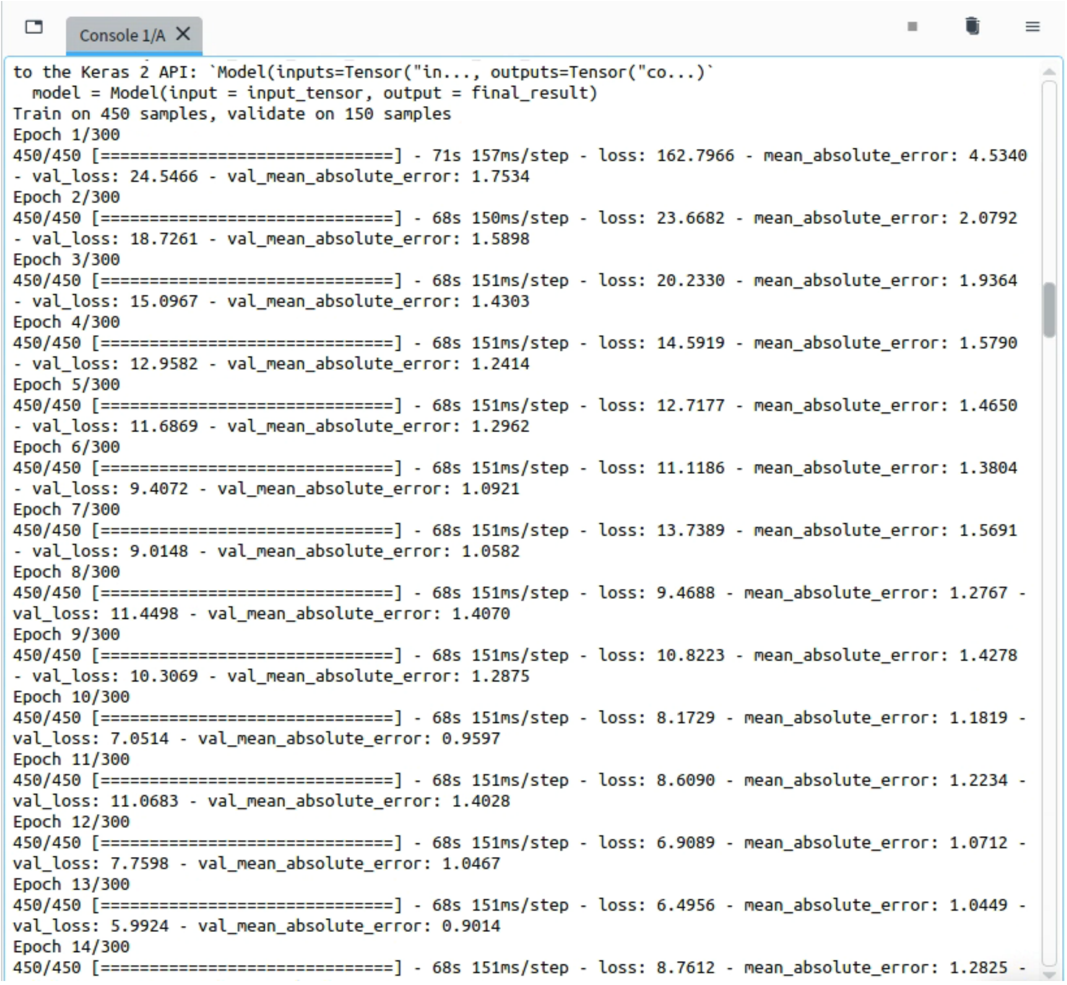

Figure S12. **Spyder's console outputs the time consumption, epoch number and loss of the training, *etc*.**

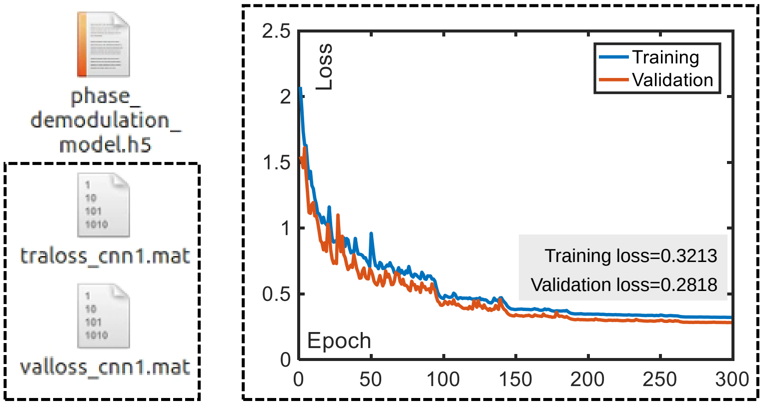

Figure S13. **The learned model, loss data, and loss curve distribution after the training is completed.**

C. Result analysis

To test the phase demodulation performance of the trained network, we apply it to phase demodulation of fringe patterns obtained by measuring different types of samples. By running "step2_test_phase_demodulation.py" in Spyder, the predicted numerator and denominator terms corresponding to the input fringe images can be output in the "test" folder (the output folder can be customized). Then users can use the arctangent function to get the phase map. Figures S14 and S15 respectively show the comparison results of different phase demodulation methods for training data and test data, where Figs. S14b-d and Figs. S15b-d are the numerator terms and denominator terms predicted from the inputs [Figs. S14a and S15a], and the phase maps calculated by the prediction results, respectively. It can be seen from Figs. S14e-S14g and Figs. S15e-S15g that, compared with the traditional single-frame phase demodulation method—spatial phase demodulation methods (here we use FT and WFT methods) whose quality is limited around discontinuities and isolated areas, the deep learning method can obtain high-quality phase map with significantly higher accuracy. We calculate the phase RMSE of traditional single-frame methods and deep learning method relative to the phase-shifting method. It can be seen from the quantization results that the phase accuracy of the deep learning method is nearly an order of magnitude higher than that of traditional single-frame methods.

To compare the results more intuitively, we further unwrap the phases and reconstruct the 3D surface profiles of the measured objects based on the pre-calibrated geometry parameters of the fringe projection system used for acquiring the dataset. It can be seen that the reconstructed result from FT [Figs. S14h and S15h] features many grainy distortions, which are mainly due to the inevitable spectral leakage and overlapping in the frequency domain. Compared with FT, the WFT reconstructed the objects with more smooth surfaces but failed to preserve the surface details, especially around the edge regions [Figs. S14i and S15i]. By comparison, the deep learning method yielded the highest-quality 3D reconstructions with the high-fidelity recovery of surface details, producing 3D reconstructions [Figs. S14j and S15j] that visually almost reproduce the results [Figs. S14k and S15k] of the multi-step phase-shifting method. But different from the multi-frame phase-shifting method, such high-quality phase demodulation is obtained from only one fringe image as input. Further, unlike the FT and WFT methods, where the performance heavily relies on fine-tuning of several parameters, the deep learning method is fully automatic — once the neural network has been trained, it does not require any manual parameter searches to optimize its performance.

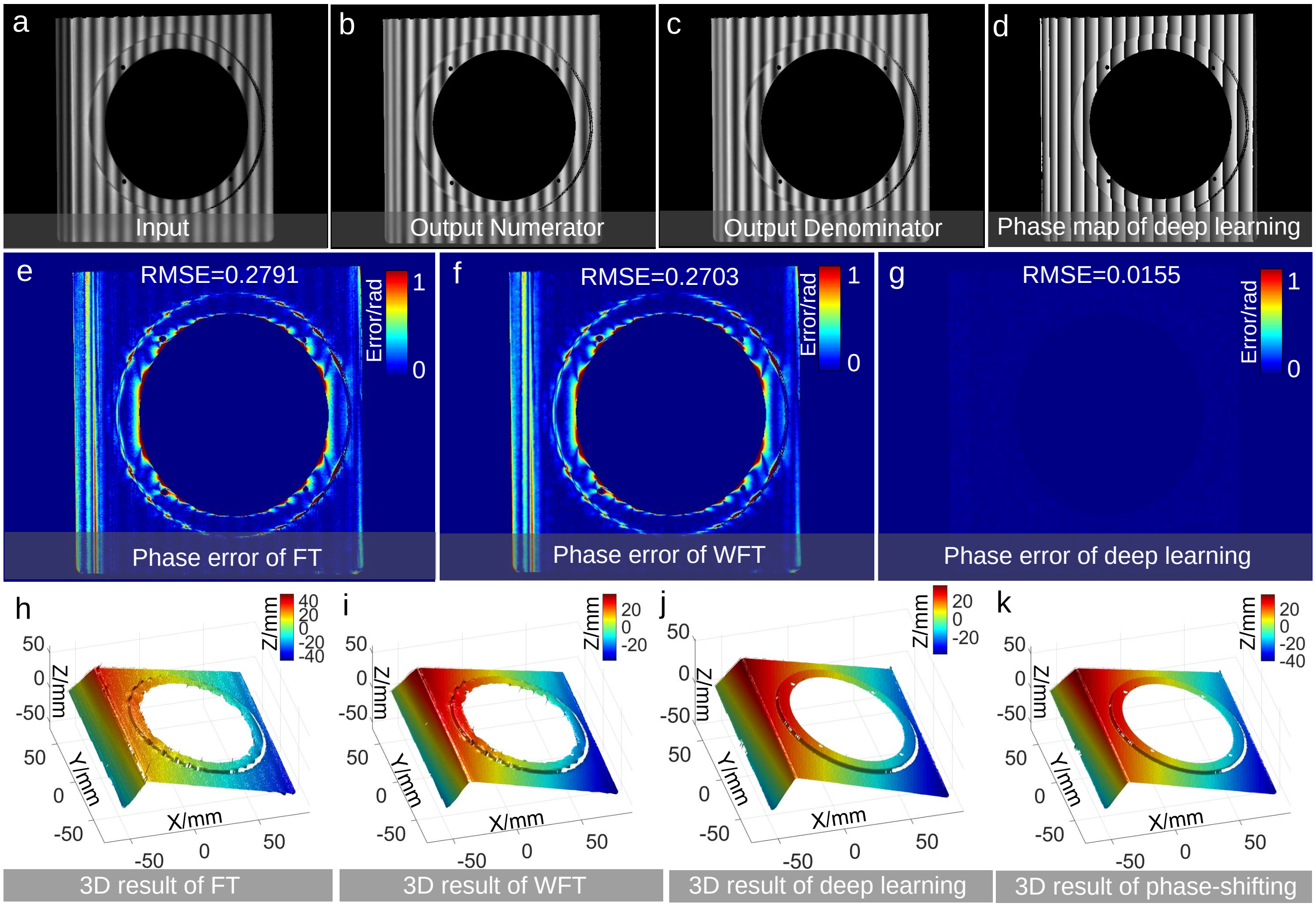

Figure S14. Comparison results of different phase demodulation methods for training data. **a** Input fringe pattern. **b** The predicted numerator. **c** The predicted denominator. **d** Phase map calculated from the numerator and denominator predicted by deep learning. **e** Phase error of FT compared to the phase-shifting method. **f** Phase error of WFT compared to the phase-shifting method. **g** Phase error of deep learning compared to the phase-shifting method. **h** 3D result of FT. **i** 3D result of WFT. **j** 3D result of deep learning. **k** 3D result of phase-shifting method.

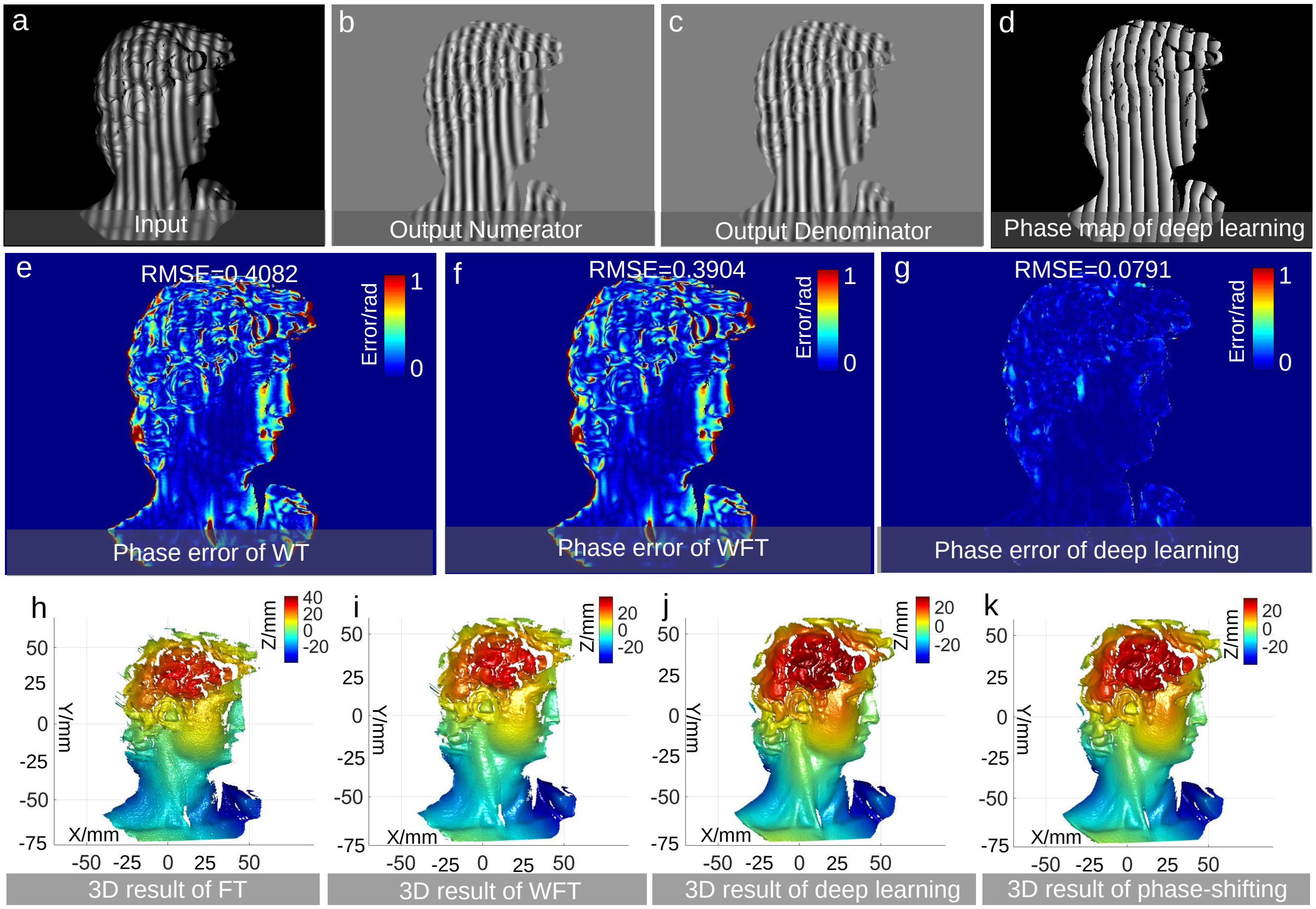

Figure S15. Comparison results of different phase demodulation methods for test data. **a** Input fringe pattern. **b** The predicted numerator. **c** The predicted denominator. **d** Phase map calculated from the numerator and denominator predicted by deep learning. **e** Phase error of FT compared to the phase-shifting method. **f** Phase error of WFT compared to the phase-shifting method. **g** Phase error of deep learning compared to the phase-shifting method. **h** 3D result of FT. **i** 3D result of WFT. **j** 3D result of deep learning. **k** 3D result of phase-shifting method

D. Supplementary code and data

For convenience, here we once again summarize the paths of the source codes and datasets associated with this tutorial. The source codes are in the main folder *"Supplementary code"*, which contains two Python file (*.py) [Fig. S3]:

- step1_train_phase_demodulation.py: the source codes for training U-Net_Phase;
- step2_test_phase_demodulation.py: the source codes for testing U-Net_Phase.

The datasets for this tutorial have been uploaded to the figshare `https://figshare.com/s/f150a36191045e0c1bef` [Fig. S4], where the following two subfolders is used in our example:

- Input of CNN1: The input data are in this folder and named "LeftImage";
- Output of CNN1: The corresponding ground truth data are in this folder, and the numerator and denominator are named "M" and "D", respectively.

References

1. Srinivasan, V., Liu, H.-C. & Halioua, M. Automated phase-measuring profilometry of 3-d diffuse objects. *Applied Optics* **23**, 3105–3108 (1984).
2. Takeda, M., Ina, H. & Kobayashi, S. Fourier-transform method of fringe-pattern analysis for computer-based topography and interferometry. *JOSA* **72**, 156–160 (1982).
3. Kemao, Q. Windowed fourier transform for fringe pattern analysis. *Applied Optics* **43**, 2695–2702 (2004).
4. Zhong, J. & Weng, J. Spatial carrier-fringe pattern analysis by means of wavelet transform: wavelet transform profilometry. *Applied Optics* **43**, 4993–4998 (2004).
5. Feng, S. *et al.* Fringe pattern analysis using deep learning. *Advanced Photonics* **1**, 025001 (2019).
6. Qian, J. *et al.* Deep-learning-enabled geometric constraints and phase unwrapping for single-shot absolute 3d shape measurement. *APL Photonics* **5**, 046105 (2020).
7. Ronneberger, O., Fischer, P. & Brox, T. U-net: Convolutional networks for biomedical image segmentation. In *International Conference on Medical Image Computing and Computer-Assisted Intervention*, 234–241 (Springer, 2015).
8. Nair, V. & Hinton, G. E. Rectified linear units improve restricted boltzmann machines. In *ICML* (2010).
9. He, K., Zhang, X., Ren, S. & Sun, J. Deep residual learning for image recognition. In *Proceedings of the IEEE Conference on Computer Vision and Pattern Recognition*, 770–778 (2016).
